# Genomic impact of stress-induced transposable element mobility in Arabidopsis

**DOI:** 10.1093/nar/gkab828

**Published:** 2021-09-22

**Authors:** David Roquis, Marta Robertson, Liang Yu, Michael Thieme, Magdalena Julkowska, Etienne Bucher

**Affiliations:** Plant Breeding and Genetic Resources, Agroscope, 1260 Nyon, Switzerland; Plant Breeding and Genetic Resources, Agroscope, 1260 Nyon, Switzerland; Boyce Thompson Institute, 533 Tower Rd., Ithaca, NY 14853, USA; Institute for Plant and Microbial Biology, University of Zurich, Switzerland; Boyce Thompson Institute, 533 Tower Rd., Ithaca, NY 14853, USA; Plant Breeding and Genetic Resources, Agroscope, 1260 Nyon, Switzerland

## Abstract

Transposable elements (TEs) have long been known to be major contributors to plant evolution, adaptation and crop domestication. Stress-induced TE mobilization is of particular interest because it may result in novel gene regulatory pathways responding to stresses and thereby contribute to stress adaptation. Here, we investigated the genomic impacts of stress induced TE mobilization in wild type Arabidopsis plants. We find that the heat-stress responsive *ONSEN* TE displays an insertion site preference that is associated with specific chromatin states, especially those rich in H2A.Z histone variant and H3K27me3 histone mark. In order to better understand how novel *ONSEN* insertions affect the plant's response to heat stress, we carried out an in-depth transcriptomic analysis. We find that in addition to simple gene knockouts, *ONSEN* can produce a plethora of gene expression changes such as: constitutive activation of gene expression, alternative splicing, acquisition of heat-responsiveness, exonisation and genesis of novel non-coding and antisense RNAs. This report shows how the mobilization of a single TE-family can lead to a rapid rise of its copy number increasing the host's genome size and contribute to a broad range of transcriptomic novelty on which natural selection can then act.

## INTRODUCTION

Transposable elements (TEs) can play key innovating roles in their host genomes. Indeed, they have contributed to the evolution of the immune system and of the placenta in mammals (1). In crop plants, especially in rice, they have been shown to contribute to genetic diversity on which specific traits may have been selected for (2,3). TEs can contribute to genome dynamics through their ability to rearrange genes but also by modifying how genes respond to their environment (4–6). From this perspective, retrotransposons are of particular interest: some of these elements can be activated via environmental stresses that can lead to their transcription, cDNA synthesis and integration of a novel copy in the host genome (7–9). This mode of action can be considered as a copy/paste-based mechanism. The long terminal repeats (LTRs) located at the very beginning and the end of a retroelement are essential for the life cycle of the TE and can also contain stress-response elements (10). These LTRs are interesting as the 5′ LTR provides a promoter for the TE itself and the 3′ LTR can drive transcription of host genes located downstream of the TE insertion site (11). A seminal example for such a TE has been described in blood oranges: a cold-stress responsive TE was found to be integrated in front of the *Ruby* gene in the Tarocco variety. This TE, and thus its LTRs, respond to cold stress rendering the down-stream *Ruby* gene cold-stress responsive in these oranges (12). This elegant work demonstrates how TEs can create novel links between the environment and the genome.

Here, we wanted to systematically investigate how novel insertions of *ONSEN* (*ATCOPIA78*), a heat-stress responsive retrotransposon (13), can lead to transcriptional and stress-response novelty in Arabidopsis. First, we find that *ONSEN* has a noted insertion preference for regions enriched for the H2A.Z histone variant and the H3K27me3 histone mark. This leads to a preferential insertion of *ONSEN* into genes. Looking at novel *ONSEN* insertions, we find that those can have highly diverse transcriptional consequences. This shows that mobilization of a single TE-family can lead to wide-ranging transcriptional novelty on which selection can then act.

## MATERIALS AND METHODS

### Transposable element induction and mobilome sequencing

To activate *ONSEN* in order to generate new insertions, we followed the protocol described by (14). Briefly, 20 Arabidopsis seeds (Col-0, obtained from the Paszkowski lab) were sterilized for 10 min in 10% bleach, rinsed, and sown on 9 cm Petri dishes containing 0.5× Murashige & Skoog media (Duschefa cat# M0222) with 1% sucrose, 0.5% Phytagel (Sigma cat# P8169), pH 5.8, and supplemented with two drugs: 5 μM α-amanitin (Sigma cat# A2263) and 40 μM zebularine (Sigma cat# Z4775). The seeds were left to stratify at 4°C for 48 h in the dark before being transferred to a Sanyo MLR-350 growth chamber for seven days using long days conditions (16 h of light at 24°C followed by 8 h of darkness at 21°C). After a week, the plantlets were put at 6°C for 24 h (lighting condition unchanged). This step increases the survival rate after the heat stress. The plants were then submitted to 24 h of heat stress at 37°C (again, lighting conditions unchanged). Control plants: HS control (without drug treatment), AZ control (without heat stress, but exposed to 6°C for 24 h before being returned to normal growth conditions) and wild type control (wild type without any treatment) were also produced.

We sacrificed two Petri dishes for each condition (drug and stress, drug only, stress only) to investigate the abundance of *ONSEN* in extrachromosomal circular DNA (eccDNA), as it was previously documented (14) that the combination of heat stress, α-amanitin and zebularine exposure to Arabidopsis leads to a drastic increase of *ONSEN* in a circularized form. All twenty plants from each petri dish were pooled separately and DNA was extracted using the CTAB method (dx.doi.org/10.17504/protocols.io.quidwue). Following the mobilome-seq method described by (15), for all samples, we digested linear DNA from 2 μg of total DNA for 17 h at 37°C using 10 U of PlasmidSafe (LubioScience cat# E3101K), followed by enzyme denaturation (30 min at 70°C). Digested DNA was precipitated with isopropanol supplemented with 1 μg of GlycoBlue coprecipitant (Fisher Scientific cat# 10391565). Circular DNA was then amplified through rolling circle amplification (RCA) with the Illustra TempliPhi kit (GE Healthcare cat# *2*5-6400-10), following the manufacturer's recommendation and leaving the reaction for 64 h at 30°C. DNA was once again precipitated with isopropanol and sent for Illumina paired end 150 bp sequencing at BGI, Hong Kong.

Plants not used for the mobilome-seq were transferred to soil (one plant per pot) and grown under the same long day conditions in a Sanyo MLR-350 growth chamber until seeds could be collected.

### Screening for Arabidopsis lines with new *ONSEN* insertions

F1 seeds were sown on soil and left to stratify at 4°C for 48 h in the dark before being transferred to a growth chamber under long days conditions (16 h of light at 24°C followed by 8 h of darkness at 21°C). We collected one leaf per adult plant and DNA was extracted using the Qiagen DNeasy Plant kit. The estimated number of *ONSEN* copies was measured through quantitative PCR on a Roche LightCycler 480 using TaqMan assay kit (Life Technologies cat# 04707494001) and probes specific to *ONSEN* sequence. The *ACTIN2* gene (*AT3G18780*) was used to normalize DNA levels. Primer sequences are available in the Supplementary File S1. Plants for which we detected an increase in *ONSEN* copy number through qPCR, as well as some control plants without changes in copy number, were self-fertilized for three generations for new *ONSEN* insertions to segregate and for plants to reach higher homozygosity. In F4, we selected nine lines with various number of new *ONSEN* insertions (our estimations from F1 qPCR ranged from 3 to 99 new insertions), as well as two control lines (either without drug treatment or without heat stress in the progenitors) for which we did not detect changes in the number of genomic *ONSEN* copies. We collected one adult leaf for each of these 11 lines and extracted DNA with the Qiagen DNeasy Plant kit. For two lines with the highest numbers of novel *ONSEN* insertions (line 45 and line 33), we pooled 4 siblings for the DNA sequencing. As we suspected that these two lines were still segregating, pooling allowed us to have a better idea of all the possible insertions that occurred after the transposable element activation by drugs and heat stress. Again, we quantified the total *ONSEN* copy number by qPCR in these eleven selected lines (F4 generation).

### Identification of novel *ONSEN* insertions

To identify the genomic position of the new *ONSEN* insertions, the extracted DNA of the 11 selected lines (nine lines with new insertions and two control lines) was sent to BGI, Hong-Kong for Illumina paired-end 150 bp sequencing, aiming for a minimum of 20× sequencing coverage. Quality control of the raw reads was done using FastQC (Andrews S. (2010). FastQC: a quality control tool for high throughput sequence data. Available online at: http://www.bioinformatics.babraham.ac.uk/projects/fastqc) and trimming/clipping was done using Trimmomatic (16) with parameters ILLUMINACLIP: TruSeq3:2:30:10 LEADING:20 TRAILING:20 SLIDINGWINDOW:4:20 and MINLEN:36. Quality of the reads was deemed excellent and no further actions were taken.

To precisely detect the location of novel *ONSEN* insertions, we used Transposable Insertion Finder – TIF v1.6 (17), providing the tool with the TAIR10 Arabidopsis reference genome (18) as well as the head and tail sequence of the LTR of ONSEN (TGTTGAAAGTTAAACTTGAT and AAAAGAATTTTACTCTAACA, respectively).

### Zygosity of the insertions

TIF provides genomic coordinates of novel insertion sites, target site duplication (TSD) sequences as well as the orientation of the insertion (+/−), but unfortunately not the zygosity of the insertions. As we did not find any satisfying bioinformatic tool to assess it, we developed our own method. To do so, we aligned the genomic paired-end Illumina reads on the reference genome TAIR10 (18) using Bowtie2 v2.4.2 (19) with the following parameters: --very-sensitive -X 700 -I 150. Alignment files were then filtered using Samtools view v1.12 (20) and flags -bh -f 2 -F 256 -q 28. This filtering allowed to keep only primary alignments and reads for which both mates were properly aligned (pointing inward, with an insert size from 150 to 700 bp long, and not PCR or optical duplicates) with a decent mapping score (MAPQ > 28). Once this was done, we counted the number of reads and properly mapped paired reads spanning over the insertion position. We considered the insertion to be homozygous if the minimal coverage in a 2000 bp window around the insertion position was at least 10× and not a single read or properly mapped pair of reads was spanning over the insertion point. For the two pooled samples (hcLine 33 and hcLine 45), we applied the same criteria, meaning that the insertions would be called homozygous only if all the samples of the pools were homozygous (all other cases were treated as heterozygous).

### Characterization of the insertion sites

To better understand the insertion site preferences of *ONSEN*, we first used Bedtools intersect v2.29.2 (21) with our list of novel insertion coordinates and some reference files: the Araport11 genome annotation (22), the chromatin states annotation from (23) and ChIP-Seq data on Arabidopsis Col-0 for the histone modifications H3K27me3 (24) and H2A.Z (25). To find if some conserved sequence motifs were present at the insertion site, we used WebLogo v3.7.4 (26) with the TSD sequences that were identified by TIF.

We also looked at the list of genes impacted by new *ONSEN* insertions and looked if they were enriched in specific functions. To do so, we performed a gene ontology overrepresentation test using the online tool g:Profiler (https://biit.cs.ut.ee/gprofiler/gost (27)).

### SNP, indels and CNV discovery

In addition to the detection of new *ONSEN* insertions, we wanted to detect and quantify to which extent our TE mobilization method also induced changes in DNA sequence. To identify single nucleotide polymorphism (SNP) and short insertions/deletions (indels), we used an implementation of the GATK4 pipeline (28) available here: https://github.com/snakemake-workflows/dna-seq-gatk-variant-calling. Briefly, the pipeline performs genomic alignment, duplicate removal, SNP identification, filtering and annotation. We simply fed the pipeline with the Illumina paired-end genomic reads of our eleven lines after the previously mentioned FastQC and Trimmomanic steps. Private SNP (*i.e*. present in only a single line) were additionally filtered with BCFtools (29) using read depth between 10 and 60× (FMT/DP ≥ 10 & FMT/DP ≤ 60) minimum mapping quality of 30 (INF/MQ ≥ 30) minimum genotype quality of 40 (FMT/GQ ≥ 40) and minimum number of reads supporting the alternative allele of 5 (FMT/AD[0:1] ≥ 5).

For the detection of copy number variations (CNV), we used the Hecaton pipeline (https://git.wur.nl/bioinformatics/hecaton) (30), specifically designed to identify CNV in plants, using default parameters.

We did not adjust the detection parameters for the two pooled lines (hcline33 and hcline45). We are aware that this is likely to reduce the detection power for these two samples, however we were only interested in detecting variants shared by the siblings of these pools.

All variants (SNPs, indels and CNVs) were annotated using snpEff v5.0 (31) with the latest Arabidopsis database (as of 1 June 2021).

### Heat stress trial for RNA-seq

Sequenced F4 seeds were sterilized for 10 minutes in 10% bleach, rinsed, and stratified at 4°C for 4 days in the dark before being sown on 0.5× Murashige & Skoog media (Du*s*chefa cat# M0222) and transferred to growth chambers under long day conditions (16 h of light at 24°C followed by 8 h of darkness at 21°C; 20 seeds per plate, six replicate plates). Ten days after sowing, plants were subjected to 6°C for 24 h and control plants were returned to normal long day growing conditions whereas heat stressed plants were placed at 37°C for 24 h before harvesting (three replicate plates per condition).

### RNA extraction and sequencing

Immediately following treatment, seedlings were flash frozen in liquid nitrogen and harvested in pools of five plants for transcriptome sequencing. RNA extractions were performed for three biological replicate samples for each line in each condition (*n* = 96) using the Macherey-Nagel NucleoSpin RNA kit (cat# 740955.50). Samples were sent to Novogene for Illumina 150 bp paired-end sequencing using a stranded poly-A library.

### Automated phenotyping

Seeds of the hcLines and control lines were sent to a phenotyping platform at Boyce Thompson Institute, Ithaca, NY. The seeds were stratified at 4°C for 24 h and germinated from 1 January 2021 on Cornell Mix soil. The plants were grown in Boyce Thompson Institute's walk-in growth chamber #03, with the 16 h light period (lights on from 7:00 am to 11:00 pm), 22°C throughout the growth period and 60% relative humidity. Twenty days after germination, trays were moved at 9:00 am into a growth chamber for heat treatment. In this growth chamber, the temperature was gradually increased from 22°C to 40°C over 2 h and kept at 40°C for 6 h. The trays were then moved back to the control condition growth chamber where they did recover from the heat stress under the same conditions as they did germinate in. Images were recorded using Raspberry Pi cameras and processed using PlantCV pipeline (32). The resulting data was processed in R, as described by the data analysis pipeline available at https://rpubs.com/mjulkowska/heat_experiment_TEv2.

### Differential gene expression analysis

To be consistent with what we did with whole genome sequencing data, quality control of the raw reads was done using FastQC (Andrews S. (2010). FastQC: a quality control tool for high throughput sequence data. Available online at: http://www.bioinformatics.babraham.ac.uk/projects/fastqc) and trimming/clipping was done using Trimmomatic (16) with parameters ILLUMINACLIP: TruSeq3:2:30:10 LEADING:20 TRAILING:20 SLIDINGWINDOW:4:20 and MINLEN:36. Quality of the reads was deemed excellent and no further actions were taken.

Salmon v1.4.0 (33) was used to create a decoy-aware index based on the *Arabidopsis thaliana* Reference Transcript Dataset 2 (AtRTD2 (34)). Illumina reads were quantified using Salmon quant with options -l ISF and --validateMappings. This allowed us to get a read count for both strands. As we were also curious to see if *ONSEN* insertions would affect only sense or antisense transcription, we performed the previous step two more times, with -l ISR --validateMappings --incompatPrior 0.0 to quantify only antisense transcripts and -l ISF --validateMappings --incompatPrior 0.0 to quantify only sense transcripts. We added one read count to all count values produced by salmon to avoid division by 0. We used DESeq2 (35) through the European Galaxy platform (usegalaxy.eu, (36)) with default parameters to detect differentially expressed genes (DEG). Our three sets of quantification files from Salmon (sense transcripts only, antisense transcripts only, and both) were processed separately.

We used g:Profiler (27) to search for gene ontology (GO) over representation in the list of DEG obtained for each line in DESeq2. We treated for each condition (control and heat stress) and each line, we looked for enriched GOs in the list of upregulated genes, downregulated genes, and both up- and downregulated genes together.

In order to detect transcripts containing pieces of *ONSEN* sequences, we also reconstructed a *denovo* transcript assemblies from the RNA-seq data using rnaSPAdes (37) with the --pe1-fr option.

### Evaluation of individual *ONSEN* copy activity

The Arabidopsis genome (Col-0 accession) contains eight full length *ONSEN* copies (*AT1G11265*, *AT1G21945*, *AT1G48710*, *AT1G58140*, *AT3G32415*, *AT3G59720*, *AT3G61330*, *AT5G13205*), and we wanted to see if some specific copies would be more active than others in the genome, mobilome and transcriptome. To investigate this, we first aligned the nucleotide sequences of all eight copies using MEGA X (38) to identify all SNP and indels, as well as to generate a ‘consensus’ *ONSEN* sequence (Supplementary File S2) and identify all non-LTR SNPs unique to each of the eight copies (Supplementary File S3). For each Illumina dataset (mobilome, genome, transcriptome), we aligned the reads on this *ONSEN* consensus sequence using Bowtie2 v2.4.2 (19) with the following parameters: –very-sensitive -X 700 -I 150. Alignment files were then filtered using Samtools view v1.12 (20) and flags -bh -f 2 -F 256. This filtering allowed to keep only primary alignments and reads for which both mates were properly aligned (pointing inward, with an insert size from 150 to 700 bp long, and not PCR or optical duplicates). We then, for each SNP unique to a given *ONSEN* genomic copy, calculated the allele frequency (number of reads containing the copy specific alternative nucleotide divided by the total number of reads covering that exact position). For mobilome and transcriptome data, we calculated the average of the nucleotide frequencies for all the SNPs exclusive to each *ONSEN* copy to get an estimate of its relative abundance. The genomic data was processed slightly differently: As we had data for two control lines without novel *ONSEN* insertions, we could calculate, when aligning the Illumina reads on the *ONSEN* consensus sequence, the actual real measured allele frequency for each SNP specific to each of the eight full length genomic copies. In theory, all unique SNP should account for 1/8 of the reads when aligned, but it was not necessarily the case. We used this information to weight each SNP so they account for this theoretical value and have a better estimation of the identity of each novel *ONSEN* insertion.

### Results integration in genome browser

To be able to explore the produced results in a more visual way, we produced several genome browser tracks that we integrated in our local instance of JBrowse (39) available at the following url (https://jbrowse.agroscope.info/jbrowse/?data=tair10). RNA-seq data was mapped to the genome using STAR (40) with the following attributes: --outSAMtype BAM SortedByCoordinate --twopassMode None --quantMode - --outSAMattrIHstart 1 --outSAMattributes NH HI AS nM ch --outSAMprimaryFlag OneBestScore --outSAMmapqUnique 60 --outSAMunmapped Within --outBAMsortingThreadN 16 --outBAMsortingBinsN 50.

## RESULTS

### Phenotypic changes resulting from novel *ONSEN* insertions

In order to mobilize the endogenous *ONSEN* TE in wild type Arabidopsis plants, we have used α-amanitin and zebularine (AZ) in combination with heat stress, as previously described (14). The selfed progeny (in F1 and F4) of the treated plants were then screened for increased *ONSEN* copy numbers by qPCR. Using this approach, we generated a collection of 9 high-copy lines (hcLines) that, according to qPCR, contained novel *ONSEN* insertions. We also generated two control lines that were the progeny of plants treated either with heat stress and without drugs (HS control) or without heat stress but only with the drugs (AZ control). Detailed qPCR quantifications in F1 and F4, as well as the correlation with the quantification performed by Illumina sequencing (mentioned below) are available in Supplementary File S4.

**Figure 1.**
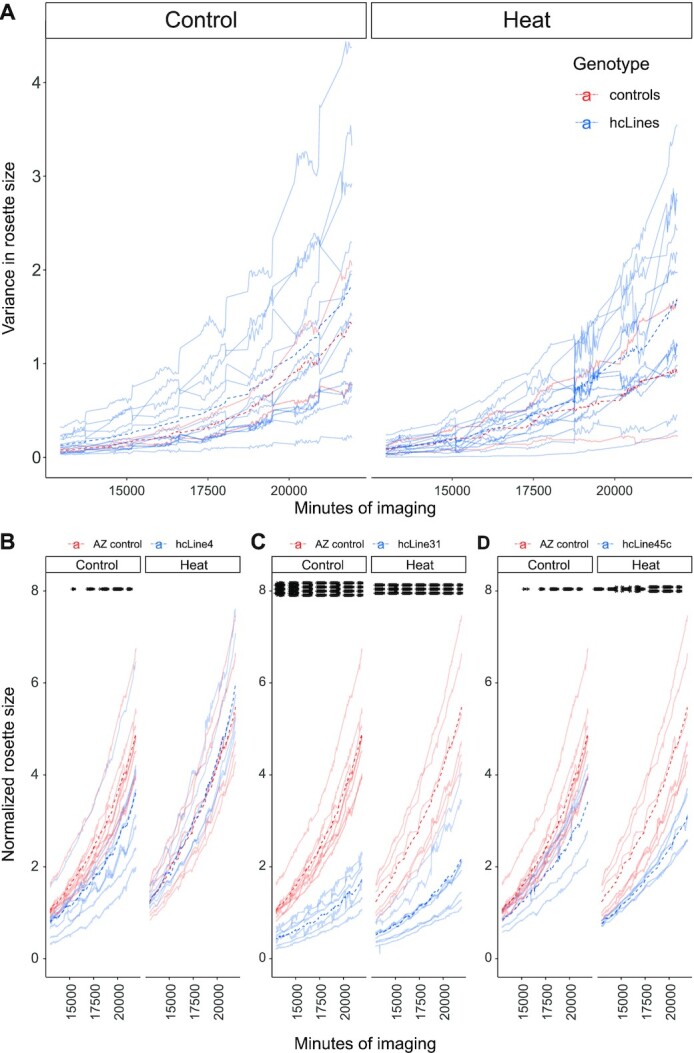
Rosette leaf area over time measured by continuous imaging. Images were recorded using Raspberry Pi cameras and processed using the PlantCV pipeline. The x axis represents the time of imaging (in minutes) starting at 20 days post-germination (corresponding to 12500 minutes of imaging) and the y axis the normalized rosette area. The rosette area has been normalized for the average plant area per Arabidopsis tray at the time point prior to exposure to heat stress (20 days after germination 8:00 am), to accommodate the spatial variation between the rigs. (**A**) Average (six replicates per line) rosette size growth over time under non-stress (Control) and heat stress (40°C for 6 h) conditions for both AZ and HS control lines (red) as well as the 9 hcLines (blue). Increase in rosette size for all replicates (transparent lines) and the genotype average (dashed line) for (**B**) hcLine4, (**C**) hcLine31 and (**D**) hcLine45c compared to AZ control. The area of the rosettes was monitored every 30 min during the 16 h light period for 7 consecutive days. The significant differences between AZ control (red) and individual hcLines (blue) were tested using one-way analysis of variance (ANOVA) and the asterisks indicate the *P*-value <0.05 for each timepoint.

First, we assessed phenotypic changes in the hcLines (F4 generation) using an automated phenotyping system based on Raspberry Pi computers. We monitored the increase in rosette area of each of the lines over a period of one week, starting 12 days after germination. Overall, we observed that the hcLines showed a greater variability between individual genotypes in rosette size compared to control lines, (Figure 1A) and most developed smaller rosettes compared to both control lines (HS control and AZ control) under non-stress growth conditions (Figure 1A). Since *ONSEN* responds to heat stress, we wanted to address if novel *ONSEN* insertions could modify how hcLines respond to heat stress. For that, we grew the plants under non-stress conditions for 20 days and then subjected them to intensive heat stress (6 h at 40°C). After returning the plants to non-stress conditions, we monitored rosette growth for each individual line using six biological replicates for each genotype. Most hcLines performed worse than the control lines (Figures 1A–D). A notable exception was hcLine4 which grew smaller rosettes under non-stress conditions, but no significant differences could be observed between hcLine4 and AZ control (Figure 1B) or the HS control after heat stress treatment (Supplementary Figure S1). All the raw data for the phenotyping is available here: https://cornell.app.box.com/s/t7n63w5cxn6psim139x6lkfxkmydlg7k. hcLine31 and hcLine45c are both shown as examples of lines with strong and intermediate rosette growth reductions compared to control lines under both non-stress and heat stress conditions (Figure 1C and D). These results suggest that mobilization of *ONSEN* results in reduced rosette growth and, in general, increased heat stress sensitivity, except for hcLine4, where heat-stress resulted in a wild-type like growth behavior (Figure 1B).

### Genomic impact of epigenetic drug treatments

In order to identify the changes in *ONSEN* copy numbers and the precise novel insertion sites we used short-reads based whole genome sequencing (WGS) data of two control lines (HS and AZ control) and nine hcLines. Importantly, it has been documented that treatments with DNA methylation inhibiting drugs such as zebularine can lead to DNA damage (41). Indeed, chromosome breakages have been documented in several plant species (41,42) and changes in telomere length have also been observed (43). We therefore first wanted to assess the magnitude of DNA damage caused by the drug treatments used to mobilize *ONSEN*. We quantified mutation rates, by counting private mutations that were unique to the progeny of each treated line. Using this approach, we observed a trend that AZ treatment increased the overall mutation rates: 165 private single nucleotide polymorphisms (SNPs, Table 1) were detected in the progeny of the HS control plant, 185 SNPs in the progeny of the AZ treated control line, and an average of 194 mutations in the hcLines (min 158, max 272, σ 35.9). Using a single sample Student's *t*-test, we determined that the mutation rate of the hclines was slightly higher than the HS control (*P*-value = 0.04226), but not different from the AZ control (*P*-value = 0.1532), a trend hinting that exposure to α-amanitin and zebularine could increase the overall mutation rate. However, as only one control of each kind was sequenced, this significance should be taken with caution. We also detected private small insertions and deletions (indels, Table 1) independently from the SNPs. In this case, the single sample Student test showed no significant differences in the amounts of indels between the hcLines (mean 142, min 100, max 246, σ 43.2) and neither the HS control (143 indels, *P*-value = 0.6562) nor the AZ control (135 indels, *P*-value = 0.9286).

**Table 1.** Private genomic variations (SNPs, Indels, CNVs) and organelle read coverage in hclines

hcLine	Private SNPs	Private Indels	Private CNVs	Mt coverage	Pt coverage
2	171	121	120	1.78×	1.46×
4	169	144	201	2.01×	1.46×
7	158	115	88	1.49×	1.15×
9	160	100	117	1.92×	1.46×
18	272	246	179	1.56×	1.39×
31	195	152	138	1.76×	1.63×
33	210	122	126	2.49×	1.49×
34	213	119	148	1.61×	1.67×
45	197	156	106	2.28×	1.16×
HS control	165	143	64	1×	1×
AZ control	185	135	136	1.9×	1.6×

Mitochondria (Mt) and Chloroplast (Pt) Illumina read coverage were measured in comparison to HS control (1×).

In order to better understand if the drug treatment or the TE mobilization could affect copy number variations (CNV, Table 1), we used a specialized pipeline to detect them in Arabidopsis (30). Focusing again on private variations (excluding *ONSEN* copy number changes), the pipeline identified the lowest number of CNVs in the HS control line, with only 66 being identified. 136 were found in the AZ control, and an average of 129 in the hcLines (min 88, max 201, σ 35.6). There is a significant difference in the number of CNVs observed between the HS control (single sample Student's *t*-test, *P*-value = 0.01113) and the hcLines, but not between the AZ control and the hcLines (*P*-value = 0.5343). This hints that the drug combination may lead to an increase of the CNV count, but as previously mentioned, this significance should be taken with caution as only a single control of each kind was sequenced.

Notably, in hcLine9 which carries three novel *ONSEN* insertions, we detected a 1.57 mb duplication on chromosome 4 around positions 11,953,199 to 13,524,050. By looking at read mapping patterns at the borders, we concluded that it was the result of a large tandem duplication. No other major structural changes were found in the other lines. Interestingly, we also noticed a significant increase in read coverage for the mitochondria and chloroplast genomes when compared to HS control (single sample Student's *t*-test, *P*-value s of 5.045e^−05^ and 9.708e^−05^, respectively). The AZ control line had a 1.9- and 1.6-fold increase in read coverage for mitochondria and chloroplasts, and in average, hcLines had a 1.88 (min 1.6, max 2.5, σ .34) and 1.44 (min 1.2, max 1.7, σ 0.2) fold increase, respectively. This suggests that epigenetic drugs can either affect overall plastid content and/or plastid genome copy numbers, or that heat stress without drugs could lead to the loss of organelles.

Using TIF, we looked for novel insertions of other transposable elements, based on the annotation provided by the Unité de Recherche Génomique Info (URGI, https://urgi.versailles.inra.fr/Data/Transposable-elements/Arabidopsis). Besides novel *ONSEN* insertions, we did not detect new insertions of other known TEs in the hcLines. VCF files including all private SNPs, indels and CNV, as well as annotations for these variants obtained by snpEff, are available in Supplementary File S5.

### *ONSEN* preferentially integrates into coding exons enriched for the H3K27me3 histone mark and H2A.Z histone variant

Next, we identified the exact *ONSEN* copy numbers and insertion sites using the aforementioned WGS data. Overall, we detected 237 novel *ONSEN* insertions in the 9 hcLines. Notably, no novel insertions were detected in the two control lines. Zygosity analysis revealed that 101 insertions were heterozygous and 136 were homozygous. The number of detected insertions here was slightly superior compared our qPCR estimations (both in F1 and F4), but correlated well (*r*^2^ = 0.9682 in F1, *r*^2^ = 0.9999 in F4, Supplementary File S4). To capture as many insertion events as possible, genome sequencing for hcLine33 and hcLine45 was performed using two pools of 4 siblings. For these lines we called zygosity for the pool and not for individual plants. A summary of these insertions can be found on Table 2, and more detailed characterization is available in Supplementary File S6.

**Table 2.** WGS based detection of novel *ONSEN* insertions in the hcLines and controls

	# Novel *ONSEN* insertions		
hcLine	Homozygous	Heterozygous	Note
2	7	1	
4	23	3	
7	26	7	
9	1	2	1.57 mb tandem duplication on chr4
18	11	2	
31	6	4	
33	46	53	Pooled DNA from four sibling plants
34	6	1	
45	10	28	Pooled DNA from four sibling plants
HS control	0	0	Control line exposed to heat stress only
AZ control	0	0	Control line exposed to drugs (α-amanitin & zebularine) only

**Figure 2.**
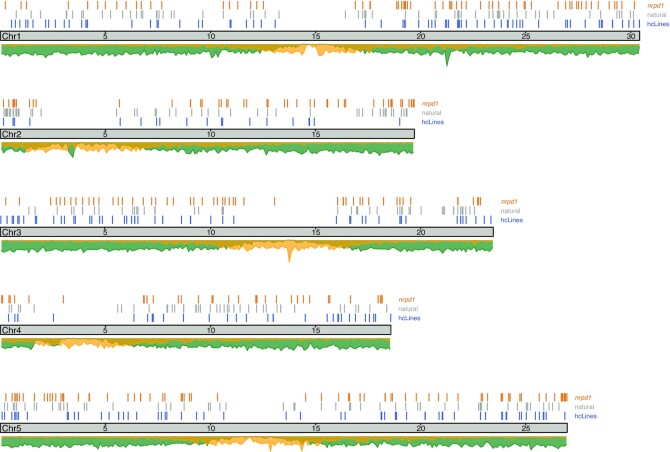
Genome-wide distribution of novel *ONSEN* insertions in the Arabidopsis genome. Novel insertions detected in this study are represented in blue (hcLines) and those previously reported (44,78) for natural populations and *nrpd1* plants in grey and orange, respectively. The density plots below the grey chromosome schemes show gene density (green) and TE density (yellow). Units are given in Mb.

The 237 novel insertion loci are seemingly evenly spread over all five chromosomes yet avoiding TE-rich pericentromeric regions (Figure 2). We compared our set of novel *ONSEN* insertions with those previously described in wild plants (natural insertions, Figure 2) and in NRPD1 defective plants (*nrpd1* insertions, Figure 2) (44,78). We observed that the distribution of *ONSEN* insertions was similar between our hcLines and the previously described natural and *nrpd1 ONSEN* insertions.

**Figure 3.**
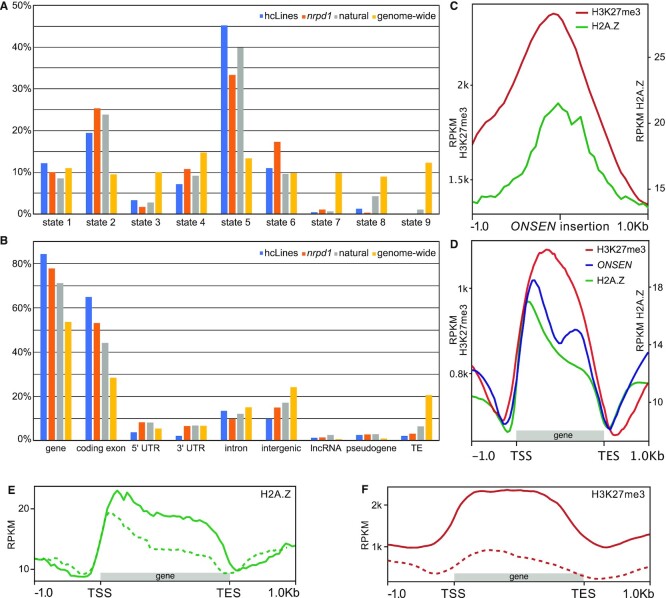
Genomic and epigenetic features at *ONSEN* insertion sites. (**A**) Frequencies of chromatin states, as defined by (23), found at *ONSEN* insertion sites and genome wide abundance of each of these respective states (in yellow). (**B**) Frequencies of genomic features from the Araport11 annotation (22) found at *ONSEN* insertion sites, as well as the genome-wide proportions of these features (UTR = untranslated regions, lncRNA = long non-coding RNA, TE = transposable elements). (**C**) Abundance of the H3K27me3 (24) histone mark (in red) and H2A.Z (25) (in green) one kb upstream and downstream of the 237 novel *ONSEN* insertions. (**D**) Global abundance of H3K27me3 (in red) and H2A.Z (in green) on 211 genomic features (genes, pseudogenes, transposable elements, long non-coding RNA) with novel *ONSEN* insertions. The distribution of *ONSEN* insertions is displayed in blue. (**E**) Abundance of H2A.Z on 211 genomic features (genes, pseudogenes, transposable elements, long non-coding RNA) with novel *ONSEN* insertions (full line) and on 215 randomly sampled genes (dotted line). (**F**) Abundance of H3K27me3 on 211 genomic features (genes, pseudogenes, transposable elements, long non-coding RNA) with novel *ONSEN* insertions (full line) and on 211 randomly sampled genes (dotted line). All density plots were made using a bin size of 50 bp.

We then looked at the annotation at the insertion sites, by intersecting them with the Araport11 genomic annotation (22) and the chromatin states as identified by (23). While genome wide, all the nine chromatin states cover similar proportions of the genome (from 9.02% to 14.79% of the genome), *ONSEN* insertion loci do not follow the same distribution. We observed that *ONSEN* had a strong insertion site preference for coding exons of genes (65% of the loci) and chromatin state 5 (45% of the loci, while this state covers 13.40% of the genome), which is enriched in H3K27me3, H2A.Z and the H3.1 histone variant (Figure 3A). Overall, less than 10% of the insertion loci were found outside of annotated genes. In total, 84.39% of the insertions are in genic features, while these features cover 55% of Arabidopsis genome, showing a distinct preference for genes (Figure 3B). Chromatin states 2, 1 and 6 also account for a significant proportion of the loci (19%, 12% and 11%, respectively). Notably, these three chromatin states are all characteristically enriched in H2A.Z and H3K27me3. This was further confirmed when we plotted H2A.Z (25) and H3K27me3 (24) densities at *ONSEN* insertion sites (Figure 3C). When we plotted the *ONSEN* insertion site density over genes, its pattern largely correlated with that of H2A.Z (Figure 3D). We then tested if genes targeted by *ONSEN* tend to have higher levels of H2A.Z histone variant and H3K27me3 marks. Indeed, genes targeted by *ONSEN* are enriched with these two chromatin marks (Figure 3E, F). *ONSEN* is clearly avoiding insertion in chromatin states 7, 8 and 9 (0.42%, 1.27% and 0.00%, respectively), which represent intergenic and heterochromatic regions of the genome. These three states cover from 9 to 12% of the genome.

Since *ONSEN* was often targeting genes, we wanted to test if *ONSEN* showed a preferential insertion orientation with respect to the gene. When inserted in an annotated genomic feature (211 loci out of 237; gene, pseudogene, transposable element or long non-coding RNA), we found no preference for the orientation of the insertion, which in 104 cases was in the same orientation as the annotated feature, and 110 cases in the opposite orientation of the feature. We also observed a seemingly bimodal distribution of *ONSEN* insertion positions within gene bodies, with one peak located between 15% and 25% (48 insertions) of the gene length and a second one located between 60% and 75% (38 insertions) of the gene length (Figure 3D).

We performed a gene ontology (GO) overrepresentation test using g:Profiler and 202 annotated gene IDs with novel *ONSEN* insertions (we excluded pseudogenes and transposable element genes) to determine if *ONSEN* targeted genes with specific functions. We noticed a mild, yet significant, enrichment in genes with molecular functions associated with NAD+ (GO:0003953, GO:0050135, GO:0061809), ADP binding (GO:0043531), as well with pathways documented as phosphatidylinositol signaling system (KEGG:04070) and inositol phosphate metabolism (GO:0035299, GO:0052746, KEGG:00562). Detailed GO results are available in Supplementary File S6.

Through sequence logo analyses, we investigated if a common sequence could be found for the target site duplications (TSDs) at the insertion sites. Unlike what was previously reported for *ONSEN* insertions in natural populations (44) we could not identify common nucleotide pattern between the TSD sequences (Supplementary Figure S2).

In order to identify potential *ONSEN* insertion hotspots, we used a 10 kb sliding window to detect multiple insertions within a small genomic distance. We used our novel hcLines insertions (*n* = 237) as well as the previously identified *nrpd1* (*n* = 281) and natural (*n* = 279) insertions (44,78). We identified 79 windows with two insertions, 16 with three insertions, 2 with four insertions, 2 with five insertions, 1 with six insertions and another one with seven insertions (Supplementary File S7). In three cases, two insertions were located less than 12 bp from each other. We calculated that the distribution of the insertions and hotspots over genome-wide 10 kb windows could not be explained by a classical random model (khi^2^, *P*-value = 0). This suggests that it is highly unlikely that the number of hotspots we observed only occurred randomly. Using g:Profiler, we searched for GO overrepresentation in genes with multiple insertions, but we could not find anything significant. We also compared the density of genes, long non-coding RNAs (lncRNAs) and TEs in these hotspots to the one displayed by 100 randomly sampled genomic windows of the same length. We observed no differences suggesting that these potential *ONSEN* hotspots are representative for the ‘normal’ genomic landscape of Arabidopsis.

### Four genomic *ONSEN* copies are responsible for the new insertions

**Figure 4.**
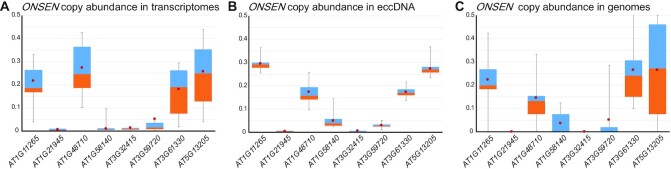
Activity of individual *ONSEN* copies. (**A**) Relative abundance of the 8 *ONSEN* copies in hcLines transcriptomes (**B**) in extrachromosomal circular DNA (eccDNA) and (**C**) in the integrated copies present in the hcLines. First and last quartiles are represented by the top and bottom whiskers. Blue boxes represent the second quartile and orange boxes the third quartile. Average is displayed by the red dot in the boxes.

The Col-0 Arabidopsis accession has 8 ‘full length’ *ONSEN* copies and we wanted to identify if one or more copies were the origin for new insertions. Based on the Col-0 reference data, we identified SNPs unique to each *ONSEN* copy, and through allele frequencies of these SNPs in transcriptome, extrachromosomal circular DNA (eccDNA) and genome data, we estimated the proportion of each copy in different datasets (Figure 4) following this TE’s life cycle. We observed that four copies (*AT1G11265*, *AT1G4870*, *AT3G61330* and *AT5G13205*) represent over 90% of the abundance in these datasets. While all these four copies are in relative equal average abundance in the RNA-seq data (although there is a lot of variability between the samples, Figure 4A), *AT1G11265* and *AT5G13205* have a higher abundance in eccDNA (Figure 4B). In terms of genomic integration, *AT1G4870* produces on average less new copies than the three other main copies (Figure 4C). Two copies, *AT1G21945* and *AT3G3241*, are very weak throughout the lifecycle steps we quantified here (transcriptome, genome and eccDNA) and we could not find any evidence that they contributed to a single novel *ONSEN* insertion.

### Transcriptomic impact of epigenetic drug treatments

Next, we wanted to assess how epigenetic drug treatments and novel *ONSEN* insertions impacted the transcriptome. To mobilize *ONSEN*, we used drugs (zebularine and α-amanitin), that both interfere with DNA methylation (45), in combination with heat stress (14). It has been documented that treatment of plants with such epigenetic drugs can lead to heritable changes in phenotype and DNA methylation patterns (46). These phenotypic changes can be caused by the acquisition and stabilization of epialleles.

To investigate the transcriptional changes resulting from drug treatments and the mobilization of TEs, we performed RNA-seq on control plants and the hcLines (F4 generation). Notably, an important limitation to our transcriptome analysis was the great number of significant differentially expressed genes (DEGs) we identified in the hcLines under control conditions (ranging from 1072 to 6541 DEGs, Supplementary File S8). Because of the complexity of the transcriptomes of the hcLines it would hardly be possible to separate direct effects resulting from DNA methylation changes, *ONSEN* insertions, indirect secondary effects resulting from those changes, other mutations and changes in plastid content. Therefore, we solely focused our analysis on the direct transcriptional consequences that may have been caused by DNA methylation changes.

**Figure 5.**
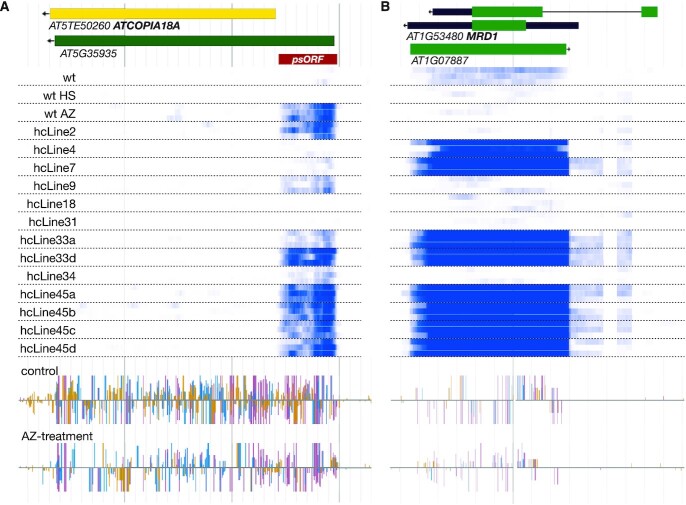
Epigenetic drug treatments result in stable transcriptional changes. The upper part represents genes (coding region in green, UTR in thick black, intron in thin black, arrows indicate the orientation of the transcription) and TE annotation (yellow). Middle part displays a heat map of transcription based on our RNA-seq data under control conditions (white = no transcription, dark blue ≥200 transcripts per million) for controls and the hcLines (three biological replicates are shown for each plant line). The lower two histograms show cytosine methylation levels in control (top) and AZ-treated (bottom) plants (color code for the DNA methylation contexts: yellow: CHH, blue: CHG and red: CG). (**A**) Constitutive transcription observed at *psORF* in the AZ control and several hcLines (**B**) Genome browser view showing overexpression of *MRD1* in multiple hcLines.

In order to assess whether the epigenetic drug treatments may have led to stable transcriptional changes, we surveyed regions of the genome previously reported to be epigenetically unstable. A notable example is a region termed ‘pseudo ORF’ (*psORF*) identified by (47) (Figure 5A). It is a region 5′ to *AT5G35935*, located just in front of an *ATCOPIA18A* element. It has been reported that NERD, a GW repeat- and PHD finger-containing protein, is involved in RNA-directed DNA methylation to transcriptionally silence this locus. We found that this locus was overexpressed in 5 of the hcLines and in the line that was only treated with epigenetic drugs (AZ control, Figure 5A). Our previously reported methylome analysis shows that plants exposed to the drug treatment showed a reduced DNA methylation at this locus, suggesting the formation of an epiallele (reduction of DNA methylation of 26%, 46% and 70% for the CG, CHG and CHH contexts, respectively, Figure 5A histograms) (14).

Two notable genes previously reported to be metastable from an epigenetic point of view and that we found to be differentially expressed are *MTO 1 RESPONDING DOWN 1* (*MRD1, AT1G53480*) (48) and *Epistatic HDA6-RdDM Target 9* (*ERT9*, *AT5G24240*) (49,50). *MRD1* is overexpressed in 7 hcLines (of which all four of the hcLine45a-d sister lines) and DNA methylation reduced by the epigenetic drug treatment (57% and 67% loss in CG and CHG methylation, respectively; no changes in CHH methylation) suggests that this locus is now fixed in a different epigenetic state as compared to the parental line (Figure 5B). *ERT9* was identified to be metastable at the DNA methylation level in a screen performed to identify spontaneously occurring variations in DNA methylation over 30 generations of inbreeding (49). Furthermore, this locus was also previously found to be strongly up regulated in plants defective in HDA6, Pol IV and Pol V (50). Here, we found that *ERT9* was highly transcribed in hcLine9, hcLine18 and the sister lines hcLine45a-d (Supplementary File S8). Also at this locus, DNA methylation was reduced due to AZ treatment (57% and 67% loss in CG and CHG methylation, respectively, no changes in CHH methylation; Supplementary Figure S3) while no genomic variant (SNP, CNV or indel, see Supplementary File S5) was detected in that region.

Three other notable metastable epigenetically controlled loci identified in our screen were the non-coding RNA *RITA* (48), and three members of the *SADHU* retroposon family (*AT3G44042*, *AT3G02515*, *AT3G42658*) (51) (Supplementary File S8). However, since these signals were only observed in single lines (the sister lines hcLine33a and hcLine33d) we cannot conclude on whether these are the result of genetic or epigenetic changes. Importantly, *AGO5* is mutated by an *ONSEN* insertion in this line, which may directly interfere with silencing of *RITA* and/or *SADHU* (48,52). Finally, we identified a Gypsy retrotransposon (*AT5G28335*) that was upregulated in all sister lines of the two independent hcLine33 and hcLine45 families.

### Transcriptomic impact of novel *ONSEN* insertions

**Figure 6.**
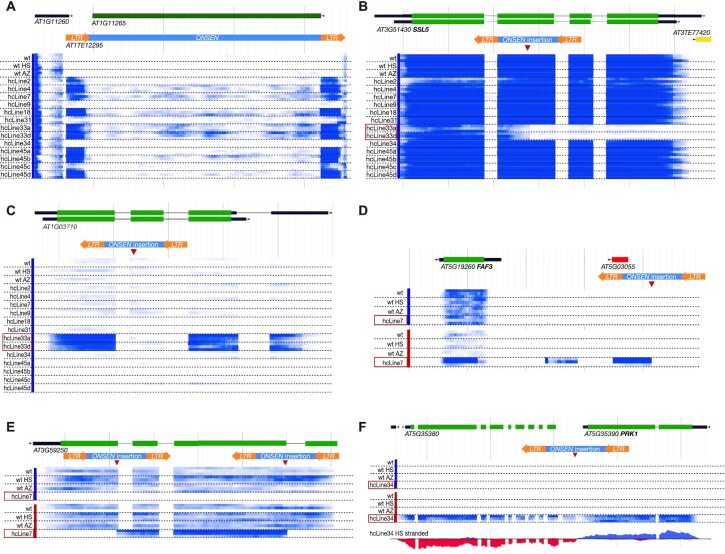
Genome browser views showing quantitative and qualitative consequences of novel *ONSEN* insertions. The heat maps in blue under the gene annotations (coding region in green, UTR in thick black, intron in thin black, TE annotation in yellow, arrows signal the orientation of the transcription) indicate RNA-seq signal intensity (white = no transcription, dark blue > = 200 transcripts per million). (**A**) Transcript levels at one of the ancestral *ONSEN* insertion sites. Increased levels of RNA-seq signal was observed at the *ONSEN* LTRs (indicated in orange) in some of the hcLines. (**B**) Example of a transcript truncation shown by the absence of RNA-seq signal following the *ONSEN* insertion (in red) in hcLine33a and hcLine33d. (**C**) constitutive overexpression and exon skipping at *AT1G03710* resulting from the *ONSEN* insertion in hcLine33a and hcLine33d. (**D**) Novel heat stress dependent spliced transcript reaching from the insertion site to *FAF3* in hcLine7. (**E**) two *ONSEN* insertions in *AT3G59250* disrupting the gene in hcLine7 yet producing a transcript upon heat stress. (**F**) Acquisition of heat stress responsiveness due to an *ONSEN* insertion between *AT5G35380* and *AT5G35390* in hcLine34. The colored bars on the left next to the sample's names indicate control conditions in blue and heat stress treated samples in red. Unnecessary tracks have been removed for better visualization. The red boxes around the names of hcLines mark the lines that contain an *ONSEN* insertion in the depicted region.

Next, we wanted to investigate how novel *ONSEN* insertions directly impact gene expression at their insertion sites, both under control and heat stress conditions (a complete list of all DEGs in all hcLines compared to a wild-type control is presented in Supplementary File S8). First, we wanted to know if additional TE insertions had an impact on *ONSEN* transcription in control conditions (without heat stress). We noted strong RNA-seq signals at the *ONSEN* LTRs suggesting that they may be transcribed in some of the hcLines even in the absence of heat stress (Figure 6A). No *ONSEN* LTR transcription was detected for in the wt control, the HS control and the AZ control. It is notable that transcription was in sense orientation and specifically confined to the LTRs. Since our stranded RNA-seq assay is designed to only detect polyadenylated transcripts we assume that RNA Pol II plays a role in the biogenesis of these transcripts. We could not find any correlation between the number of new *ONSEN* copies and the LTR-specific transcription. Confirming our previous observations (14), changes in *ONSEN* TE copy number did not lead to a significant variation in its own expression after heat stress, as we did not find *AT1G11265* (a gene annotation covering an active copy of *ONSEN*) in our DEG list.

We then systematically investigated the transcriptional effect of each individual *ONSEN* insertion. Examples as to how *ONSEN* can affect gene expression are shown in Figure 6B-F (detailed graphs showing how reads are mapping to these specific loci are provided in Supplementary Figure S4). An often-documented effect of novel TE insertions are gene knockouts. An example for that is depicted in Figure 6B where *ONSEN* is integrated in the second exon of *AT3G51430* / *SSL5* in the two sister lines hcLine33a and hcLine33d. The gene appears to be normally transcribed at the 5′ end but the RNA-seq signal abruptly stops at the *ONSEN* insertion site. Figure 6C shows an *ONSEN* insertion with two distinct effects: Overexpression of the *AT1G03710* gene in the absence of heat stress and at the same time exon skipping, as the second exon where *ONSEN* is located only produces a background level of RNA-seq signal.

Among the 237 insertions we found 6 that produced heat-stress dependent novel transcripts that we did not detect in control plants, and which are not annotated in Araport11. These transcripts are always composed of a mix of intergenic and genic regions, and two of them even display splicing (one example shown in Figure 6D). A pronounced effect was observed under heat stress: 61 genes that are not transcribed under heat stress in wild type became heat-stress responsive due to novel *ONSEN* insertions. Of these, however, only two produced genic transcripts that appeared to be intact (AT2G27880 in hcLine33a and hcLine33d; AT5G59105 in hcLine18). In many cases, transcripts were either truncated (36 cases) or in antisense orientation (19 cases). For some of the homozygous *ONSEN* insertions, the transcriptional profile of the gene carrying an *ONSEN* insertion seemed normal. However, by investigating the *de novo* transcriptome assemblies of the hcLines, we found 7 transcripts in control conditions and 32 in HS containing pieces of LTR sequences in them, hinting at a possible exonization of *ONSEN* in these transcripts. Of note is AT3G59250, which was targeted by two *ONSEN* insertions in hcLine7 (Figure 6E): Here, the two insertions entirely abolish transcription of the gene, yet upon heat stress, transcription is initiated at the 5′ *ONSEN* insertion and ends precisely at the 3′ *ONSEN* insertion while maintaining the third exon of this gene. We were also able to detect the presence of *ONSEN* LTR sequences in the transcripts of 35 genes containing *ONSEN* insertions in hcLines grown under control conditions (out of 106 transcribed genes, so 33.0%). The number of genes increased to 139 when hcLines were grown under heat stress condition (out of 195 transcribed *ONSEN*-containing genes, so 71.3%). Transcripts containing *ONSEN* LTR sequences were almost always truncated in 5′ (6 in control, 78 in heat stress condition) or in 3′ (26 in control, 51 in heat stress condition). This demonstrates that transcription can start within *ONSEN* LTRs and continue into the gene where it is inserted (5′ truncation) or start at the transcription start site of the gene and terminate at the end of the inserted *ONSEN* sequence (3′ truncation). A quantification of the most common effects is displayed in Table 3 and detailed effects for each insertion can be consulted in Supplementary File S6.

**Table 3.** Quantification of the observed effect on transcripts located at the insertion site of novel *ONSEN* copies in the hcLines under control and heat stress conditions.

Structural impact on transcripts	Control condition	Heat stress
*ONSEN* exonisation	**7**	**32**
5′ truncation	**9**	**67**
3′ truncation	**38**	**29**
Intron retention	**1**	**20**
Exon skipping	**5**	**3**
Transcript fusion with intergenic sequence	**0**	**7**
Transcript fusion with nearby gene(s)	**0**	**19**
**Impact on expression levels**		
No transcription in wt and no transcription in hcLine	**111**	**40**
Transcription in wt and normal transcript in hcLine	**40**	**26**
Intact transcripts, upregulated	**2**	**2**
Intact transcripts, downregulated	**17**	**4**
Knocked out genes	**3**	**3**
Antisense gene transcription	**7**	**48**
Novel, unannotated, transcripts	**0**	**6**

Transcription was compared to wt control. Note that the sum of the effects is more than the number of *ONSEN* insertions (237 insertions) because some insertions can have multiple effects (ex: truncation of the transcript and transcription of an antisense transcript at the same locus). Exonisation means that *ONSEN* sequence, or part of *ONSEN* sequence, is found in the mRNA-sequence of the gene where it is inserted. Fusion with other genes means that the transcript at the loci of insertion also contains sequence of the next 5′ or 3′ annotated genes. Similarly, fusion with intergenic sequence means that the transcripts at the loci of *ONSEN* insertions contains intergenic sequences which are normally not transcribed. Here, truncation means that the gene is transcribed, but the transcription abruptly stops before or after an *ONSEN* insertion (this could lead to a variety of consequences, especially protein truncation). When a gene is normally transcribed in wt or control lines, and no transcription was detected in the hcLine with the *ONSEN* insertion, we used the term ‘knocked out’.

**Figure 7.**
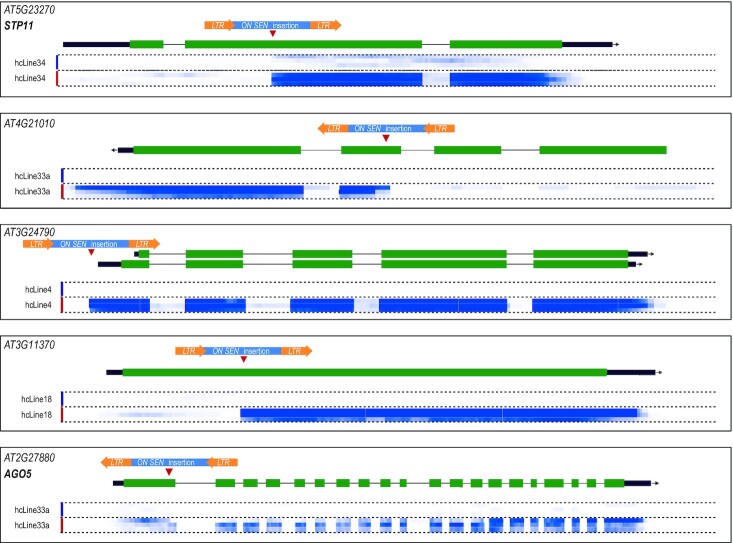
Acquisition of heat-responsiveness via novel *ONSEN* insertions. Extracts of genome browser views. The upper parts represent gene annotation (coding region in green, UTR in thick black, intron in thin black). The heat maps in blue under the gene annotations indicate RNA-seq signal intensity (white = no transcription, dark blue ≥200 transcripts per million). The colored bars on the left next to the sample's names indicate control conditions in blue and heat stress treated samples in red. Unnecessary tracks have been removed for better visualization. Marked heat-responsiveness can be observed right downstream of *ONSEN* insertions (red triangles). In the cases of *STP11*, *AT4G21010* and *AT3G11370* this leads to truncated transcripts. *AT3G24790* appears to be intact thanks to the insertion of *ONSEN* in the promoter region. *AGO5* is unique as it carries an insertion at the end of the first exon, yet this seemingly leads to a properly spliced heat-stress dependent full-length transcript.

Finally, we found that *ONSEN* can act as both, a heat-stress dependent promoter and enhancer: Its insertion right between *AT5G35380* and *AT5G35390*/*PRK1* rendered both genes heat-stress responsive (Figure 6F). This is notable as the *ONSEN* LTR points towards *PRK1* and yet the gene located 5′ upstream to the insertion site also became heat stress responsive. RNA-seq read mapping for loci presented on Figure 6B–F is available in Supplementary File S4. Five additional examples of how novel *ONSEN* insertions lead to the acquisition of heat-responsiveness in genes are shown in Figure 7.

We then wanted to investigate if there was a link between gene expression levels and *ONSEN* insertion site preference. We looked at the steady state transcript levels of genes in wild-type plants that were targeted by *ONSEN* insertions in the hcLines. We noticed that average and median gene expression for these genes (see Supplementary File S6 for the list of all genes where novel *ONSEN* insertions were found) was below the genomic median and average. We then wanted to see if these genes truly had a lower steady-state transcript level compared to the global average. To do so, we compared the transcription values (transcript per million - TPM) obtained by Salmon of the 211 genes with novel *ONSEN* insertions with the TPM values of four groups of 211 randomly sampled genes, for the three wt (untreated) replicates, in both control and heat stress conditions (Supplementary File S9 for the gene lists and their TPM values). Using a Kruskal-Wallis test with pairwise comparison, we observed that our list of 211 genes with novel *ONSEN* insertion had indeed a significantly lower transcription level than all four randomly sampled gene lists, in both control and heat stress conditions (*P*-value s of 1.644e^–7^ and 1.448e^–5^, respectively). There was, however, no significant difference found between the four randomly sampled gene lists in any condition.

## DISCUSSION

Ever since their initial discovery by Barbara McClintock (53), it was understood that TEs can directly influence gene expression. Next to being potent mutagens, TEs can also modulate gene expression as their mobility can lead to a redistribution and/or amplification of the gene regulatory elements that they carry (11,54–56). These can be enhancers, promoters but also repressive epigenetic marks that the TE attracts (57) and it is probable that a large fraction of genes is under the influence of TEs or remnants thereof (58). Here we wanted to study at the molecular level, how a stress-responsive TE can change the stress-response of its host. While there have been numerous studies and reviews discussing the mutagenic and epigenetic effects of TEs (7,59), so far few detailed studies have been carried out to investigate the direct effect they have on gene expression at novel insertion sites in plants. Using heat stress and drug-induced mobilization of *ONSEN*, we were able to create a collection of Arabidopsis lines carrying varying numbers of novel TE insertions (14). Through whole genome sequencing, we identified the exact insertion sites of this TE. Of the eight ‘full length’ *ONSEN* copies present in the wild-type genome, four copies accounted for 234 out of 237 insertions (Figure 4). These four copies all have a complete open reading frame (ORF) coding for all the proteins necessary for the transposition. Also, *AT1G11265*, *AT4G61330* and *AT5G13205* have perfectly identical LTR sequences; a condition normally essential to allow the retrotransposon to perform its complete lifecycle (60) and which is evidence for its recent mobility. Interestingly, *AT1G48710*, which, in the Arabidopsis TAIR10 genome is shown to have two SNPs unique to its 3′LTR, still contributed to a good proportion of novel *ONSEN* insertions in our hcLines. However, by investigating this specific copy, we observed that the frequency of these two SNPs was 10 times less than the other SNPs unique to *AT1G48710*, hinting that in our wild-type line, this copy probably had identical LTRs. This could have happened through recombination between *ONSEN* copies, as it has been shown previously (61). *AT3G59720*, accounting for only three novel insertions, has perfect LTRs, but does not have a complete ORF; hinting that non-autonomous copies are much less likely to be inserted, even though other autonomous copies are mobilized at the same time. The last three copies, *AT1G21945*, *AT1G58140* and *AT3G32415* have neither identical LTRs, nor an intact ORF, and are very weakly transcribed and seem unable to generate new insertions confirming previous reports (61,62).

Preferential TE insertion in regions enriched for different chromatin states have been documented for a broad range of TEs in animals (63). In plants, preferential insertion sites have so far rather been documented at the sequence level: For instance, the rice mPing transposons integrates preferentially upstream of protein-coding genes (2). In the case of Gypsy LTR retrotransposons it has been proposed that chromodomains encoded by these TEs play a role in targeting those towards heterochromatin (64). Here we found that *ONSEN* had a clear preference for chromatin states rich in H2A.Z (as also documented by (78)) and H3K27me3. Next it would be of great interest to test how *ONSEN* insertion site preference may be modulated in plants defective in H2A.Z (65) and/or H3K27me3 (66) deposition. This would help elucidate if these histone modifications are necessary for targeting *ONSEN* at these sites or if there are other chromatin features guiding it.

It is surprising that *ONSEN* primarily targets exons of genes considering that TEs usually would target genomic niches to reduce the potential negative impact on fitness of the host (67). Zhang *et al.* (68) have proposed two types of TE insertion strategies: (A) Targeting of transcription start sites (TSSs) in association with Pol II mediated transcription or (B) preferential targeting of both TSSs and transcription termination sites of medium expressed genes. The strategy of *ONSEN* may have a somewhat intermediate strategy primarily integrating into genes showing low expression in the tissues we tested. It is notable that *ONSEN* preferentially integrated in genes with the chromatin states 5 and 2 that show a low expression level in adult plants and are often associated with typical polycomb chromatin or repressed regions ((78) and this work). This may explain why we were able to recover sibling lines (hcLine33a and hcLine33d) sharing 99 new insertions in their genome. In terms of size, these insertions add 490 kb to a genome of 119 Mb (0.4% increase in genome size) and confirms previous findings that TEs can contribute to a rapid increase in genome size (69); in this case in a single generation. As the insertion sites observed in the hcLines are similar to the ones previously documented for *nrpd1* and natural populations (44,78), both in terms of chromatin states and genomic features, we concluded that the activation through the exposition to α-amanitin and zebularine did not impact *ONSEN* insertion site preferences.

By looking at the genes where we found novel *ONSEN* insertions in the hcLines, as well as the possible hotspots of insertions, we observed an enrichment in genes related to phosphatidylinositol signaling system, inositol phosphate metabolism, and NAD + biosynthesis (Supplementary Files 6 & 7). These genes and functions have recently been highlighted to play a role in response to abiotic stress in plants (70,71) and it goes in the same direction as a previous observation stating that *ONSEN* preferentially targets environmentally responsive genes (78). However, we have to keep in mind that we can only observe non-lethal or non sterility-inducing insertions in hcLines, and we cannot rule out that other preferential sites for insertions could exist, but are not observed because effects of an insertion at such loci would be too deleterious to be inherited to the progeny.

In our transcriptome analysis, we document that the epigenetic drugs can lead to heritable transcriptional changes, notably at regions previously shown to be epigenetically unstable (48,49). This suggests that the combined drug treatments may have led to stable DNA methylation changes in the treated lines resulting in stochastic transcriptional activation of silent loci. Methylome analysis of these lines will be of great interest to confirm this hypothesis but was outside of the scope of this report centered on the direct TE-induced transcriptional changes.

The *ONSEN* LTRs contain heat-stress response elements that are necessary for its mobilization (62,72). We have previously reported that novel *ONSEN* insertions can lead to the acquisition of heat-stress responsiveness at the affected gene (13), yet the study was limited to qPCR. Using stranded RNA-seq we were now in the position to analyze these findings at much greater detail which also allowed us to uncover other transcriptional changes. Indeed, our analysis on the direct effect of *ONSEN* on transcription revealed a plethora of additional transcriptional changes: knock-out, constitutive activation of gene expression, alternative splicing, creation of ncRNAs, antisense transcription and exonisation (integration of *ONSEN* sequence in the transcribed portion of the gene), just to name a few. Some of the most prominent effects we observed will be discussed here. While we often observed *ONSEN* causing gene truncation, there were numerous cases of *ONSEN* exonisation (Table 3). Transcript fusions between genes and the novel TE insertions within these genes under control conditions most likely explain the *ONSEN* LTR-specific signal we observed (Figure 6A) that can be the result of mismapped reads. The acquisition of constitutive gene expression at *AT1G03710* under control conditions was unexpected. However, since the *ONSEN* insertion results in exon skipping, it likely leads to the complete absence or the biosynthesis of a non-functional protein. This in turn could promote a positive feedback loop increasing this gene's expression. The creation of heat-stress responsive non-coding RNAs (ncRNAs) and antisense transcripts by novel *ONSEN* insertions adds an intriguing layer of complexity. It is conceivable that these novel stress-responsive ncRNAs and antisense RNAs then regulate gene expression via the generation of small interfering RNAs (siRNAs). These siRNAs could then influence gene expression in *cis* and in *trans* via post-transcriptional gene silencing and translation inhibition (73). In summary, these examples show that novel TE insertions can contribute to highly complex responses other than just gene knock-out and stress-induced gene activation.

It has been well-documented that novel TE insertions predominantly have negative effects on their host's fitness (74). In line with this, we observed a broader variability in rosette size in the hcLines with a marked trend to a reduction for this parameter. Unfortunately, the aforementioned high complexity of transcriptional changes in combination with the unexpectedly large number of DEGs in the hcLines precluded us from performing a more in-depth correlative analysis between the observed phenotypes and the transcriptome. Especially hcLine4, which underperforms under control condition, yet performs like controls following a heat stress is of particular interest. To isolate the one causal TE insertion of the 26 novel insertions identified, or the epigenetic change related to this trait, backcrosses to wild-type plants will have to be carried out and the progeny carefully genotyped and epigenotyped.

In our efforts to detect novel TE insertions in the hcLines, we could not detect any TE other than *ONSEN* that was mobilized by our treatments (neither as enrichment in the eccDNA nor as novel insertions). As other transposable elements are known to be active in Arabidopsis, such as the CACTA family (75) and *EVADE*/*ATCOPIA93* (76,77), we were surprised to see that our TE-mobilization method only affects *ONSEN*. This reinforces our idea that TE families can respond to very specific triggers, such as stresses and developmental signals, and that a transient inhibition of the silencing pathways through DNA methylation reduction (zebularine) and RNA polymerase II inhibition (α-amanitin) are not sufficient to release other TEs. This will be a focus for our upcoming work.

In this detailed molecular study, we uncovered the intricate relationship between a transposable element and its host. We found that *ONSEN* has a strong insertion site preference for specific chromatin states, and we unravel the prodigious impact TEs can have on their hosts’ genome and transcriptome. Novel *ONSEN* insertions led to transcriptional modifications going far beyond knockouts and stress responsiveness. It will be of great interest to now study how such novel *ONSEN* insertions impact the ecological competitiveness of its Arabidopsis host in order to assess the adaptive power of TEs.

## DATA AVAILABILITY

The datasets generated and/or analyzed in this study are available in the Zenodo repository (DOI: 10.5281/zenodo.5052057, 10.5281/zenodo.5052099, 10.5281/zenodo.5052019, 10.5281/zenodo.5407606).

## Supplementary Material

gkab828_Supplemental_Files
