# Correction to ‘Genomic impact of stress-induced transposable element mobility in Arabidopsis’

**DOI:** 10.1093/nar/gkab1078

**Published:** 2021-11-08

**Authors:** David Roquis, Marta Robertson, Liang Yu, Michael Thieme, Magdalena Julkowska, Etienne Bucher

**Affiliations:** Plant Breeding and Genetic Resources, Agroscope, 1260 Nyon, Switzerland; Plant Breeding and Genetic Resources, Agroscope, 1260 Nyon, Switzerland; Boyce Thompson Institute, 533 Tower Rd., Ithaca, NY 14853, USA; Institute for Plant and Microbial Biology, University of Zurich, Switzerland; Boyce Thompson Institute, 533 Tower Rd., Ithaca, NY 14853, USA; Plant Breeding and Genetic Resources, Agroscope, 1260 Nyon, Switzerland

The authors wish to correct two citations and add a missing reference (78) as follows (shown in **bold**):

## RESULTS

ONSEN preferentially integrates into coding exons enriched for the H3K27me3 histone mark and H2A.Z histone variant

… We compared our set of novel ONSEN insertions with those previously described in wild plants (natural insertions, Figure 2) and in NRPD1 defective plants (nrpd1 insertions, Figure 2) (44,78) …

… We used our novel hcLines insertions (*n*= 237) as well as the previously identified nrpd1 (*n*= 281) and natural (*n*= 279) insertions (44,78) …

**Figure 2 caption**

Genome-wide distribution of novel ONSEN insertions in the Arabidopsis genome. Novel insertions detected in this study are represented in blue (hcLines) and those previously reported (44,78) for natural populations and nrpd1 plants in grey and orange, respectively. The density plots below the grey chromosome schemes show gene density (green) and TE density (yellow). Units are given in Mb.

## DISCUSSION

… Here we found that ONSEN had a clear preference for chromatin states rich in H2A.Z (as also documented by (78)) and H3K27me3 …

… It is notable that *ONSEN* preferentially integrated in genes with the chromatin states 5 and 2 that show a low expression level in adult plants and are often associated with typical polycomb chromatin

or repressed regions ((78) and this work) …

… As the insertion sites observed in the hcLines are similar to the ones previously documented for nrpd1 and natural populations (44,78), both in terms of chromatin states and genomic features, we concluded that the activation through the exposition to α-amanitin and zebularine did not impact *ONSEN* insertion site preferences …

… These genes and functions have recently been highlighted to play a role in response to abiotic stress in plants (70,71) and it goes in the same direction as a previous observation stating that *ONSEN* preferentially targets environmentally responsive genes (78) …

The article (1) has been updated. The correction does not affect the results, discussion and conclusions presented in the article.
