# Supplementary material for: Genomic impact of stress-induced transposable element mobility in Arabidopsis
Source: Nucleic Acids Res. 2021 Sep 22;49(18):10431–47. doi: 10.1093/nar/gkab828 (PMC8501995; doi:10.1093/nar/gkab828)
Supplement: gkab828_Supplemental_Files [file gkab828_supplemental_files.zip › Supplementary File 1 - qPCR Primers.docx]

**Supplementary File 1**. qPCR primers used to quantify ONSEN in extrachromosomal circular DNA (eccDNA)

| **Species** | **Purpose** | **Primer Name** | **Primer Sequence** |
| --- | --- | --- | --- |
| Arabidopsis thaliana | qPCR | 284 COPIA78-4219F_RT | CCACAAGAGGAACCAACGAA |
|  |  | 285 COPIA78-4219R_RT | TTCGATCATGGAAGACCGG |
|  |  | ONSEN probe (FAM) | AAGTCGGCAATAGCTTTGGCGAAGA (BHQ1) |
|  |  | ACT2_QT_F | TGCCAATCTACGAGGGTTTC |
|  |  | ACT2_QT_R | TTACAATTTCCCGCTCTGCT |
|  |  | ACT2_QT_probe (JOE) | TCCGTCTTGACCTTGCTGGACG  (BHQ-1) |
