# Supplementary material for: Genomic impact of stress-induced transposable element mobility in Arabidopsis
Source: Nucleic Acids Res. 2021 Sep 22;49(18):10431–47. doi: 10.1093/nar/gkab828 (PMC8501995; doi:10.1093/nar/gkab828)
Supplement: gkab828_Supplemental_Files [file gkab828_supplemental_files.zip › Supplementary Materials Legends.docx]

## Supplementary Data

**Supplementary Figure 1** – **Rosette leaf area over time measured by continuous imaging;** Rosette surface average (dashed line) for (**A**) hcLine4, (**B**) hcLine31 and (**C**) hcLine45c compared to HS control.

**Supplementary Figure 2. TSD WebLogos***;* Nucleotide sequence web logos for the target site duplication (TSD) of all novel *ONSEN* insertions in the hclines (portable document file, .pdf).

**Supplementary** **Figure 3**. **Transcriptional and epigenetic changes at *ERT9***. JBrowse screen capture of the locus.

**Supplementary Figure 4. RNA-seq read mapping to the loci of Figure 6B-F**; mapping of the RNA-seq reads on the reconstituted genes to include *ONSEN* insertions.

**Supplementary File 1. qPCR Primers**; Primer sequences for qPCR (Word document, .docx).

**Supplementary File 2. *ATCOPIA78*_Consensus_with_all_InDels**; *ONSEN* consensus sequence generated using the eight “full length” copies of Col-0 genome (FASTA file, .fa).

**Supplementary File 3. *ONSEN* SNPs on Consensus**; List of all SNPs unique to each of the eight “full length” ONSEN copies of Col-0 genome on the consensus sequence from Supplementary File 7 (Excel workbook, .xlsx).

**Supplementary File 4. *ONSEN* qPCR**; qPCR quantification of ONSEN copy numbers in F1 and F4 of the hcLines, as well as correlation with copy number measured through Illumina sequencing (Excel workbook, .xlsx).

**Supplementary File 5. hcLines SNP INDELS CNV**; VCF files for private SNPs, indels and CNVs detected in the hclines, as well as corresponding snpEff annotations in HTML format (compressed archive file containing three VCF and three HTML, .zip).

**Supplementary File 6. *ONSEN* Insertion Characterization**; Detailed characterization of each individual novel *ONSEN* insertions in the hclines (Excel workbook, .xlsx).

**Supplementary File 7. *ONSEN* hotspots**; Characterization of all *ONSEN* possible insertion hotspots (Excel workbook, .xlsx).

**Supplementary File 8. DEG RNA-seq**; List of all differentially expressed genes (DEG) obtained from DESeq2 in the hclines (Excel workbook, .xlsx).

**Supplementary File 9. *ONSEN* and random genes TPM values**; estimated transcription values from Salmon in transcript per million (TPM) in wt under control and heat stress conditions, for genes with novel *ONSEN* insertions as well as four sets of 211 randomly sampled genes, (Excel workbook, .xlsx).
