# Supplementary material for: Genomic impact of stress-induced transposable element mobility in Arabidopsis
Source: Nucleic Acids Res. 2021 Sep 22;49(18):10431–47. doi: 10.1093/nar/gkab828 (PMC8501995; doi:10.1093/nar/gkab828)
Supplement: gkab828_Supplemental_Files [file gkab828_supplemental_files.zip › Supplementary_Figures.pdf]

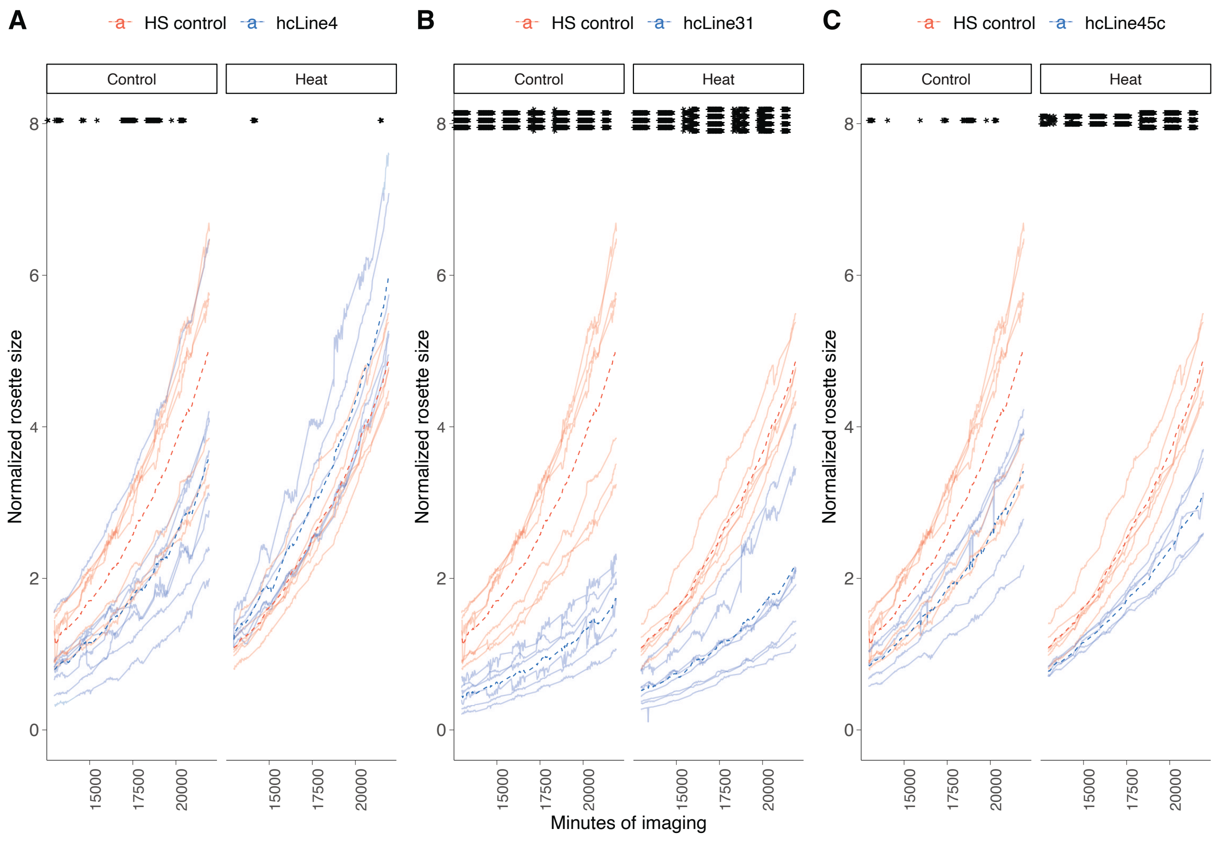

Supplementary Figure 1. Rosette leaf area over time measured by continuous imaging. Rosette surface average (dashed line) for (**A**) hcLine4, (**B**) hcLine31 and (**C**) hcLine45c compared to HS control. The area of the rosettes was monitored every 30 minutes during the 16 h light period for 7 consecutive days. The significant differences between AZ control (red) and individual hcLines (blue) were tested using one-way analysis of variance (ANOVA) and the asterisks indicate the p-value < 0.05 for each timepoint.

ONSEN TSD Sequences

235 x 5bp TSD; 2 x 6 bp TSD

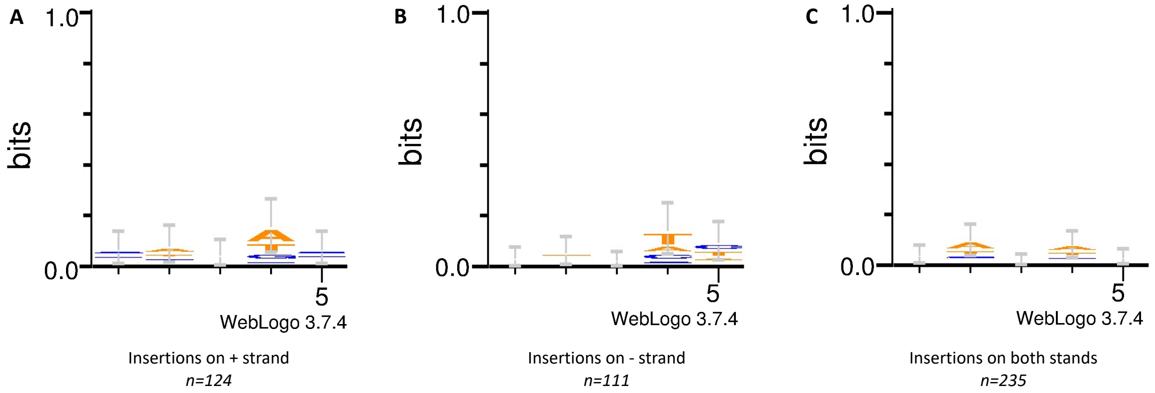

Supplementary Figure 2. Sequence logos of ONSEN TSD. These were generated using the target site duplication (TSD) of the novel ONSEN insertion when ONSEN is A. inserted in sense orientation (+ strand), B. antisense orientation (- strand) or C. all insertions without regard to the orientation.

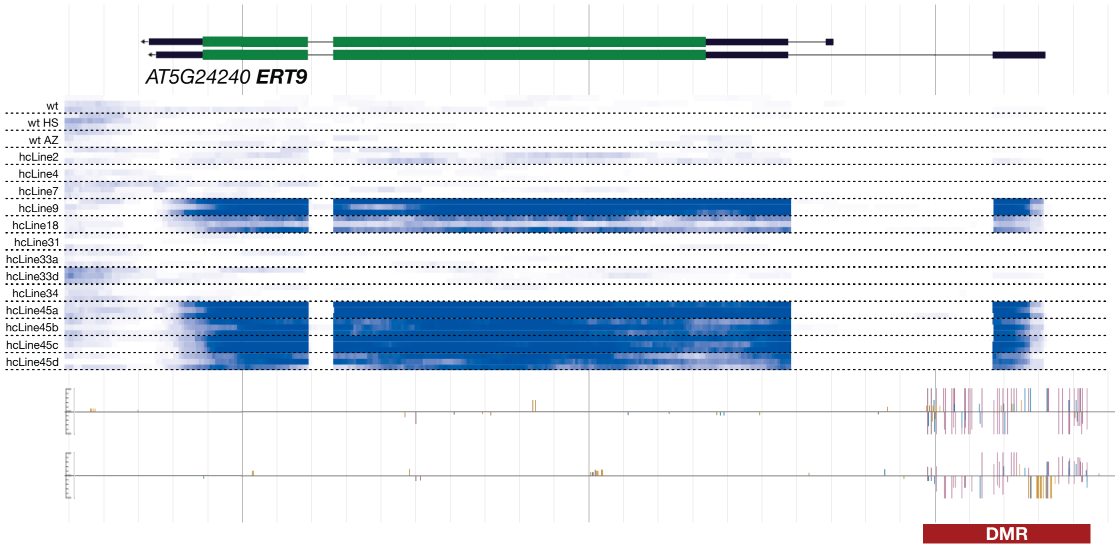

Supplementary Figure 3. Epigenetic drug treatments result in stable transcriptional changes at *ERT9*. The upper part represents genes (coding region in green, UTR in thick black, intron in thin black, arrows indicate the orientation of the transcription). Middle part displays a heat map of transcription based on our RNA-seq data under control conditions (white = no transcription, dark blue >= 200 transcripts per million) for controls and the hcLines (3 biological replicates are shown for each plant line). The lower two histograms show cytosine methylation levels in control (top) and AZ-treated (bottom) plants (color code for the DNA methylation contexts: yellow: CHH, blue: CHG and red: CG). The region with differential DNA methylation is indicated by the red box below.

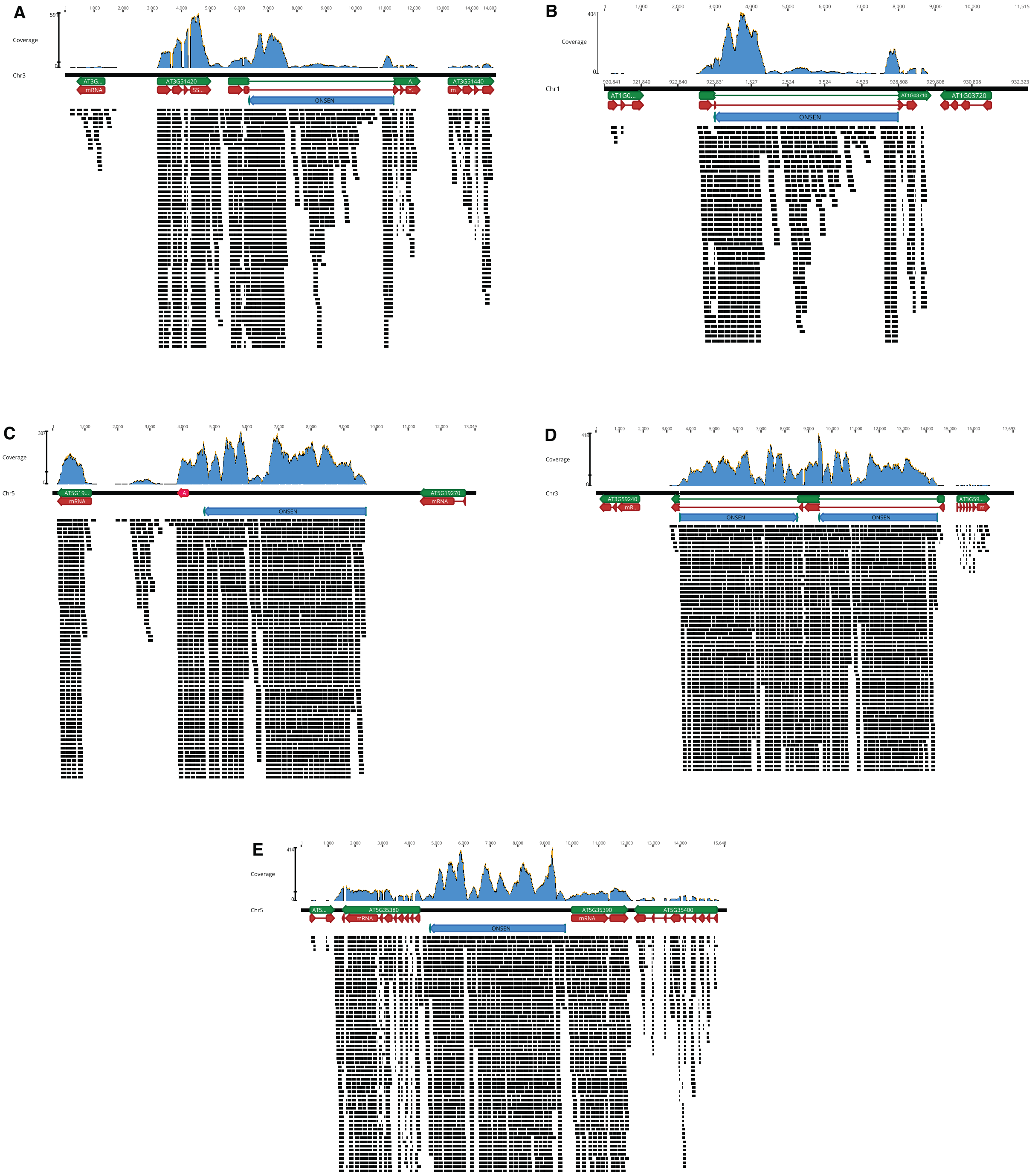

Supplementary Figure 4. RNA-seq read mapping to the loci of Figure 6B-F. Genome browser views showing RNA-seq reads mapping on the reconstituted genes to include *ONSEN* insertions (blue annotation, genes in green and mRNAs in red) under heat stress. The top light blue plot indicates read coverages. (**A**) RNA-seq reads mapped to the *ONSEN* insertion at *AT3G51430* in hcLine33a. (**B**) Reads mapped to *AT1G03710* that carries an *ONSEN* insertion in hcLine33a. (**C**) RNA-seq reads mapped to the locus close to *FAF3* that includes an *ONSEN* insertion in hcLine7. (**D**) Reads mapped to the region around *AT3G59250* containing two *ONSEN* insertions in hcLine7. (**E**) RNA-seq reads mapped to the locus carrying an *ONSEN* insertion between *AT5G35380* and *AT5G35390* in hcLine34.
