# Supplementary material for: Genomic impact of stress-induced transposable element mobility in Arabidopsis
Source: Nucleic Acids Res. 2021 Sep 22;49(18):10431–47. doi: 10.1093/nar/gkab828 (PMC8501995; doi:10.1093/nar/gkab828)
Supplement: gkab828_Supplemental_Files [file gkab828_supplemental_files.zip › Supplementary File 5 - hclines SNP INDELS CNV.zip!/snpEff_hclines_private_CNV.html]

### SnpEff: Variant analysis

**Contents**
Summary  
 Variant rate by chromosome  
Variants by type  
 Number of variants by impact   
 Number of variants by functional class   
 Number of variants by effect   
Quality histogram  
InDel length histogram  
Base variant table  
Transition vs transversions (ts/tv)  
 Allele frequency   
 Allele Count   
 Codon change table   
 Amino acid change table   
 Chromosome variants plots   
 Details by gene


---


**Summary**

|  |  |
| --- | --- |
| **Genome** | Arabidopsis\_thaliana |
| **Date** | 2021-06-28 14:58 |
| **SnpEff version** | ``` SnpEff 5.0e (build 2021-03-09 06:01), by Pablo Cingolani ``` |
| **Command line arguments** | ``` SnpEff  Arabidopsis_thaliana hclines_private_CNV.vcf ``` |
| **Warnings** | 224 |
| **Errors** | 600 |
| **Number of lines (input file)** | 1,753 |
| **Number of variants (before filter)** | 1,753 |
| **Number of not variants  (i.e. reference equals alternative)** | 0 |
| **Number of variants processed   (i.e. after filter and non-variants)** | 1,731 |
| **Number of known variants  (i.e. non-empty ID)** | 1,731 ( 100% ) |
| **Number of multi-allelic VCF entries  (i.e. more than two alleles)** | 0 |
| **Number of effects** | 8,498 |
| **Genome total length** | 119,667,774 |
| **Genome effective length** | 119,146,350 |
| **Variant rate** | 1 variant every 68,830 bases |


---


 **Variants rate details** 

| Chromosome | Length | Variants | Variants rate |
| --- | --- | --- | --- |
| 1 | 30,427,671 | 266 | 114,389 |
| 2 | 19,698,289 | 198 | 99,486 |
| 3 | 23,459,830 | 221 | 106,153 |
| 4 | 18,585,056 | 194 | 95,799 |
| 5 | 26,975,502 | 252 | 107,045 |
| chloroplast | 1 | 0 | 0 |
| mitochondria | 1 | 0 | 0 |
| Total | 119,146,350 | 1,731 | 68,830 |


---


 **Number variants by type**

| **Type** | **Total** |
| --- | --- |
| **SNP** | 0 |
| **MNP** | 0 |
| **INS** | 1 |
| **DEL** | 180 |
| **MIXED** | 0 |
| **INV** | 0 |
| **DUP** | 1,550 |
| **BND** | 0 |
| **INTERVAL** | 0 |
| **Total** | 1,731 |
| --- | --- |


---


 **Number of effects by impact** 

| **Type (alphabetical order)** |  | Count | Percent |
| --- | --- | --- | --- |
| **HIGH** |  | 1,564 | 19.802% |
| **LOW** |  | 102 | 1.291% |
| **MODERATE** |  | 18 | 0.228% |
| **MODIFIER** |  | 6,214 | 78.678% |


---


 **Number of effects by functional class** 

| **Type (alphabetical order)** |  | Count | Percent |
| --- | --- | --- | --- |

  

Missense / Silent ratio: 0


---


 **Number of effects by type and region** 

| Type | Region |
| --- | --- |
| **Type (alphabetical order)** \| \| Count \| Percent; **3\_prime\_UTR\_truncation** \| \| 17 \| 0.188%; **3\_prime\_UTR\_variant** \| \| 99 \| 1.094%; **5\_prime\_UTR\_truncation** \| \| 6 \| 0.066%; **5\_prime\_UTR\_variant** \| \| 75 \| 0.829%; **bidirectional\_gene\_fusion** \| \| 109 \| 1.204%; **conservative\_inframe\_deletion** \| \| 26 \| 0.287%; **disruptive\_inframe\_deletion** \| \| 3 \| 0.033%; **downstream\_gene\_variant** \| \| 2,562 \| 28.303%; **duplication** \| \| 488 \| 5.391%; **exon\_loss\_variant** \| \| 508 \| 5.612%; **exon\_region** \| \| 91 \| 1.005%; **feature\_ablation** \| \| 5 \| 0.055%; **frameshift\_variant** \| \| 351 \| 3.878%; **gene\_fusion** \| \| 62 \| 0.685%; **intergenic\_region** \| \| 616 \| 6.805%; **intragenic\_variant** \| \| 57 \| 0.63%; **intron\_variant** \| \| 507 \| 5.601%; **non\_coding\_transcript\_exon\_variant** \| \| 16 \| 0.177%; **non\_coding\_transcript\_variant** \| \| 75 \| 0.829%; **splice\_acceptor\_variant** \| \| 122 \| 1.348%; **splice\_donor\_variant** \| \| 226 \| 2.497%; **splice\_region\_variant** \| \| 401 \| 4.43%; **start\_lost** \| \| 29 \| 0.32%; **stop\_gained** \| \| 60 \| 0.663%; **stop\_lost** \| \| 48 \| 0.53%; **transcript\_ablation** \| \| 49 \| 0.541%; **upstream\_gene\_variant** \| \| 2,444 \| 27% | **Type (alphabetical order)** \| \| Count \| Percent; **DOWNSTREAM** \| \| 2,561 \| 32.426%; **EXON** \| \| 1,358 \| 17.194%; **GENE** \| \| 178 \| 2.254%; **INTERGENIC** \| \| 616 \| 7.799%; **INTRON** \| \| 151 \| 1.912%; **SPLICE\_SITE\_ACCEPTOR** \| \| 49 \| 0.62%; **SPLICE\_SITE\_DONOR** \| \| 104 \| 1.317%; **SPLICE\_SITE\_REGION** \| \| 88 \| 1.114%; **TRANSCRIPT** \| \| 185 \| 2.342%; **UPSTREAM** \| \| 2,444 \| 30.945%; **UTR\_3\_PRIME** \| \| 99 \| 1.253%; **UTR\_5\_PRIME** \| \| 65 \| 0.823% |


---


 **Quality:**

```
|  |  |
| --- | --- |
| Min | -1 |
| Max | -1 |
| Mean | -1 |
| Median | -1 |
| Standard deviation | 0 |
| Values | -1 |
| Count | 1731 |
```


---


 **Insertions and deletions length:**

```
|  |  |
| --- | --- |
| Min | 1 |
| Max | 68 |
| Mean | 1.37 |
| Median | 1 |
| Standard deviation | 4.98 |
| Values | 1,68 |
| Count | 180,1 |
```


---


 **Base changes (SNPs)** 

|  |  |  |  |  |
| --- | --- | --- | --- | --- |
|  | **A** | **C** | **G** | **T** |
| **A** | 0 | 0 | 0 | 0 |
| **C** | 0 | 0 | 0 | 0 |
| **G** | 0 | 0 | 0 | 0 |
| **T** | 0 | 0 | 0 | 0 |

---


  **Ts/Tv (transitions / transversions)** 


**Note:** Only SNPs are used for this statistic.  
**Note:** This Ts/Tv ratio is a 'raw' ratio (ratio of observed events).

|  |  |
| --- | --- |
| Transitions | 0 |
| Transversions | 0 |
| Ts/Tv ratio | 0 |

**All variants:**

```
No results available (empty input?)
```

**Only known variants** (i.e. the ones having a non-empty ID field):

```
No results available (empty input?)
```

---


  **Allele frequency** 

  

|  |  |
| --- | --- |
| Min | 0 |
| Max | 0 |
| Mean | 0 |
| Median | 0 |
| Standard deviation | 0 |
| Values | 0 |
| Count | 1731 |

---


  **Allele Count** 

  

|  |  |
| --- | --- |
| Min | 0 |
| Max | 0 |
| Mean | 0 |
| Median | 0 |
| Standard deviation | 0 |
| Values | 0 |
| Count | 1731 |

---


  **Hom/Het per sample** 

  
  
  

```
Sample_names 
Reference 
Het 
Hom 
Missing
```

---


 **Codon changes**

How to read this table:   
- Rows are reference codons and columns are changed codons. E.g. Row 'AAA' column 'TAA' indicates how many 'AAA' codons have been replaced by 'TAA' codons.  
- Red background colors indicate that more changes happened (heat-map).  
- Diagonals are indicated using grey background color   
- WARNING: This table may include different translation codon tables (e.g. mamalian DNA and mitochondrial DNA).

|  | - | AAA | AAC | AAG | AAN | AAT | ACA | ACC | ACG | ACT | AGA | AGC | AGG | AGT | ANG | ATA | ATC | ATG | ATT | CAA | CAC | CAG | CAT | CCA | CCC | CCG | CCT | CGA | CGC | CGG | CGT | CTA | CTC | CTG | CTT | GAA | GAC | GAG | GAT | GCA | GCC | GCG | GCT | GGA | GGC | GGG | GGT | GTA | GTC | GTG | GTT | TAA | TAC | TAG | TAT | TCA | TCC | TCG | TCT | TGA | TGC | TGG | TGT | TTA | TTC | TTG | TTT |
| --- | --- | --- | --- | --- | --- | --- | --- | --- | --- | --- | --- | --- | --- | --- | --- | --- | --- | --- | --- | --- | --- | --- | --- | --- | --- | --- | --- | --- | --- | --- | --- | --- | --- | --- | --- | --- | --- | --- | --- | --- | --- | --- | --- | --- | --- | --- | --- | --- | --- | --- | --- | --- | --- | --- | --- | --- | --- | --- | --- | --- | --- | --- | --- | --- | --- | --- | --- |
| - |  | 354 | 195 | 395 |  | 224 | 294 | 150 | 100 | 223 | 462 | 220 | 234 | 269 |  | 188 | 174 | 216 | 142 | 369 | 137 | 179 | 275 | 201 | 75 | 81 | 174 | 106 | 57 | 81 | 103 | 172 | 253 | 135 | 337 | 335 | 115 | 238 | 180 | 215 | 132 | 66 | 290 | 519 | 209 | 110 | 409 | 153 | 132 | 158 | 390 | 173 | 141 | 115 | 170 | 245 | 194 | 127 | 252 | 374 | 226 | 345 | 211 | 221 | 224 | 362 | 245 |
| AAA | 752 | 236 | 155 | 242 |  | 122 | 172 | 91 | 48 | 94 | 311 | 189 | 161 | 98 |  | 129 | 128 | 199 | 121 | 201 | 134 | 92 | 111 | 74 | 29 | 42 | 96 | 84 | 62 | 63 | 64 | 114 | 129 | 149 | 123 | 239 | 57 | 118 | 96 | 85 | 42 | 27 | 92 | 191 | 34 | 33 | 104 | 61 | 51 | 127 | 71 | 157 | 71 | 129 | 121 | 133 | 82 | 88 | 139 | 265 | 88 | 195 | 164 | 110 | 162 | 203 | 154 |
| AAC | 354 | 104 | 59 | 132 |  | 58 | 105 | 58 | 35 | 42 | 156 | 54 | 72 | 83 |  | 32 | 67 | 92 | 82 | 121 | 68 | 100 | 109 | 43 | 16 | 49 | 42 | 52 | 19 | 22 | 24 | 66 | 65 | 46 | 73 | 163 | 69 | 69 | 60 | 44 | 29 | 17 | 18 | 116 | 51 | 48 | 16 | 65 | 48 | 114 | 45 | 74 | 59 | 47 | 73 | 91 | 67 | 32 | 83 | 154 | 92 | 145 | 87 | 53 | 99 | 66 | 91 |
| AAG | 1,273 | 320 | 169 | 281 |  | 157 | 184 | 72 | 50 | 186 | 363 | 197 | 127 | 90 |  | 140 | 115 | 167 | 137 | 314 | 114 | 134 | 99 | 103 | 60 | 37 | 33 | 54 | 54 | 66 | 52 | 108 | 165 | 84 | 196 | 249 | 53 | 87 | 96 | 94 | 44 | 54 | 97 | 269 | 83 | 51 | 88 | 104 | 49 | 154 | 127 | 158 | 77 | 99 | 81 | 141 | 91 | 26 | 102 | 314 | 144 | 221 | 142 | 145 | 141 | 285 | 124 |
| AAN | 2 |  |  |  |  |  | 1 |  |  |  |  | 5 |  |  |  |  |  |  |  |  |  |  |  |  |  |  |  |  |  |  |  |  |  |  | 1 |  |  |  |  |  |  |  |  |  |  |  |  |  |  |  |  |  |  |  |  |  |  |  |  |  |  |  |  |  |  | 1 |  |
| AAT | 308 | 229 | 109 | 175 |  | 77 | 77 | 63 | 52 | 66 | 197 | 91 | 103 | 44 |  | 80 | 116 | 128 | 106 | 196 | 78 | 73 | 79 | 85 | 10 | 53 | 48 | 46 | 35 | 56 | 35 | 93 | 67 | 122 | 192 | 191 | 67 | 78 | 102 | 47 | 75 | 48 | 47 | 131 | 31 | 66 | 49 | 61 | 63 | 78 | 103 | 129 | 52 | 35 | 100 | 131 | 88 | 70 | 117 | 190 | 122 | 91 | 86 | 87 | 209 | 138 | 103 |
| ACA | 377 | 120 | 82 | 95 |  | 51 | 67 | 51 | 16 | 34 | 89 | 81 | 78 | 41 |  | 44 | 78 | 87 | 59 | 107 | 94 | 116 | 114 | 45 | 28 | 21 | 48 | 27 | 31 | 27 | 73 | 51 | 51 | 66 | 84 | 149 | 43 | 90 | 49 | 57 | 41 | 26 | 33 | 59 | 20 | 20 | 41 | 24 | 44 | 61 | 54 | 53 | 87 | 63 | 55 | 110 | 58 | 11 | 70 | 99 | 68 | 121 | 103 | 51 | 76 | 95 | 90 |
| ACC | 311 | 27 | 70 | 55 |  | 33 | 74 | 31 | 21 | 43 | 102 | 28 | 46 | 30 |  | 32 | 43 | 28 | 33 | 96 | 21 | 29 | 24 | 53 | 24 | 11 | 10 | 24 | 1 | 28 | 16 | 32 | 56 | 24 | 29 | 36 | 17 | 13 | 38 | 22 | 20 | 4 | 18 | 25 | 21 | 24 | 28 | 13 | 4 | 51 | 11 | 15 | 24 | 29 | 26 | 47 | 20 | 10 | 36 | 60 | 54 | 58 | 20 | 8 | 63 | 66 | 42 |
| ACG | 129 | 40 | 30 | 51 |  | 17 | 19 | 6 | 6 | 22 | 47 | 50 | 19 | 6 |  | 26 | 14 | 62 | 19 | 41 | 20 | 22 | 33 | 2 | 1 | 26 | 4 | 15 | 26 | 14 | 10 | 24 | 46 | 25 | 12 | 39 | 18 | 20 | 16 | 26 |  | 6 | 6 | 19 | 11 | 31 | 9 | 18 | 15 | 34 | 32 | 11 | 32 | 21 | 19 | 55 | 28 | 18 | 15 | 40 | 33 | 23 | 29 | 26 | 17 | 53 | 58 |
| ACT | 361 | 137 | 103 | 103 |  | 54 | 113 | 50 | 59 | 50 | 177 | 81 | 84 | 64 |  | 67 | 70 | 137 | 57 | 142 | 117 | 47 | 84 | 42 | 18 | 31 | 32 | 32 | 19 | 31 | 33 | 51 | 54 | 92 | 54 | 110 | 100 | 56 | 55 | 58 | 47 | 13 | 18 | 100 | 35 | 48 | 56 | 44 | 86 | 63 | 80 | 96 | 67 | 64 | 91 | 92 | 44 | 90 | 56 | 149 | 65 | 131 | 115 | 54 | 84 | 127 | 82 |
| AGA | 514 | 149 | 75 | 191 |  | 60 | 90 | 23 | 47 | 87 | 140 | 104 | 92 | 66 |  | 92 | 72 | 108 | 57 | 130 | 40 | 119 | 74 | 46 | 22 | 32 | 43 | 52 | 12 | 31 | 66 | 70 | 139 | 63 | 102 | 163 | 45 | 71 | 60 | 53 | 37 | 8 | 82 | 117 | 36 | 23 | 63 | 14 | 68 | 29 | 61 | 46 | 43 | 58 | 98 | 76 | 67 | 38 | 53 | 154 | 95 | 88 | 102 | 43 | 92 | 113 | 137 |
| AGC | 320 | 96 | 28 | 103 |  | 67 | 54 | 33 | 19 | 37 | 124 | 58 | 32 | 23 | 1 | 62 | 57 | 57 | 60 | 66 | 7 | 58 | 40 | 26 | 7 | 9 | 26 | 36 | 21 | 10 | 10 | 32 | 19 | 54 | 74 | 63 | 22 | 35 | 34 | 53 | 14 | 11 | 57 | 50 | 14 | 20 | 27 | 8 | 38 | 38 | 64 | 41 | 18 | 53 | 54 | 48 | 37 | 30 | 58 | 116 | 16 | 63 | 42 | 76 | 83 | 95 | 87 |
| AGG | 109 | 64 | 36 | 92 |  | 35 | 35 | 28 | 27 | 18 | 86 | 37 | 10 | 64 |  | 19 | 37 | 57 | 32 | 54 | 40 | 59 | 37 | 44 | 1 | 26 | 31 | 21 | 2 | 21 | 2 | 24 | 27 | 56 | 32 | 57 | 20 | 50 | 25 | 42 | 11 | 6 | 17 | 66 | 41 | 21 | 21 | 15 | 34 | 26 | 33 | 12 | 7 | 55 | 25 | 23 | 68 | 18 | 48 | 107 | 58 | 34 | 55 | 35 | 22 | 75 | 78 |
| AGT | 285 | 107 | 82 | 87 |  | 41 | 49 | 37 | 16 | 56 | 120 | 70 | 58 | 49 |  | 40 | 92 | 83 | 20 | 89 | 38 | 76 | 39 | 56 | 33 | 13 | 35 | 45 | 19 | 32 | 30 | 41 | 38 | 62 | 85 | 126 | 45 | 109 | 69 | 40 | 59 | 16 | 25 | 79 | 24 | 34 | 66 | 34 | 50 | 50 | 52 | 47 | 23 | 86 | 55 | 41 | 31 | 37 | 52 | 90 | 71 | 103 | 87 | 37 | 89 | 86 | 94 |
| ANG |  |  |  |  |  |  |  |  |  |  |  |  |  |  |  |  |  |  |  |  |  |  |  |  |  |  |  |  |  |  |  |  |  |  |  |  |  |  |  |  |  |  |  |  |  |  |  |  |  |  |  |  |  |  |  |  |  |  |  |  |  |  |  |  |  |  |  |
| ATA | 238 | 125 | 36 | 81 |  | 106 | 26 | 37 | 28 | 55 | 128 | 47 | 53 | 41 |  | 57 | 80 | 65 | 76 | 99 | 56 | 68 | 78 | 20 | 10 | 6 | 34 | 18 | 6 | 27 | 19 | 60 | 72 | 53 | 58 | 76 | 21 | 62 | 58 | 55 | 12 | 20 | 30 | 95 | 4 | 38 | 40 | 31 | 32 | 38 | 44 | 71 | 55 | 44 | 47 | 84 | 37 | 14 | 82 | 174 | 45 | 68 | 95 | 66 | 71 | 70 | 88 |
| ATC | 243 | 74 | 69 | 105 |  | 78 | 72 | 32 | 22 | 22 | 76 | 60 | 99 | 46 |  | 33 | 43 | 99 | 58 | 97 | 21 | 31 | 88 | 14 | 21 | 30 | 13 | 25 | 4 | 47 | 20 | 51 | 70 | 69 | 67 | 51 | 30 | 54 | 44 | 58 | 46 | 6 | 47 | 59 | 24 | 5 | 28 | 36 | 21 | 57 | 79 | 55 | 77 | 28 | 62 | 68 | 57 | 44 | 39 | 85 | 43 | 75 | 62 | 37 | 83 | 77 | 46 |
| ATG | 636 | 153 | 49 | 130 |  | 114 | 115 | 57 | 16 | 68 | 249 | 68 | 129 | 66 |  | 90 | 93 | 103 | 88 | 221 | 84 | 110 | 117 | 71 | 34 | 35 | 63 | 40 | 21 | 51 | 29 | 64 | 132 | 60 | 116 | 168 | 77 | 90 | 93 | 84 | 43 | 25 | 79 | 61 | 67 | 71 | 83 | 26 | 54 | 85 | 85 | 87 | 29 | 90 | 83 | 109 | 85 | 55 | 118 | 221 | 78 | 126 | 97 | 58 | 24 | 166 | 51 |
| ATT | 382 | 82 | 107 | 112 |  | 93 | 82 | 35 | 22 | 72 | 205 | 87 | 101 | 56 | 1 | 54 | 97 | 188 | 72 | 89 | 67 | 111 | 95 | 75 | 27 | 34 | 33 | 88 | 26 | 33 | 38 | 46 | 70 | 68 | 78 | 112 | 50 | 97 | 110 | 73 | 27 | 16 | 38 | 126 | 58 | 45 | 30 | 42 | 55 | 56 | 47 | 97 | 70 | 75 | 113 | 138 | 84 | 45 | 86 | 163 | 122 | 148 | 71 | 64 | 86 | 122 | 110 |
| CAA | 258 | 96 | 101 | 111 |  | 80 | 122 | 71 | 42 | 44 | 147 | 133 | 138 | 39 |  | 42 | 107 | 71 | 103 | 116 | 46 | 66 | 75 | 94 | 38 | 38 | 38 | 30 | 13 | 43 | 62 | 74 | 79 | 92 | 83 | 123 | 64 | 76 | 55 | 81 | 65 | 19 | 37 | 72 | 38 | 21 | 52 | 63 | 66 | 82 | 98 | 59 | 26 | 94 | 61 | 115 | 94 | 28 | 90 | 182 | 41 | 142 | 79 | 60 | 97 | 98 | 112 |
| CAC | 112 | 27 | 26 | 51 |  | 39 | 45 | 20 | 6 | 62 | 122 | 31 | 20 | 29 |  | 24 | 24 | 26 | 22 | 52 | 16 | 51 | 49 | 38 | 12 | 12 | 14 | 32 | 9 | 18 | 3 | 10 | 44 | 35 | 26 | 21 | 10 | 34 | 20 | 23 | 11 | 2 | 37 | 29 | 30 | 62 | 12 | 20 | 8 | 30 | 16 | 39 | 15 | 13 | 17 | 63 | 35 | 7 | 17 | 42 | 66 | 65 | 10 | 27 | 39 | 44 | 42 |
| CAG | 157 | 91 | 101 | 73 |  | 78 | 68 | 32 | 39 | 20 | 110 | 73 | 66 | 34 |  | 26 | 81 | 116 | 67 | 99 | 43 | 101 | 68 | 36 | 12 | 8 | 77 | 29 | 12 | 34 | 28 | 22 | 73 | 62 | 80 | 86 | 28 | 103 | 71 | 56 | 11 | 23 | 38 | 73 | 11 | 29 | 15 | 31 | 36 | 51 | 69 | 74 | 33 | 46 | 10 | 110 | 46 | 15 | 87 | 106 | 89 | 78 | 60 | 51 | 125 | 88 | 107 |
| CAT | 348 | 115 | 50 | 78 |  | 53 | 94 | 34 | 13 | 79 | 108 | 78 | 59 | 60 |  | 73 | 48 | 57 | 44 | 127 | 56 | 53 | 68 | 94 | 11 | 23 | 52 | 41 | 15 | 25 | 2 | 44 | 117 | 107 | 79 | 120 | 20 | 59 | 88 | 55 | 11 | 22 | 47 | 60 | 56 | 36 | 60 | 26 | 24 | 40 | 74 | 79 | 23 | 41 | 68 | 139 | 54 | 16 | 26 | 94 | 81 | 103 | 72 | 21 | 61 | 68 | 102 |
| CCA | 477 | 142 | 92 | 172 |  | 116 | 80 | 90 | 24 | 56 | 168 | 80 | 80 | 34 | 3 | 68 | 80 | 148 | 58 | 120 | 81 | 88 | 63 | 39 | 70 | 49 | 70 | 39 | 42 | 35 | 26 | 41 | 117 | 102 | 34 | 119 | 29 | 40 | 49 | 64 | 67 | 36 | 37 | 59 | 40 | 39 | 34 | 28 | 47 | 41 | 37 | 81 | 50 | 59 | 42 | 73 | 97 | 27 | 60 | 158 | 112 | 139 | 90 | 93 | 87 | 106 | 51 |
| CCC | 71 | 69 | 26 | 27 |  | 8 | 34 | 25 | 4 | 22 | 30 | 6 | 10 | 14 |  | 4 | 21 | 53 | 34 | 43 | 8 | 18 | 31 | 12 | 10 |  | 7 |  | 2 | 14 | 21 | 11 | 13 | 10 | 7 | 19 | 17 | 29 | 36 | 18 | 15 |  | 6 | 29 | 2 | 12 | 18 |  | 17 | 24 | 10 | 22 | 2 | 15 | 12 | 21 | 40 | 2 | 59 | 30 | 14 | 37 | 8 | 18 | 16 | 30 | 6 |
| CCG | 142 | 39 | 54 | 25 |  | 16 | 14 | 19 | 14 | 29 | 51 | 28 | 14 | 11 |  | 29 | 17 | 42 | 28 | 34 | 14 | 25 | 22 | 13 | 18 | 2 | 18 | 14 | 1 | 35 | 7 | 19 | 19 | 8 | 26 | 22 | 18 | 57 | 25 | 15 | 22 |  | 16 | 32 | 10 | 21 | 21 | 8 | 23 | 10 | 25 | 10 | 16 | 20 | 22 | 34 | 13 | 6 | 4 | 71 | 19 | 50 | 33 | 15 | 37 | 30 | 17 |
| CCT | 294 | 116 | 41 | 113 |  | 78 | 104 | 81 | 30 | 82 | 150 | 43 | 87 | 42 |  | 74 | 48 | 96 | 80 | 108 | 57 | 88 | 45 | 32 | 23 | 32 | 26 | 40 | 10 | 41 | 24 | 77 | 41 | 100 | 85 | 77 | 70 | 85 | 47 | 70 | 35 | 18 | 41 | 90 | 28 | 65 | 28 | 36 | 48 | 31 | 49 | 54 | 79 | 83 | 39 | 99 | 172 | 29 | 67 | 93 | 73 | 130 | 87 | 37 | 122 | 72 | 77 |
| CGA | 99 | 36 | 38 | 22 |  | 23 | 14 | 21 | 22 | 18 | 52 | 51 | 17 | 19 |  | 18 | 18 | 14 | 8 | 35 | 18 | 4 | 34 | 14 | 1 | 11 | 16 | 5 | 6 | 22 | 6 | 16 | 19 | 25 | 17 | 50 | 9 | 39 | 4 | 4 | 5 | 7 | 21 | 28 | 19 | 18 | 11 | 8 | 14 | 22 | 22 | 31 |  | 18 | 20 | 27 | 22 | 32 | 11 | 42 | 72 | 56 | 52 | 21 | 34 | 26 | 13 |
| CGC | 44 | 29 | 15 | 20 |  | 4 | 4 | 6 | 12 | 18 | 39 | 2 | 16 | 20 |  | 16 | 17 |  | 12 | 18 | 10 | 22 | 6 | 28 | 2 | 2 | 6 | 21 | 1 | 4 | 4 | 8 | 13 | 17 | 10 | 19 |  | 4 | 6 | 17 | 10 |  | 8 | 24 |  | 8 | 7 | 8 | 16 | 12 | 2 | 18 | 16 | 6 | 12 | 4 | 18 | 14 | 13 | 31 | 18 | 31 | 22 | 8 | 23 | 14 | 14 |
| CGG | 65 | 20 | 22 | 24 |  | 29 | 28 | 12 | 16 | 12 | 20 | 4 | 21 | 26 |  | 10 | 13 | 10 | 29 | 33 | 2 | 22 | 17 | 10 | 4 | 4 |  | 22 | 4 | 10 | 16 | 27 | 26 | 20 | 60 | 11 | 14 | 20 | 11 | 29 | 1 | 4 | 5 | 31 | 8 | 18 | 30 | 14 | 12 |  | 15 | 25 | 17 | 32 | 17 | 23 | 9 | 16 | 12 | 33 | 24 | 30 | 31 | 24 | 22 | 21 | 33 |
| CGT | 118 | 39 | 36 | 57 |  | 79 | 39 | 11 | 16 | 23 | 68 | 8 | 61 | 23 |  | 31 | 47 | 99 | 31 | 22 | 15 | 23 | 50 | 10 | 5 | 18 | 36 |  | 7 | 2 | 4 | 15 | 25 | 43 | 22 | 36 | 6 | 15 | 36 | 33 | 48 | 12 | 11 | 26 | 20 | 8 | 10 | 8 | 15 | 24 | 14 | 36 | 37 | 38 | 33 | 36 | 31 | 45 | 41 | 104 | 17 | 75 | 23 | 33 | 26 | 56 | 18 |
| CTA | 201 | 85 | 26 | 33 |  | 47 | 43 | 16 | 6 | 38 | 127 | 36 | 57 | 48 |  | 23 | 61 | 69 | 58 | 32 | 40 | 35 | 44 | 30 | 26 | 20 | 56 | 45 | 12 | 10 | 30 | 14 | 23 | 50 | 39 | 54 | 29 | 41 | 24 | 31 | 20 | 15 | 36 | 39 | 16 | 17 | 15 | 28 | 36 | 28 | 25 | 42 | 32 | 18 | 41 | 45 | 16 | 33 | 68 | 80 | 64 | 59 | 29 | 23 | 46 | 88 | 36 |
| CTC | 262 | 59 | 34 | 61 |  | 51 | 65 | 26 | 24 | 60 | 88 | 42 | 55 | 33 |  | 40 | 43 | 102 | 32 | 69 | 40 | 68 | 36 | 35 | 15 | 12 | 25 | 31 | 24 | 75 | 25 | 51 | 27 | 44 | 69 | 42 | 20 | 46 | 42 | 12 | 52 | 23 | 20 | 31 | 15 | 15 | 28 | 46 | 35 | 38 | 43 | 53 | 41 | 38 | 64 | 58 | 48 | 34 | 73 | 85 | 60 | 73 | 79 | 48 | 57 | 46 | 55 |
| CTG | 176 | 75 | 41 | 41 |  | 49 | 57 | 20 | 21 | 34 | 80 | 40 | 62 | 37 |  | 17 | 34 | 36 | 21 | 65 | 20 | 19 | 16 | 32 | 13 | 20 | 30 | 18 | 2 | 22 | 7 | 35 | 33 | 60 | 60 | 88 | 51 | 35 | 27 | 62 | 9 | 14 | 35 | 51 | 10 | 23 | 29 | 30 | 24 | 21 | 48 | 78 | 22 | 57 | 55 | 88 | 31 | 28 | 99 | 126 | 102 | 66 | 61 | 16 | 62 | 76 | 54 |
| CTT | 419 | 148 | 119 | 142 |  | 94 | 96 | 61 | 45 | 128 | 189 | 67 | 131 | 90 | 1 | 76 | 97 | 148 | 78 | 144 | 49 | 93 | 60 | 38 | 21 | 24 | 48 | 41 | 35 | 54 | 38 | 82 | 123 | 94 | 86 | 142 | 46 | 72 | 56 | 59 | 35 | 35 | 137 | 127 | 40 | 22 | 57 | 76 | 63 | 64 | 90 | 77 | 74 | 44 | 102 | 113 | 91 | 48 | 190 | 157 | 151 | 143 | 59 | 110 | 134 | 136 | 113 |
| GAA | 557 | 193 | 170 | 224 |  | 139 | 173 | 83 | 83 | 82 | 351 | 159 | 154 | 159 |  | 90 | 142 | 194 | 138 | 250 | 122 | 117 | 112 | 56 | 41 | 43 | 45 | 103 | 32 | 65 | 58 | 104 | 144 | 177 | 180 | 207 | 97 | 134 | 102 | 161 | 59 | 70 | 62 | 211 | 67 | 101 | 58 | 95 | 87 | 148 | 106 | 159 | 107 | 105 | 168 | 217 | 117 | 87 | 140 | 329 | 134 | 199 | 193 | 132 | 191 | 226 | 158 |
| GAC | 562 | 103 | 56 | 128 |  | 52 | 101 | 50 | 22 | 86 | 201 | 86 | 76 | 34 |  | 96 | 81 | 94 | 77 | 97 | 57 | 64 | 75 | 97 | 10 | 34 | 43 | 33 | 28 | 50 | 18 | 68 | 95 | 65 | 70 | 91 | 39 | 58 | 50 | 45 | 31 | 26 | 56 | 98 | 18 | 73 | 40 | 30 | 23 | 91 | 56 | 86 | 30 | 28 | 59 | 81 | 25 | 52 | 67 | 162 | 45 | 103 | 68 | 66 | 57 | 99 | 104 |
| GAG | 352 | 247 | 80 | 196 |  | 158 | 163 | 57 | 61 | 73 | 257 | 136 | 134 | 96 |  | 103 | 107 | 145 | 118 | 173 | 104 | 138 | 83 | 101 | 16 | 41 | 82 | 96 | 22 | 78 | 61 | 93 | 81 | 116 | 112 | 140 | 83 | 85 | 142 | 69 | 48 | 14 | 92 | 170 | 65 | 49 | 102 | 37 | 63 | 94 | 91 | 109 | 83 | 97 | 122 | 105 | 127 | 51 | 113 | 279 | 105 | 228 | 127 | 78 | 105 | 167 | 100 |
| GAT | 546 | 226 | 129 | 173 |  | 128 | 154 | 109 | 47 | 114 | 376 | 171 | 218 | 134 |  | 106 | 118 | 198 | 107 | 276 | 87 | 117 | 183 | 129 | 75 | 33 | 64 | 101 | 41 | 67 | 43 | 133 | 185 | 151 | 170 | 213 | 68 | 134 | 129 | 192 | 70 | 19 | 120 | 172 | 82 | 38 | 54 | 78 | 104 | 110 | 111 | 127 | 62 | 87 | 147 | 111 | 128 | 71 | 161 | 383 | 107 | 208 | 186 | 134 | 229 | 260 | 139 |
| GCA | 276 | 129 | 55 | 159 |  | 52 | 62 | 96 | 37 | 70 | 166 | 58 | 74 | 66 |  | 56 | 115 | 115 | 82 | 110 | 48 | 78 | 92 | 62 | 22 | 60 | 30 | 54 | 31 | 43 | 36 | 45 | 98 | 106 | 109 | 156 | 19 | 94 | 65 | 47 | 42 | 13 | 83 | 85 | 44 | 35 | 81 | 50 | 41 | 50 | 102 | 124 | 57 | 63 | 61 | 98 | 65 | 48 | 68 | 189 | 118 | 119 | 106 | 40 | 86 | 123 | 90 |
| GCC | 190 | 55 | 33 | 67 |  | 40 | 54 | 16 | 6 | 28 | 91 | 31 | 65 | 41 |  | 28 | 32 | 52 | 26 | 56 | 10 | 16 | 31 | 31 | 10 | 21 | 18 | 23 | 4 | 10 | 21 | 20 | 36 | 29 | 45 | 34 | 14 | 44 | 36 | 37 | 8 | 12 | 12 | 25 | 26 | 4 | 13 | 17 | 23 | 15 | 19 | 53 | 25 | 67 | 14 | 37 | 10 | 18 | 52 | 75 | 42 | 65 | 39 | 27 | 63 | 61 | 43 |
| GCG | 113 | 39 | 34 | 45 |  | 24 | 37 | 25 | 27 | 27 | 65 | 29 | 16 | 14 |  | 21 | 10 | 38 | 5 | 56 | 17 | 42 | 29 | 28 | 6 | 6 | 20 | 15 | 6 | 7 | 9 | 12 | 14 | 29 | 7 | 43 | 6 | 8 | 11 | 9 | 8 | 2 | 29 | 35 | 44 | 5 | 33 | 2 | 8 | 23 | 53 | 21 | 60 | 25 | 11 | 41 | 28 | 20 | 24 | 37 | 58 | 26 | 26 | 10 | 39 | 62 | 21 |
| GCT | 551 | 138 | 107 | 188 |  | 88 | 156 | 41 | 62 | 117 | 231 | 140 | 122 | 120 |  | 60 | 139 | 161 | 123 | 199 | 106 | 80 | 108 | 85 | 46 | 30 | 60 | 49 | 30 | 47 | 30 | 99 | 74 | 92 | 122 | 96 | 70 | 111 | 76 | 111 | 46 | 7 | 84 | 75 | 94 | 82 | 139 | 65 | 77 | 54 | 94 | 87 | 67 | 117 | 81 | 176 | 96 | 45 | 103 | 183 | 184 | 154 | 108 | 114 | 151 | 154 | 79 |
| GGA | 489 | 129 | 69 | 140 |  | 75 | 58 | 24 | 33 | 84 | 177 | 103 | 180 | 122 |  | 80 | 92 | 128 | 120 | 110 | 30 | 130 | 87 | 101 | 53 | 39 | 16 | 35 | 15 | 48 | 50 | 87 | 45 | 90 | 116 | 100 | 56 | 68 | 76 | 73 | 32 | 16 | 58 | 112 | 52 | 95 | 51 | 41 | 88 | 94 | 126 | 115 | 39 | 62 | 92 | 133 | 68 | 27 | 110 | 115 | 116 | 224 | 103 | 47 | 108 | 148 | 90 |
| GGC | 335 | 61 | 31 | 76 |  | 25 | 58 | 47 | 5 | 48 | 119 | 48 | 61 | 39 |  | 44 | 20 | 60 | 57 | 60 | 47 | 35 | 40 | 27 | 11 | 19 | 17 | 14 | 7 | 36 | 7 | 37 | 32 | 62 | 23 | 37 | 19 | 27 | 38 | 48 | 12 | 5 | 51 | 78 | 11 | 22 | 23 | 3 | 7 | 22 | 32 | 40 | 9 | 20 | 10 | 66 | 61 | 22 | 59 | 111 | 21 | 87 | 36 | 48 | 26 | 59 | 38 |
| GGG | 159 | 71 | 76 | 36 |  | 54 | 25 | 47 | 31 | 45 | 99 | 28 | 71 | 46 |  | 47 | 30 | 55 | 37 | 76 | 20 | 73 | 26 | 13 | 27 | 17 | 16 | 30 | 2 | 17 | 13 | 52 | 25 | 96 | 34 | 31 | 23 | 56 | 51 | 59 | 20 | 6 | 36 | 80 | 20 | 30 | 42 | 18 | 27 | 34 | 38 | 37 | 22 | 36 | 24 | 49 | 45 | 14 | 27 | 86 | 52 | 91 | 39 | 34 | 43 | 116 | 42 |
| GGT | 580 | 120 | 51 | 163 |  | 42 | 87 | 48 | 47 | 88 | 177 | 140 | 198 | 85 |  | 79 | 103 | 148 | 50 | 146 | 44 | 96 | 82 | 31 | 43 | 29 | 34 | 52 | 11 | 104 | 18 | 51 | 50 | 87 | 125 | 95 | 8 | 75 | 94 | 51 | 41 | 44 | 63 | 51 | 65 | 64 | 63 | 24 | 53 | 97 | 95 | 76 | 37 | 71 | 70 | 77 | 98 | 44 | 73 | 153 | 78 | 238 | 146 | 108 | 73 | 81 | 121 |
| GTA | 232 | 126 | 25 | 67 |  | 20 | 46 | 47 | 17 | 34 | 90 | 71 | 44 | 65 |  | 34 | 35 | 43 | 35 | 63 | 26 | 27 | 42 | 44 | 18 | 8 | 32 | 13 | 26 | 40 | 13 | 35 | 47 | 31 | 70 | 56 | 37 | 56 | 34 | 43 | 37 | 16 | 29 | 101 | 24 | 42 | 21 | 16 | 31 | 36 | 37 | 53 | 16 | 28 | 45 | 46 | 41 | 8 | 45 | 79 | 49 | 59 | 64 | 36 | 47 | 78 | 51 |
| GTC | 157 | 62 | 24 | 62 |  | 39 | 21 | 28 | 21 | 30 | 87 | 47 | 26 | 93 |  | 40 | 44 | 70 | 25 | 70 | 10 | 32 | 39 | 30 | 17 | 10 | 8 | 27 | 10 | 19 | 7 | 34 | 22 | 63 | 35 | 61 | 18 | 42 | 48 | 22 | 6 | 3 | 23 | 59 | 40 | 22 | 26 | 26 | 22 | 38 | 26 | 95 | 31 | 50 | 37 | 67 | 25 | 24 | 43 | 89 | 32 | 46 | 98 | 44 | 57 | 68 | 49 |
| GTG | 222 | 102 | 74 | 108 |  | 63 | 68 | 64 | 25 | 46 | 158 | 31 | 70 | 76 |  | 57 | 94 | 73 | 41 | 66 | 41 | 54 | 63 | 63 | 4 | 14 | 7 | 67 | 40 | 30 | 18 | 12 | 100 | 112 | 31 | 113 | 37 | 49 | 74 | 55 | 13 | 41 | 49 | 60 | 32 | 29 | 78 | 48 | 45 | 21 | 56 | 105 | 32 | 41 | 68 | 101 | 68 | 47 | 36 | 100 | 45 | 110 | 124 | 63 | 61 | 163 | 99 |
| GTT | 492 | 168 | 59 | 172 |  | 103 | 159 | 54 | 32 | 74 | 197 | 147 | 122 | 154 |  | 70 | 107 | 181 | 97 | 230 | 78 | 79 | 91 | 69 | 42 | 54 | 40 | 32 | 23 | 38 | 61 | 89 | 122 | 150 | 128 | 122 | 37 | 69 | 68 | 101 | 48 | 40 | 115 | 144 | 28 | 64 | 102 | 73 | 26 | 100 | 135 | 113 | 42 | 78 | 85 | 108 | 66 | 83 | 146 | 176 | 105 | 186 | 214 | 72 | 159 | 148 | 151 |
| TAA | 28 | 9 | 9 | 8 |  | 2 | 10 | 1 | 1 | 2 | 5 | 2 | 3 | 6 |  | 10 | 4 | 6 |  | 5 |  | 7 |  |  |  | 3 | 1 |  | 2 |  | 2 | 4 | 3 | 2 | 2 | 17 |  | 9 | 1 | 4 | 1 | 5 | 3 | 4 | 4 | 1 |  | 6 | 4 | 4 | 2 | 14 | 2 | 2 | 1 | 2 |  |  | 1 | 1 | 6 |  | 1 |  | 6 | 4 | 8 |
| TAC | 353 | 95 | 49 | 61 |  | 39 | 71 | 68 | 41 | 66 | 73 | 41 | 69 | 51 |  | 84 | 42 | 47 | 89 | 87 | 54 | 74 | 36 | 14 | 24 | 24 | 25 | 16 | 22 | 23 | 14 | 35 | 41 | 38 | 64 | 89 | 33 | 42 | 38 | 48 | 24 | 16 | 43 | 68 | 22 | 32 | 24 | 28 | 28 | 53 | 52 | 47 | 42 | 28 | 47 | 51 | 61 | 68 | 38 | 94 | 51 | 105 | 70 | 55 | 58 | 103 | 51 |
| TAG | 45 | 2 | 10 | 4 |  | 8 | 1 | 6 | 5 | 2 | 3 | 9 |  |  |  | 4 | 2 |  |  |  | 5 |  |  | 3 |  | 1 |  | 4 |  |  | 6 |  | 2 | 2 |  |  | 2 |  | 2 | 7 |  |  | 2 | 1 | 1 |  |  |  |  |  | 3 | 4 | 2 |  | 4 | 1 | 5 | 1 |  | 18 |  | 2 |  | 2 |  | 10 | 4 |
| TAT | 224 | 34 | 19 | 97 |  | 32 | 56 | 39 | 35 | 76 | 152 | 61 | 39 | 82 |  | 62 | 133 | 68 | 78 | 89 | 64 | 82 | 48 | 44 | 22 | 20 | 25 | 24 | 18 | 13 | 12 | 46 | 83 | 86 | 91 | 115 | 30 | 70 | 63 | 28 | 14 | 19 | 40 | 60 | 52 | 19 | 29 | 71 | 19 | 66 | 59 | 23 | 28 | 41 | 28 | 57 | 66 | 13 | 62 | 116 | 67 | 111 | 66 | 48 | 93 | 154 | 117 |
| TCA | 284 | 147 | 82 | 124 |  | 72 | 57 | 64 | 34 | 79 | 210 | 148 | 93 | 52 |  | 79 | 109 | 91 | 75 | 101 | 61 | 83 | 117 | 29 | 28 | 8 | 64 | 55 | 27 | 53 | 12 | 52 | 112 | 127 | 93 | 132 | 41 | 54 | 121 | 101 | 33 | 13 | 26 | 79 | 60 | 42 | 63 | 61 | 33 | 65 | 45 | 99 | 44 | 104 | 51 | 50 | 32 | 53 | 65 | 191 | 138 | 132 | 59 | 57 | 159 | 100 | 104 |
| TCC | 205 | 76 | 24 | 52 |  | 21 | 44 | 17 | 18 | 32 | 74 | 56 | 31 | 42 |  | 80 | 33 | 65 | 80 | 81 | 23 | 47 | 50 | 18 | 24 | 13 | 8 | 29 | 10 | 37 | 5 | 41 | 18 | 45 | 31 | 55 | 15 | 27 | 27 | 53 | 21 | 5 | 19 | 41 | 20 | 9 | 11 | 25 | 33 | 22 | 24 | 35 | 38 | 4 | 21 | 32 | 27 | 16 | 61 | 60 | 27 | 68 | 42 | 41 | 75 | 68 | 63 |
| TCG | 115 | 63 | 30 | 42 |  | 14 | 14 | 14 | 7 | 10 | 37 | 17 | 35 | 34 |  | 18 | 23 | 27 | 31 | 34 | 65 | 47 | 41 | 8 | 11 | 11 | 14 | 17 | 8 | 6 | 16 | 10 | 16 | 29 | 23 | 36 | 8 | 44 | 25 | 19 | 14 | 5 | 23 | 52 | 28 | 11 | 25 | 8 | 23 | 25 | 47 | 10 | 20 | 24 | 27 | 36 | 26 | 19 | 25 | 49 | 44 | 35 | 26 | 14 | 40 | 48 | 32 |
| TCT | 312 | 132 | 65 | 177 |  | 82 | 163 | 70 | 37 | 83 | 214 | 108 | 119 | 81 |  | 98 | 200 | 143 | 90 | 162 | 56 | 84 | 149 | 60 | 44 | 34 | 44 | 55 | 28 | 37 | 52 | 76 | 112 | 115 | 128 | 90 | 65 | 79 | 114 | 77 | 31 | 18 | 53 | 135 | 61 | 58 | 79 | 45 | 116 | 98 | 78 | 134 | 50 | 97 | 102 | 103 | 89 | 61 | 67 | 222 | 94 | 157 | 83 | 79 | 163 | 135 | 86 |
| TGA | 63 | 6 | 6 | 13 |  | 3 | 4 | 4 |  | 7 | 4 | 4 | 1 | 1 |  | 3 | 16 |  | 2 | 8 |  |  |  | 5 | 10 |  | 7 |  | 4 | 2 | 4 | 4 | 8 | 4 | 8 | 5 |  | 2 | 7 | 8 | 2 | 1 | 5 | 2 | 5 | 2 | 3 | 4 | 1 | 14 | 8 | 4 | 4 |  | 4 | 10 | 5 |  | 1 | 2 | 4 | 2 | 4 | 4 | 11 | 7 | 8 |
| TGC | 140 | 28 | 17 | 89 |  | 17 | 20 | 9 | 8 | 20 | 48 | 29 | 12 | 28 |  | 36 | 25 | 36 | 15 | 35 | 16 | 15 | 18 | 38 | 4 | 22 | 21 | 32 | 6 | 6 | 10 | 11 | 34 | 22 | 42 | 36 | 4 | 24 | 46 | 15 | 6 | 2 | 23 | 33 | 12 | 2 | 2 | 6 | 8 | 37 | 22 | 8 | 19 | 44 | 39 | 24 | 16 | 15 | 34 | 36 | 16 | 30 | 30 | 29 | 38 | 63 | 49 |
| TGG | 167 | 84 | 52 | 90 |  | 47 | 25 | 22 | 31 | 15 | 61 | 50 | 41 | 40 |  | 56 | 88 | 143 | 40 | 45 | 41 | 54 | 38 | 36 | 14 | 6 | 28 | 44 | 13 | 27 | 14 | 25 | 37 | 60 | 54 | 84 | 20 | 73 | 18 | 32 | 26 | 8 | 16 | 52 | 57 | 23 | 25 | 14 | 19 | 34 | 44 | 45 | 17 | 38 | 33 | 36 | 45 | 14 | 76 | 79 | 33 | 102 | 50 | 43 | 59 | 85 | 51 |
| TGT | 177 | 37 | 46 | 87 |  | 29 | 21 | 3 | 17 | 46 | 72 | 22 | 18 | 42 |  | 43 | 57 | 67 | 35 | 41 | 29 | 36 | 15 | 10 | 31 | 6 | 20 | 18 | 8 | 24 | 10 | 41 | 34 | 32 | 34 | 34 | 24 | 14 | 47 | 36 | 7 | 7 | 23 | 32 | 37 | 19 | 19 | 12 | 29 | 32 | 57 | 33 | 8 | 48 | 25 | 39 | 48 | 17 | 29 | 87 | 49 | 70 | 84 | 38 | 45 | 52 | 44 |
| TTA | 303 | 82 | 64 | 41 |  | 54 | 72 | 30 | 44 | 41 | 83 | 50 | 59 | 48 |  | 25 | 71 | 64 | 80 | 68 | 37 | 47 | 42 | 47 | 12 | 13 | 24 | 44 | 22 | 46 | 39 | 18 | 32 | 34 | 72 | 89 | 32 | 17 | 42 | 50 | 38 | 9 | 64 | 98 | 10 | 11 | 43 | 6 | 33 | 19 | 68 | 58 | 22 | 35 | 41 | 56 | 51 | 39 | 32 | 119 | 22 | 70 | 57 | 40 | 69 | 100 | 98 |
| TTC | 274 | 71 | 46 | 156 |  | 49 | 39 | 37 | 17 | 73 | 155 | 36 | 63 | 87 |  | 58 | 29 | 119 | 73 | 80 | 28 | 60 | 53 | 46 | 20 | 36 | 51 | 37 | 17 | 27 | 11 | 53 | 42 | 84 | 103 | 91 | 45 | 68 | 45 | 87 | 37 | 44 | 43 | 68 | 53 | 29 | 45 | 22 | 58 | 22 | 106 | 80 | 51 | 52 | 58 | 81 | 54 | 47 | 70 | 102 | 72 | 81 | 82 | 64 | 75 | 77 | 114 |
| TTG | 494 | 115 | 115 | 161 |  | 76 | 105 | 48 | 30 | 61 | 180 | 77 | 94 | 88 |  | 70 | 96 | 108 | 130 | 131 | 51 | 93 | 99 | 92 | 52 | 26 | 36 | 48 | 45 | 22 | 37 | 57 | 105 | 89 | 105 | 141 | 88 | 44 | 74 | 77 | 49 | 28 | 40 | 58 | 48 | 44 | 57 | 55 | 50 | 19 | 105 | 52 | 53 | 79 | 83 | 128 | 42 | 28 | 86 | 145 | 75 | 120 | 87 | 83 | 71 | 166 | 123 |
| TTT | 530 | 124 | 85 | 114 |  | 92 | 59 | 53 | 16 | 101 | 197 | 77 | 111 | 71 | 1 | 75 | 55 | 145 | 115 | 135 | 93 | 141 | 78 | 115 | 44 | 38 | 50 | 46 | 27 | 30 | 33 | 46 | 105 | 116 | 128 | 153 | 58 | 113 | 99 | 84 | 51 | 48 | 52 | 97 | 82 | 66 | 73 | 78 | 77 | 54 | 76 | 85 | 36 | 44 | 94 | 138 | 81 | 61 | 90 | 170 | 88 | 137 | 86 | 89 | 107 | 106 | 161 |


---


 **Amino acid changes**

How to read this table:   
- Rows are reference amino acids and columns are changed amino acids. E.g. Row 'A' column 'E' indicates how many 'A' amino acids have been replaced by 'E' amino acids.  
- Red background colors indicate that more changes happened (heat-map).  
- Diagonals are indicated using grey background color   
- WARNING: This table may include different translation codon tables (e.g. mamalian DNA and mitochondrial DNA).

|  | \* | - | ? | A | C | D | E | F | G | H | I | K | L | M | N | P | Q | R | S | T | V | W | Y |
| --- | --- | --- | --- | --- | --- | --- | --- | --- | --- | --- | --- | --- | --- | --- | --- | --- | --- | --- | --- | --- | --- | --- | --- |
| \* | 45 | 136 |  | 38 | 15 | 12 | 33 | 37 | 23 | 5 | 41 | 42 | 66 | 6 | 38 | 30 | 20 | 40 | 48 | 43 | 46 | 4 | 17 |
| - | 662 |  | 556 | 703 | 437 | 295 | 573 | 469 | 1,247 | 412 | 504 | 749 | 1,480 | 216 | 419 | 531 | 548 | 1,043 | 1,307 | 767 | 833 | 345 | 311 |
| ? |  | 6 |  |  |  |  |  |  |  |  |  |  | 2 |  |  |  |  |  | 5 | 1 |  |  |  |
| A | 1,041 | 1,128 | 2 | 550 | 681 | 297 | 586 | 572 | 820 | 441 | 697 | 820 | 1,528 | 366 | 433 | 535 | 637 | 1,245 | 1,428 | 861 | 693 | 364 | 376 |
| C | 256 | 317 |  | 119 | 179 | 121 | 108 | 176 | 156 | 78 | 211 | 241 | 432 | 103 | 109 | 152 | 127 | 264 | 343 | 144 | 203 | 100 | 91 |
| D | 873 | 1,108 |  | 559 | 406 | 286 | 496 | 529 | 575 | 402 | 585 | 630 | 1,496 | 292 | 365 | 485 | 554 | 1,252 | 1,121 | 683 | 603 | 311 | 298 |
| E | 1,078 | 909 |  | 575 | 559 | 424 | 566 | 554 | 823 | 421 | 698 | 860 | 1,610 | 339 | 547 | 425 | 678 | 1,411 | 1,507 | 775 | 721 | 427 | 480 |
| F | 533 | 803 | 2 | 446 | 328 | 247 | 425 | 457 | 513 | 252 | 405 | 465 | 1,013 | 264 | 272 | 400 | 416 | 754 | 893 | 395 | 493 | 218 | 239 |
| G | 922 | 1,558 | 5 | 615 | 591 | 365 | 489 | 541 | 859 | 376 | 759 | 796 | 1,653 | 391 | 423 | 493 | 726 | 1,541 | 1,584 | 775 | 799 | 640 | 303 |
| H | 308 | 459 | 1 | 208 | 229 | 138 | 234 | 244 | 345 | 189 | 235 | 271 | 622 | 83 | 168 | 256 | 283 | 454 | 555 | 353 | 238 | 168 | 123 |
| I | 792 | 862 | 2 | 428 | 438 | 313 | 452 | 484 | 552 | 405 | 570 | 579 | 1,198 | 352 | 489 | 317 | 495 | 1,013 | 1,115 | 505 | 538 | 291 | 424 |
| K | 1,122 | 2,022 | 3 | 535 | 538 | 302 | 693 | 581 | 853 | 458 | 770 | 1,079 | 1,811 | 366 | 603 | 474 | 741 | 1,461 | 1,376 | 897 | 744 | 416 | 350 |
| L | 1,343 | 1,852 | 4 | 950 | 846 | 531 | 811 | 918 | 904 | 534 | 1,052 | 1,043 | 2,334 | 527 | 770 | 747 | 864 | 1,977 | 2,181 | 1,171 | 1,050 | 531 | 630 |
| M | 398 | 631 | 5 | 231 | 175 | 170 | 258 | 75 | 282 | 201 | 271 | 283 | 596 | 103 | 163 | 203 | 331 | 519 | 501 | 256 | 250 | 126 | 112 |
| N | 629 | 658 | 4 | 325 | 387 | 298 | 501 | 502 | 508 | 334 | 483 | 640 | 1,068 | 220 | 303 | 346 | 490 | 817 | 951 | 498 | 577 | 236 | 284 |
| P | 696 | 982 | 5 | 460 | 436 | 291 | 448 | 413 | 528 | 321 | 541 | 703 | 1,111 | 339 | 431 | 421 | 524 | 941 | 1,061 | 708 | 434 | 356 | 262 |
| Q | 561 | 414 | 1 | 330 | 269 | 218 | 388 | 441 | 311 | 232 | 426 | 371 | 862 | 187 | 360 | 341 | 382 | 712 | 864 | 438 | 496 | 220 | 130 |
| R | 846 | 949 |  | 471 | 569 | 236 | 535 | 512 | 654 | 343 | 559 | 743 | 1,345 | 288 | 452 | 412 | 541 | 963 | 1,169 | 627 | 486 | 314 | 325 |
| S | 1,462 | 1,517 | 5 | 786 | 729 | 586 | 850 | 1,075 | 1,088 | 686 | 1,247 | 1,206 | 2,269 | 466 | 608 | 623 | 928 | 1,797 | 1,834 | 1,044 | 1,082 | 558 | 503 |
| T | 700 | 1,177 | 1 | 395 | 487 | 336 | 513 | 512 | 547 | 507 | 542 | 628 | 1,231 | 314 | 440 | 396 | 600 | 1,049 | 1,141 | 662 | 634 | 333 | 401 |
| V | 1,007 | 1,102 | 1 | 641 | 731 | 353 | 568 | 674 | 872 | 390 | 679 | 867 | 1,753 | 367 | 407 | 460 | 621 | 1,258 | 1,638 | 766 | 736 | 401 | 356 |
| W | 162 | 167 |  | 82 | 83 | 38 | 157 | 110 | 157 | 79 | 184 | 174 | 304 | 143 | 99 | 84 | 99 | 200 | 261 | 93 | 111 | 102 | 50 |
| Y | 349 | 577 |  | 232 | 254 | 164 | 316 | 319 | 306 | 202 | 488 | 287 | 844 | 115 | 139 | 198 | 332 | 475 | 651 | 452 | 376 | 216 | 145 |


---


 **Variants by chromosome**

```
		  

		1, Position,0,1000000,2000000,3000000,4000000,5000000,6000000,7000000,8000000,9000000,10000000,11000000,12000000,13000000,14000000,15000000,16000000,17000000,18000000,19000000,20000000,21000000,22000000,23000000,24000000,25000000,26000000,27000000,28000000,29000000,30000000
1,Count,6,5,10,12,11,7,4,11,8,7,7,10,3,6,10,23,5,13,5,14,10,13,9,5,11,6,9,7,7,9,3

	
```

```
		  

		2, Position,0,100000,200000,300000,400000,500000,600000,700000,800000,900000,1000000,1100000,1200000,1300000,1400000,1500000,1600000,1700000,1800000,1900000,2000000,2100000,2200000,2300000,2400000,2500000,2600000,2700000,2800000,2900000,3000000,3100000,3200000,3300000,3400000,3500000,3600000,3700000,3800000,3900000,4000000,4100000,4200000,4300000,4400000,4500000,4600000,4700000,4800000,4900000,5000000,5100000,5200000,5300000,5400000,5500000,5600000,5700000,5800000,5900000,6000000,6100000,6200000,6300000,6400000,6500000,6600000,6700000,6800000,6900000,7000000,7100000,7200000,7300000,7400000,7500000,7600000,7700000,7800000,7900000,8000000,8100000,8200000,8300000,8400000,8500000,8600000,8700000,8800000,8900000,9000000,9100000,9200000,9300000,9400000,9500000,9600000,9700000,9800000,9900000,10000000,10100000,10200000,10300000,10400000,10500000,10600000,10700000,10800000,10900000,11000000,11100000,11200000,11300000,11400000,11500000,11600000,11700000,11800000,11900000,12000000,12100000,12200000,12300000,12400000,12500000,12600000,12700000,12800000,12900000,13000000,13100000,13200000,13300000,13400000,13500000,13600000,13700000,13800000,13900000,14000000,14100000,14200000,14300000,14400000,14500000,14600000,14700000,14800000,14900000,15000000,15100000,15200000,15300000,15400000,15500000,15600000,15700000,15800000,15900000,16000000,16100000,16200000,16300000,16400000,16500000,16600000,16700000,16800000,16900000,17000000,17100000,17200000,17300000,17400000,17500000,17600000,17700000,17800000,17900000,18000000,18100000,18200000,18300000,18400000,18500000,18600000,18700000,18800000,18900000,19000000,19100000,19200000,19300000,19400000,19500000,19600000
2,Count,5,0,0,1,1,0,0,1,2,0,0,2,2,0,0,0,2,1,1,1,0,2,0,0,0,0,0,0,0,1,4,1,1,0,0,1,37,0,0,0,0,2,0,3,0,1,1,1,1,0,1,1,1,1,0,0,0,0,1,1,0,1,0,1,1,0,1,1,0,0,0,0,1,1,1,0,1,0,0,0,1,0,1,2,0,1,2,1,0,0,4,1,1,0,0,0,0,2,1,1,0,1,0,3,2,1,1,1,0,0,0,0,0,0,0,1,1,0,1,2,0,0,0,0,0,2,1,0,0,11,1,1,2,0,0,2,2,0,1,2,0,1,1,0,1,1,0,1,3,0,2,4,1,0,0,3,1,0,0,0,0,1,1,2,0,1,0,1,0,1,2,2,0,1,1,0,1,1,1,1,0,1,0,1,2,1,1,0,0,2,0,0,1,0,2,2,2

	
```

```
		  

		3, Position,0,100000,200000,300000,400000,500000,600000,700000,800000,900000,1000000,1100000,1200000,1300000,1400000,1500000,1600000,1700000,1800000,1900000,2000000,2100000,2200000,2300000,2400000,2500000,2600000,2700000,2800000,2900000,3000000,3100000,3200000,3300000,3400000,3500000,3600000,3700000,3800000,3900000,4000000,4100000,4200000,4300000,4400000,4500000,4600000,4700000,4800000,4900000,5000000,5100000,5200000,5300000,5400000,5500000,5600000,5700000,5800000,5900000,6000000,6100000,6200000,6300000,6400000,6500000,6600000,6700000,6800000,6900000,7000000,7100000,7200000,7300000,7400000,7500000,7600000,7700000,7800000,7900000,8000000,8100000,8200000,8300000,8400000,8500000,8600000,8700000,8800000,8900000,9000000,9100000,9200000,9300000,9400000,9500000,9600000,9700000,9800000,9900000,10000000,10100000,10200000,10300000,10400000,10500000,10600000,10700000,10800000,10900000,11000000,11100000,11200000,11300000,11400000,11500000,11600000,11700000,11800000,11900000,12000000,12100000,12200000,12300000,12400000,12500000,12600000,12700000,12800000,12900000,13000000,13100000,13200000,13300000,13400000,13500000,13600000,13700000,13800000,13900000,14000000,14100000,14200000,14300000,14400000,14500000,14600000,14700000,14800000,14900000,15000000,15100000,15200000,15300000,15400000,15500000,15600000,15700000,15800000,15900000,16000000,16100000,16200000,16300000,16400000,16500000,16600000,16700000,16800000,16900000,17000000,17100000,17200000,17300000,17400000,17500000,17600000,17700000,17800000,17900000,18000000,18100000,18200000,18300000,18400000,18500000,18600000,18700000,18800000,18900000,19000000,19100000,19200000,19300000,19400000,19500000,19600000,19700000,19800000,19900000,20000000,20100000,20200000,20300000,20400000,20500000,20600000,20700000,20800000,20900000,21000000,21100000,21200000,21300000,21400000,21500000,21600000,21700000,21800000,21900000,22000000,22100000,22200000,22300000,22400000,22500000,22600000,22700000,22800000,22900000,23000000,23100000,23200000,23300000,23400000
3,Count,2,3,0,4,0,0,1,0,1,0,1,1,0,0,1,0,0,2,1,2,2,2,1,0,1,2,1,0,3,1,4,1,0,0,1,0,1,1,1,0,0,1,2,1,0,1,3,0,0,1,0,0,3,0,0,2,1,0,1,1,0,1,0,1,1,1,1,0,0,1,0,2,2,1,2,0,2,0,0,1,1,0,1,1,0,0,0,1,0,1,0,2,1,1,0,2,0,1,0,0,0,1,1,1,2,0,0,2,1,0,3,2,2,0,1,0,0,0,1,0,0,0,0,0,2,0,0,0,1,0,1,1,0,1,0,5,1,2,4,2,1,8,9,0,1,0,0,1,2,2,1,2,1,2,0,0,1,0,0,0,0,2,2,1,0,1,2,1,1,0,1,0,1,0,0,2,0,3,2,0,1,0,1,0,0,3,0,1,1,1,0,1,0,0,3,0,1,0,0,0,0,3,0,1,0,1,0,3,1,2,1,2,0,0,1,3,1,0,1,0,3,0,1,3,0,0,3,0,1,1,1,0,0,2,0

	
```

```
		  

		4, Position,0,100000,200000,300000,400000,500000,600000,700000,800000,900000,1000000,1100000,1200000,1300000,1400000,1500000,1600000,1700000,1800000,1900000,2000000,2100000,2200000,2300000,2400000,2500000,2600000,2700000,2800000,2900000,3000000,3100000,3200000,3300000,3400000,3500000,3600000,3700000,3800000,3900000,4000000,4100000,4200000,4300000,4400000,4500000,4600000,4700000,4800000,4900000,5000000,5100000,5200000,5300000,5400000,5500000,5600000,5700000,5800000,5900000,6000000,6100000,6200000,6300000,6400000,6500000,6600000,6700000,6800000,6900000,7000000,7100000,7200000,7300000,7400000,7500000,7600000,7700000,7800000,7900000,8000000,8100000,8200000,8300000,8400000,8500000,8600000,8700000,8800000,8900000,9000000,9100000,9200000,9300000,9400000,9500000,9600000,9700000,9800000,9900000,10000000,10100000,10200000,10300000,10400000,10500000,10600000,10700000,10800000,10900000,11000000,11100000,11200000,11300000,11400000,11500000,11600000,11700000,11800000,11900000,12000000,12100000,12200000,12300000,12400000,12500000,12600000,12700000,12800000,12900000,13000000,13100000,13200000,13300000,13400000,13500000,13600000,13700000,13800000,13900000,14000000,14100000,14200000,14300000,14400000,14500000,14600000,14700000,14800000,14900000,15000000,15100000,15200000,15300000,15400000,15500000,15600000,15700000,15800000,15900000,16000000,16100000,16200000,16300000,16400000,16500000,16600000,16700000,16800000,16900000,17000000,17100000,17200000,17300000,17400000,17500000,17600000,17700000,17800000,17900000,18000000,18100000,18200000,18300000,18400000,18500000
4,Count,2,2,2,0,1,1,1,0,3,0,0,1,1,0,0,0,0,0,0,1,2,0,3,3,0,0,2,3,1,0,5,0,1,1,1,0,2,0,0,36,12,1,1,0,0,2,1,0,0,2,1,0,0,2,0,0,1,0,1,0,0,0,1,0,1,0,2,1,0,1,0,1,1,2,2,1,0,0,1,4,0,0,0,1,0,0,1,2,1,0,1,2,1,4,1,0,0,3,1,1,0,0,3,0,3,0,0,0,0,1,0,1,2,0,1,0,0,2,1,1,0,0,1,1,1,0,2,1,0,0,0,0,1,1,0,1,0,2,0,0,0,0,2,1,0,0,2,1,4,0,1,0,1,1,1,1,1,0,0,1,0,0,0,1,1,1,0,0,3,0,0,1,1,0,0,0,1,1,0,0,1,0,0,1,1,2

	
```

```
		  

		5, Position,0,100000,200000,300000,400000,500000,600000,700000,800000,900000,1000000,1100000,1200000,1300000,1400000,1500000,1600000,1700000,1800000,1900000,2000000,2100000,2200000,2300000,2400000,2500000,2600000,2700000,2800000,2900000,3000000,3100000,3200000,3300000,3400000,3500000,3600000,3700000,3800000,3900000,4000000,4100000,4200000,4300000,4400000,4500000,4600000,4700000,4800000,4900000,5000000,5100000,5200000,5300000,5400000,5500000,5600000,5700000,5800000,5900000,6000000,6100000,6200000,6300000,6400000,6500000,6600000,6700000,6800000,6900000,7000000,7100000,7200000,7300000,7400000,7500000,7600000,7700000,7800000,7900000,8000000,8100000,8200000,8300000,8400000,8500000,8600000,8700000,8800000,8900000,9000000,9100000,9200000,9300000,9400000,9500000,9600000,9700000,9800000,9900000,10000000,10100000,10200000,10300000,10400000,10500000,10600000,10700000,10800000,10900000,11000000,11100000,11200000,11300000,11400000,11500000,11600000,11700000,11800000,11900000,12000000,12100000,12200000,12300000,12400000,12500000,12600000,12700000,12800000,12900000,13000000,13100000,13200000,13300000,13400000,13500000,13600000,13700000,13800000,13900000,14000000,14100000,14200000,14300000,14400000,14500000,14600000,14700000,14800000,14900000,15000000,15100000,15200000,15300000,15400000,15500000,15600000,15700000,15800000,15900000,16000000,16100000,16200000,16300000,16400000,16500000,16600000,16700000,16800000,16900000,17000000,17100000,17200000,17300000,17400000,17500000,17600000,17700000,17800000,17900000,18000000,18100000,18200000,18300000,18400000,18500000,18600000,18700000,18800000,18900000,19000000,19100000,19200000,19300000,19400000,19500000,19600000,19700000,19800000,19900000,20000000,20100000,20200000,20300000,20400000,20500000,20600000,20700000,20800000,20900000,21000000,21100000,21200000,21300000,21400000,21500000,21600000,21700000,21800000,21900000,22000000,22100000,22200000,22300000,22400000,22500000,22600000,22700000,22800000,22900000,23000000,23100000,23200000,23300000,23400000,23500000,23600000,23700000,23800000,23900000,24000000,24100000,24200000,24300000,24400000,24500000,24600000,24700000,24800000,24900000,25000000,25100000,25200000,25300000,25400000,25500000,25600000,25700000,25800000,25900000,26000000,26100000,26200000,26300000,26400000,26500000,26600000,26700000,26800000,26900000
5,Count,0,0,1,1,2,0,0,0,0,0,1,0,0,1,1,1,0,0,0,0,1,1,0,1,1,0,0,0,0,0,0,0,2,1,1,1,0,1,1,1,1,0,2,2,3,1,0,0,0,0,0,1,0,2,1,1,1,0,1,0,3,0,0,2,1,0,1,1,1,0,0,2,0,3,1,0,1,2,0,0,0,0,1,1,1,0,1,0,0,1,1,1,0,0,2,0,0,0,0,0,1,0,0,0,0,0,1,0,1,0,1,5,0,0,0,0,1,46,1,1,0,1,2,1,0,1,1,0,7,1,1,2,2,0,2,0,1,0,1,0,0,0,0,1,0,0,0,1,1,2,1,1,2,0,2,1,0,0,0,1,1,0,1,0,2,1,0,2,0,0,1,0,2,0,1,1,2,0,0,1,1,1,2,1,0,0,1,0,1,1,0,1,2,1,1,1,1,0,1,2,2,1,3,0,2,0,0,1,0,0,1,3,0,1,1,2,1,0,0,0,0,2,0,0,0,1,1,2,1,0,2,1,1,1,3,0,1,1,2,0,0,2,0,0,0,1,0,2,0,0,0,0,1,2,2,0,0,1,2,3,1,0,2,1,0,1,1,2,0,1

	
```

```
		  

		chloroplast, Position,0,1
chloroplast,Count,0,0

	
```

```
		  

		mitochondria, Position,0,1
mitochondria,Count,0,0

	
```


---

 **Details by gene** 

**Here** you can find a tab-separated table.
