# Supplementary material for: Genomic impact of stress-induced transposable element mobility in Arabidopsis
Source: Nucleic Acids Res. 2021 Sep 22;49(18):10431–47. doi: 10.1093/nar/gkab828 (PMC8501995; doi:10.1093/nar/gkab828)
Supplement: gkab828_Supplemental_Files [file gkab828_supplemental_files.zip › Supplementary File 5 - hclines SNP INDELS CNV.zip!/snpEff_hclines_private_SNP.html]

### SnpEff: Variant analysis

**Contents**
Summary  
 Variant rate by chromosome  
Variants by type  
 Number of variants by impact   
 Number of variants by functional class   
 Number of variants by effect   
Quality histogram  
InDel length histogram  
Base variant table  
Transition vs transversions (ts/tv)  
 Allele frequency   
 Allele Count   
 Codon change table   
 Amino acid change table   
 Chromosome variants plots   
 Details by gene


---


**Summary**

|  |  |
| --- | --- |
| **Genome** | Arabidopsis\_thaliana |
| **Date** | 2021-06-28 15:13 |
| **SnpEff version** | ``` SnpEff 5.0e (build 2021-03-09 06:01), by Pablo Cingolani ``` |
| **Command line arguments** | ``` SnpEff  Arabidopsis_thaliana hclines_private_SNP.vcf ``` |
| **Warnings** | 43 |
| **Errors** | 0 |
| **Number of lines (input file)** | 2,150 |
| **Number of variants (before filter)** | 2,150 |
| **Number of not variants  (i.e. reference equals alternative)** | 0 |
| **Number of variants processed   (i.e. after filter and non-variants)** | 2,095 |
| **Number of known variants  (i.e. non-empty ID)** | 0 ( 0% ) |
| **Number of multi-allelic VCF entries  (i.e. more than two alleles)** | 0 |
| **Number of effects** | 5,198 |
| **Genome total length** | 119,667,805 |
| **Genome effective length** | 119,146,348 |
| **Variant rate** | 1 variant every 56,871 bases |


---


 **Variants rate details** 

| Chromosome | Length | Variants | Variants rate |
| --- | --- | --- | --- |
| 1 | 30,427,671 | 436 | 69,788 |
| 2 | 19,698,289 | 278 | 70,857 |
| 3 | 23,459,830 | 628 | 37,356 |
| 4 | 18,585,056 | 371 | 50,094 |
| 5 | 26,975,502 | 382 | 70,616 |
| Total | 119,146,348 | 2,095 | 56,871 |


---


 **Number variants by type**

| **Type** | **Total** |
| --- | --- |
| **SNP** | 2,095 |
| **MNP** | 0 |
| **INS** | 0 |
| **DEL** | 0 |
| **MIXED** | 0 |
| **INV** | 0 |
| **DUP** | 0 |
| **BND** | 0 |
| **INTERVAL** | 0 |
| **Total** | 2,095 |
| --- | --- |


---


 **Number of effects by impact** 

| **Type (alphabetical order)** |  | Count | Percent |
| --- | --- | --- | --- |
| **HIGH** |  | 29 | 0.558% |
| **LOW** |  | 107 | 2.058% |
| **MODERATE** |  | 188 | 3.617% |
| **MODIFIER** |  | 4,874 | 93.767% |


---


 **Number of effects by functional class** 

| **Type (alphabetical order)** |  | Count | Percent |
| --- | --- | --- | --- |
| **MISSENSE** |  | 188 | 63.3% |
| **NONSENSE** |  | 17 | 5.724% |
| **SILENT** |  | 92 | 30.976% |

  

Missense / Silent ratio: 2.0435


---


 **Number of effects by type and region** 

| Type | Region |
| --- | --- |
| **Type (alphabetical order)** \| \| Count \| Percent; **3\_prime\_UTR\_variant** \| \| 40 \| 0.765%; **5\_prime\_UTR\_premature\_start\_codon\_gain\_variant** \| \| 3 \| 0.057%; **5\_prime\_UTR\_variant** \| \| 21 \| 0.402%; **downstream\_gene\_variant** \| \| 1,464 \| 27.992%; **intergenic\_region** \| \| 1,759 \| 33.633%; **intron\_variant** \| \| 165 \| 3.155%; **missense\_variant** \| \| 188 \| 3.595%; **non\_coding\_transcript\_exon\_variant** \| \| 10 \| 0.191%; **splice\_acceptor\_variant** \| \| 4 \| 0.076%; **splice\_donor\_variant** \| \| 8 \| 0.153%; **splice\_region\_variant** \| \| 23 \| 0.44%; **stop\_gained** \| \| 17 \| 0.325%; **synonymous\_variant** \| \| 92 \| 1.759%; **upstream\_gene\_variant** \| \| 1,436 \| 27.457% | **Type (alphabetical order)** \| \| Count \| Percent; **DOWNSTREAM** \| \| 1,464 \| 28.165%; **EXON** \| \| 305 \| 5.868%; **INTERGENIC** \| \| 1,759 \| 33.84%; **INTRON** \| \| 145 \| 2.79%; **SPLICE\_SITE\_ACCEPTOR** \| \| 4 \| 0.077%; **SPLICE\_SITE\_DONOR** \| \| 8 \| 0.154%; **SPLICE\_SITE\_REGION** \| \| 13 \| 0.25%; **UPSTREAM** \| \| 1,436 \| 27.626%; **UTR\_3\_PRIME** \| \| 40 \| 0.77%; **UTR\_5\_PRIME** \| \| 24 \| 0.462% |


---


 **Quality:**

```
|  |  |
| --- | --- |
| Min | 35 |
| Max | 1,538 |
| Mean | 241.173 |
| Median | 160 |
| Standard deviation | 208.166 |
| Values | 35,38,40,42,43,45,49,50,51,52,53,54,55,56,57,58,59,60,61,62,63,64,65,66,67,68,69,70,71,72,73,74,75,76,77,78,79,80,81,82,83,84,85,86,87,88,89,90,91,92,93,94,95,96,97,98,99,100,101,102,103,104,105,106,107,108,109,110,111,112,113,114,115,116,117,118,119,120,121,122,123,124,125,126,127,128,129,130,131,132,133,134,135,136,137,138,139,140,141,142,143,144,145,146,147,148,149,150,151,152,153,154,155,156,157,158,159,160,161,162,163,164,165,166,167,168,169,170,171,172,173,174,175,176,177,178,179,180,181,182,183,184,185,186,187,188,189,190,191,192,193,194,195,196,197,198,199,200,202,203,204,205,206,207,208,209,210,211,212,214,215,216,217,218,219,220,222,223,225,226,227,229,230,231,232,234,235,236,237,238,239,240,241,242,243,244,246,247,248,249,250,251,252,253,254,255,256,258,259,260,261,262,263,264,265,266,267,268,269,270,271,273,274,275,276,277,278,279,280,281,282,283,284,285,286,287,288,289,290,292,294,295,296,297,298,299,301,302,303,304,305,307,308,309,310,312,313,316,318,319,320,322,323,325,327,328,329,331,333,335,336,337,338,339,340,343,345,346,347,349,351,353,355,356,357,358,360,361,363,364,365,366,367,369,370,371,372,373,375,376,377,379,381,384,385,386,388,391,394,396,397,398,399,400,402,403,406,407,408,409,411,412,413,415,417,418,420,421,424,425,427,430,434,435,436,438,439,442,443,447,450,456,457,463,464,465,467,469,470,472,474,475,476,477,479,481,483,484,485,488,489,493,494,495,497,498,501,502,503,504,506,508,513,515,516,517,518,519,520,522,523,524,526,527,528,529,531,532,534,535,537,538,540,543,544,546,552,553,554,558,561,563,564,565,568,571,574,577,578,580,581,582,585,587,588,592,594,595,596,597,598,603,604,605,606,607,609,612,615,617,618,619,621,622,625,627,628,630,634,637,641,643,645,648,651,653,655,656,657,658,660,661,662,663,664,665,667,672,674,682,684,686,693,697,700,707,709,710,713,714,716,719,721,722,723,725,727,730,732,734,739,740,741,745,747,750,752,754,757,760,761,762,764,765,767,769,777,778,779,784,785,787,788,792,797,805,811,812,814,815,818,822,827,831,839,845,856,862,865,890,893,895,898,902,917,918,921,922,923,925,927,937,943,946,967,970,976,982,987,989,994,1001,1002,1027,1049,1088,1116,1117,1145,1168,1187,1216,1250,1255,1272,1392,1538 |
| Count | 1,1,1,2,2,3,2,2,1,2,5,6,1,4,3,3,4,9,3,11,9,8,9,6,13,9,3,4,3,6,7,4,6,11,6,4,6,3,9,9,4,4,9,8,3,15,9,4,17,5,7,22,9,7,24,3,3,17,5,1,30,13,4,28,10,6,19,4,4,29,6,3,25,8,4,29,8,7,24,8,4,18,2,3,23,7,8,19,8,7,35,4,7,26,5,4,14,4,7,35,3,3,18,4,3,26,3,2,20,3,7,26,3,4,16,4,4,27,3,6,16,4,2,14,5,8,20,8,1,9,1,3,14,1,2,14,5,6,11,2,5,11,2,5,12,6,2,14,1,6,9,1,3,16,2,7,11,4,7,1,1,13,5,2,8,2,5,8,1,9,2,3,7,5,4,5,1,5,3,5,1,15,1,1,9,1,5,1,5,2,1,1,5,1,3,1,2,5,3,2,2,1,1,5,1,2,9,1,9,2,1,3,1,3,2,3,1,9,1,1,5,1,3,2,1,2,2,1,2,1,1,3,1,2,2,3,1,3,2,4,1,2,1,1,3,3,5,1,1,1,3,2,4,2,4,1,1,1,3,5,1,5,1,3,4,2,1,1,2,2,1,3,1,1,1,2,2,2,2,1,2,3,3,1,3,6,1,1,1,2,2,1,4,2,3,3,1,1,2,2,1,1,1,1,1,3,3,2,1,2,3,1,1,3,2,3,1,1,1,3,2,3,1,1,1,1,1,4,1,1,1,1,1,1,1,1,3,1,4,2,1,1,2,1,2,3,1,1,1,2,1,1,1,3,2,3,1,3,2,1,2,1,2,1,1,1,1,2,3,1,1,1,2,1,3,1,2,1,1,1,1,1,1,1,1,4,2,2,1,1,1,2,1,1,3,1,1,1,2,1,1,1,1,1,1,6,4,2,2,1,2,2,1,1,1,1,1,1,1,1,3,1,1,2,1,1,2,1,1,1,1,1,1,2,1,1,2,1,1,1,1,1,1,2,2,3,1,2,2,1,1,1,1,2,1,1,2,1,2,2,1,1,1,3,1,1,3,1,1,1,1,1,1,1,3,1,2,1,1,1,2,1,1,1,1,1,1,1,2,1,1,1,2,1,2,2,1,2,2,3,1,1,1,1,1,1,1,1,1,1,1,2,2,2,1,1,2,2,1,1,1,2,1,1,1,1,1,1,1,1,1,1,1,1,1,1,1,2,1,1,1,1,1,1,1,2,1,1,1,1,1,1,1,1,1,1,1 |
```


---


 **Insertions and deletions length:**

---


 **Base changes (SNPs)** 

|  |  |  |  |  |
| --- | --- | --- | --- | --- |
|  | **A** | **C** | **G** | **T** |
| **A** | 0 | 101 | 205 | 126 |
| **C** | 118 | 0 | 116 | 379 |
| **G** | 388 | 99 | 0 | 116 |
| **T** | 143 | 199 | 105 | 0 |

---


  **Ts/Tv (transitions / transversions)** 


**Note:** Only SNPs are used for this statistic.  
**Note:** This Ts/Tv ratio is a 'raw' ratio (ratio of observed events).

|  |  |
| --- | --- |
| Transitions | 1,307 |
| Transversions | 1,015 |
| Ts/Tv ratio | 1.2877 |

**All variants:**

```
Sample ,Total
Transitions ,114,102,104,112,100,166,101,96,133,138,141,1307
Transversions ,108,84,65,89,74,135,86,97,99,91,87,1015
Ts/Tv ,1,056,1,214,1,600,1,258,1,351,1,230,1,174,0,990,1,343,1,516,1,621,1,288
```

**Only known variants** (i.e. the ones having a non-empty ID field):

```
No results available (empty input?)
```

---


  **Allele frequency** 

  

|  |  |
| --- | --- |
| Min | 0 |
| Max | 0 |
| Mean | 0 |
| Median | 0 |
| Standard deviation | 0 |
| Values | 0 |
| Count | 2095 |

---


  **Allele Count** 

  

|  |  |
| --- | --- |
| Min | 0 |
| Max | 0 |
| Mean | 0 |
| Median | 0 |
| Standard deviation | 0 |
| Values | 0 |
| Count | 2095 |

---


  **Hom/Het per sample** 

  
  
  

```
Sample_names 
Reference 
Het 
Hom 
Missing
```

---


 **Codon changes**

How to read this table:   
- Rows are reference codons and columns are changed codons. E.g. Row 'AAA' column 'TAA' indicates how many 'AAA' codons have been replaced by 'TAA' codons.  
- Red background colors indicate that more changes happened (heat-map).  
- Diagonals are indicated using grey background color   
- WARNING: This table may include different translation codon tables (e.g. mamalian DNA and mitochondrial DNA).

|  | AAA | AAC | AAG | AAT | ACA | ACC | ACG | ACT | AGA | AGC | AGG | AGT | ATA | ATC | ATG | ATT | CAA | CAC | CAG | CAT | CCA | CCC | CCG | CCT | CGA | CGC | CGG | CGT | CTA | CTC | CTG | CTT | GAA | GAC | GAG | GAT | GCA | GCC | GCG | GCT | GGA | GGC | GGG | GGT | GTA | GTC | GTG | GTT | TAA | TAC | TAG | TAT | TCA | TCC | TCG | TCT | TGA | TGC | TGG | TGT | TTA | TTC | TTG | TTT |
| --- | --- | --- | --- | --- | --- | --- | --- | --- | --- | --- | --- | --- | --- | --- | --- | --- | --- | --- | --- | --- | --- | --- | --- | --- | --- | --- | --- | --- | --- | --- | --- | --- | --- | --- | --- | --- | --- | --- | --- | --- | --- | --- | --- | --- | --- | --- | --- | --- | --- | --- | --- | --- | --- | --- | --- | --- | --- | --- | --- | --- | --- | --- | --- | --- |
| AAA |  | 3 | 1 |  |  |  |  |  |  |  |  |  |  |  |  |  |  |  |  |  |  |  |  |  |  |  |  |  |  |  |  |  | 1 |  |  |  |  |  |  |  |  |  |  |  |  |  |  |  |  |  |  |  |  |  |  |  |  |  |  |  |  |  |  |  |
| AAC |  |  | 1 | 1 |  | 1 |  |  |  |  |  |  |  |  |  |  |  |  |  |  |  |  |  |  |  |  |  |  |  |  |  |  |  | 2 |  |  |  |  |  |  |  |  |  |  |  |  |  |  |  |  |  |  |  |  |  |  |  |  |  |  |  |  |  |  |
| AAG | 4 | 4 |  |  |  |  | 1 |  |  |  | 1 |  |  |  |  |  |  |  |  |  |  |  |  |  |  |  |  |  |  |  |  |  |  |  |  |  |  |  |  |  |  |  |  |  |  |  |  |  |  |  | 1 |  |  |  |  |  |  |  |  |  |  |  |  |  |
| AAT |  | 1 | 1 |  |  |  |  |  |  |  |  | 1 |  |  |  |  |  |  |  |  |  |  |  |  |  |  |  |  |  |  |  |  |  |  |  |  |  |  |  |  |  |  |  |  |  |  |  |  |  |  |  |  |  |  |  |  |  |  |  |  |  |  |  |  |
| ACA |  |  |  |  |  |  | 1 |  | 1 |  |  |  |  |  |  |  |  |  |  |  | 1 |  |  |  |  |  |  |  |  |  |  |  |  |  |  |  |  |  |  |  |  |  |  |  |  |  |  |  |  |  |  |  | 3 |  |  |  |  |  |  |  |  |  |  |  |
| ACC |  |  |  |  |  |  |  | 1 |  | 1 |  |  |  | 3 |  |  |  |  |  |  |  |  |  |  |  |  |  |  |  |  |  |  |  |  |  |  |  |  |  |  |  |  |  |  |  |  |  |  |  |  |  |  |  |  |  |  |  |  |  |  |  |  |  |  |
| ACG |  |  |  |  | 11 |  |  |  |  |  |  |  |  |  | 2 |  |  |  |  |  |  |  |  |  |  |  |  |  |  |  |  |  |  |  |  |  |  |  |  |  |  |  |  |  |  |  |  |  |  |  |  |  |  |  | 1 |  |  |  |  |  |  |  |  |  |
| ACT |  |  |  |  |  |  |  |  |  |  |  |  |  |  |  | 7 |  |  |  |  |  |  |  |  |  |  |  |  |  |  |  |  |  |  |  |  |  |  |  |  |  |  |  |  |  |  |  |  |  |  |  |  |  |  |  |  |  |  |  |  |  |  |  |  |
| AGA |  |  |  |  | 3 |  |  |  |  |  |  |  | 1 |  |  |  |  |  |  |  |  |  |  |  |  |  |  |  |  |  |  |  |  |  |  |  |  |  |  |  | 1 |  |  |  |  |  |  |  |  |  |  |  |  |  |  |  |  |  |  |  |  |  |  |  |
| AGC |  | 1 |  |  |  |  |  |  |  |  | 1 |  |  |  |  |  |  |  |  |  |  |  |  |  |  |  |  |  |  |  |  |  |  |  |  |  |  |  |  |  |  |  |  |  |  |  |  |  |  |  |  |  |  |  |  |  |  |  |  |  |  |  |  |  |
| AGG |  |  |  |  |  |  |  |  | 5 |  |  |  |  |  |  |  |  |  |  |  |  |  |  |  |  |  |  |  |  |  |  |  |  |  |  |  |  |  |  |  |  |  |  |  |  |  |  |  |  |  |  |  |  |  |  |  |  |  |  |  |  |  |  |  |
| AGT |  |  |  | 1 |  |  |  |  |  |  | 1 |  |  |  |  |  |  |  |  |  |  |  |  |  |  |  |  |  |  |  |  |  |  |  |  |  |  |  |  |  |  |  |  |  |  |  |  |  |  |  |  |  |  |  |  |  |  |  |  |  |  |  |  |  |
| ATA |  |  |  |  | 1 |  |  |  |  |  |  |  |  |  |  |  |  |  |  |  |  |  |  |  |  |  |  |  |  |  |  |  |  |  |  |  |  |  |  |  |  |  |  |  |  |  |  |  |  |  |  |  |  |  |  |  |  |  |  |  |  |  |  |  |
| ATC |  |  |  |  |  |  |  |  |  |  |  |  |  |  |  |  |  |  |  |  |  |  |  |  |  |  |  |  |  |  |  |  |  |  |  |  |  |  |  |  |  |  |  |  |  |  |  |  |  |  |  |  |  |  |  |  |  |  |  |  |  |  |  |  |
| ATG |  |  |  |  |  |  | 1 |  |  |  |  |  | 1 |  |  | 1 |  |  |  |  |  |  |  |  |  |  |  |  |  |  | 1 |  |  |  |  |  |  |  |  |  |  |  |  |  |  |  |  |  |  |  |  |  |  |  |  |  |  |  |  |  |  |  |  |  |
| ATT |  |  |  |  |  |  |  |  |  |  |  |  |  |  |  |  |  |  |  |  |  |  |  |  |  |  |  |  |  |  |  |  |  |  |  |  |  |  |  |  |  |  |  |  |  |  |  | 1 |  |  |  |  |  |  |  |  |  |  |  |  |  |  |  |  |
| CAA |  |  |  |  |  |  |  |  |  |  |  |  |  |  |  |  |  |  |  |  |  |  |  |  |  |  |  |  |  |  |  |  | 1 |  |  |  |  |  |  |  |  |  |  |  |  |  |  |  | 2 |  |  |  |  |  |  |  |  |  |  |  |  |  |  |  |
| CAC |  |  |  |  |  |  |  |  |  |  |  |  |  |  |  |  |  |  |  | 1 |  |  |  |  |  |  |  |  |  |  |  |  |  |  |  |  |  |  |  |  |  |  |  |  |  |  |  |  |  |  |  |  |  |  |  |  |  |  |  |  |  |  |  |  |
| CAG |  |  | 3 |  |  |  |  |  |  |  |  |  |  |  |  |  | 1 | 1 |  |  |  |  |  |  |  |  |  |  |  |  |  |  |  |  |  |  |  |  |  |  |  |  |  |  |  |  |  |  |  |  |  |  |  |  |  |  |  |  |  |  |  |  |  |  |
| CAT |  |  |  |  |  |  |  |  |  |  |  |  |  |  |  |  |  |  |  |  |  |  |  |  |  |  |  | 5 |  |  |  |  |  |  |  |  |  |  |  |  |  |  |  |  |  |  |  |  |  |  |  | 1 |  |  |  |  |  |  |  |  |  |  |  |  |
| CCA |  |  |  |  |  |  |  |  |  |  |  |  |  |  |  |  |  |  |  |  |  | 1 |  | 3 |  |  |  |  |  |  |  |  |  |  |  |  |  |  |  |  |  |  |  |  |  |  |  |  |  |  |  |  | 3 |  |  |  |  |  |  |  |  |  |  |  |
| CCC |  |  |  |  |  | 2 |  |  |  |  |  |  |  |  |  |  |  |  |  |  |  |  |  |  |  |  |  |  |  |  |  |  |  |  |  |  |  |  |  |  |  |  |  |  |  |  |  |  |  |  |  |  |  |  |  |  |  |  |  |  |  |  |  |  |
| CCG |  |  |  |  |  |  |  |  |  |  |  |  |  |  |  |  |  |  |  |  | 4 |  |  |  |  |  |  |  |  |  | 3 |  |  |  |  |  |  |  | 1 |  |  |  |  |  |  |  |  |  |  |  |  |  |  |  | 1 |  |  |  |  |  |  |  |  |  |
| CCT |  |  |  |  |  |  |  | 2 |  |  |  |  |  |  |  |  |  |  |  |  | 1 | 1 |  |  |  |  |  | 3 |  |  |  | 6 |  |  |  |  |  |  |  |  |  |  |  |  |  |  |  |  |  |  |  |  |  |  |  |  |  |  |  |  |  |  |  |  |
| CGA |  |  |  |  |  |  |  |  |  |  |  |  |  |  |  |  |  |  |  |  |  |  |  |  |  |  |  |  |  |  |  |  |  |  |  |  |  |  |  |  |  |  |  |  |  |  |  |  |  |  |  |  |  |  |  |  |  |  |  |  |  |  |  |  |
| CGC |  |  |  |  |  |  |  |  |  | 1 |  |  |  |  |  |  |  | 8 |  |  |  |  |  |  |  |  |  |  |  |  |  |  |  |  |  |  |  |  |  |  |  |  |  |  |  |  |  |  |  |  |  |  |  |  |  |  |  |  |  |  |  |  |  |  |
| CGG |  |  |  |  |  |  |  |  |  |  |  |  |  |  |  |  |  |  |  |  |  |  |  |  | 1 |  |  |  |  |  |  |  |  |  |  |  |  |  |  |  |  |  |  |  |  |  |  |  |  |  |  |  |  |  |  |  |  |  | 1 |  |  |  |  |  |
| CGT |  |  |  |  |  |  |  |  |  |  |  |  |  |  |  |  |  |  |  | 1 |  |  |  |  |  |  |  |  |  |  |  |  |  |  |  |  |  |  |  |  |  |  |  |  |  |  |  |  |  |  |  |  |  |  |  |  |  |  |  | 1 |  |  |  |  |
| CTA |  |  |  |  |  |  |  |  |  |  |  |  |  |  |  |  |  |  |  |  |  |  |  |  |  |  |  |  |  |  |  |  |  |  |  |  |  |  |  |  |  |  |  |  |  |  |  |  |  |  |  |  |  |  |  |  |  |  |  |  | 10 |  |  |  |
| CTC |  |  |  |  |  |  |  |  |  |  |  |  |  |  |  |  |  |  |  |  |  |  |  |  |  | 1 |  |  |  |  |  |  |  |  |  |  |  |  |  |  |  |  |  |  |  |  |  |  |  |  |  |  |  |  |  |  |  |  |  |  |  | 1 |  |  |
| CTG |  |  |  |  |  |  |  |  |  |  |  |  |  |  | 2 |  |  |  | 1 |  |  |  |  |  |  |  |  |  |  |  |  |  |  |  |  |  |  |  |  |  |  |  |  |  |  |  | 1 |  |  |  |  |  |  |  |  |  |  |  |  |  |  |  | 4 |  |
| CTT |  |  |  |  |  |  |  |  |  |  |  |  |  |  |  |  |  |  |  |  |  |  |  |  |  |  |  |  | 1 |  |  |  |  |  |  |  |  |  |  |  |  |  |  |  |  |  |  |  |  |  |  |  |  |  |  |  |  |  |  |  |  |  |  | 12 |
| GAA | 2 |  |  |  |  |  |  |  |  |  |  |  |  |  |  |  | 1 |  |  |  |  |  |  |  |  |  |  |  |  |  |  |  |  | 2 | 1 |  |  |  |  |  | 1 |  |  |  |  |  |  |  |  |  |  |  |  |  |  |  |  |  |  |  |  |  |  |  |
| GAC |  |  |  |  |  |  |  |  |  |  |  |  |  |  |  |  |  |  |  |  |  |  |  |  |  |  |  |  |  |  |  |  |  |  |  | 1 |  |  |  |  |  |  |  |  |  |  |  |  |  |  |  |  |  |  |  |  |  |  |  |  |  |  |  |  |
| GAG |  |  | 6 |  |  |  |  |  |  |  |  |  |  |  |  |  |  |  |  |  |  |  |  |  |  |  |  |  |  |  |  |  | 3 | 1 |  | 4 |  |  |  |  |  |  |  |  |  |  |  |  |  |  |  |  |  |  |  |  |  |  |  |  |  |  |  |  |
| GAT |  |  |  |  |  |  |  |  |  |  |  |  |  |  |  |  |  |  |  |  |  |  |  |  |  |  |  |  |  |  |  |  |  |  |  |  |  |  |  |  |  |  |  |  |  |  |  |  |  |  |  |  |  |  |  |  |  |  |  |  |  |  |  |  |
| GCA |  |  |  |  |  |  |  |  |  |  |  |  |  |  |  |  |  |  |  |  |  |  |  |  |  |  |  |  |  |  |  |  |  |  |  |  |  |  |  |  |  |  |  |  | 2 |  |  |  |  |  |  |  | 1 |  |  |  |  |  |  |  |  |  |  |  |
| GCC |  |  |  |  |  |  |  |  |  |  |  |  |  |  |  |  |  |  |  |  |  |  |  |  |  |  |  |  |  |  |  |  |  |  |  |  |  |  |  |  |  |  |  |  |  | 2 |  |  |  |  |  |  |  |  |  |  |  |  |  |  |  |  |  |  |
| GCG |  |  |  |  |  |  |  |  |  |  |  |  |  |  |  |  |  |  |  |  |  |  |  |  |  |  |  |  |  |  |  |  |  |  |  |  | 1 |  |  |  |  |  |  |  |  |  |  |  |  |  |  |  |  |  |  |  |  |  |  |  |  |  |  |  |
| GCT |  |  |  |  |  |  |  |  |  |  |  |  |  |  |  |  |  |  |  |  |  |  |  | 2 |  |  |  |  |  |  |  |  |  |  |  |  |  |  |  |  |  |  |  |  |  |  |  | 2 |  |  |  |  |  |  |  |  |  |  |  |  |  |  |  |  |
| GGA |  |  |  |  |  |  |  |  | 6 |  |  |  |  |  |  |  |  |  |  |  |  |  |  |  | 2 |  |  |  |  |  |  |  | 1 |  |  |  |  |  |  |  |  | 1 | 2 |  |  |  |  |  |  |  |  |  |  |  |  |  | 2 |  |  |  |  |  |  |  |
| GGC |  |  |  |  |  |  |  |  |  |  |  |  |  |  |  |  |  |  |  |  |  |  |  |  |  |  |  |  |  |  |  |  |  |  |  |  |  |  |  |  | 2 |  |  | 1 |  |  |  |  |  |  |  |  |  |  |  |  |  |  |  |  |  |  |  |  |
| GGG |  |  |  |  |  |  |  |  |  |  |  |  |  |  |  |  |  |  |  |  |  |  |  |  |  |  |  |  |  |  |  |  |  |  | 1 |  |  |  |  |  |  |  |  |  |  |  |  |  |  |  |  |  |  |  |  |  |  |  |  |  |  |  |  |  |
| GGT |  |  |  |  |  |  |  |  |  |  |  | 2 |  |  |  |  |  |  |  |  |  |  |  |  |  |  |  |  |  |  |  |  |  |  |  |  |  |  |  | 3 |  |  |  |  |  |  |  | 1 |  |  |  |  |  |  |  |  |  |  |  |  |  |  |  |  |
| GTA |  |  |  |  |  |  |  |  |  |  |  |  | 1 |  |  |  |  |  |  |  |  |  |  |  |  |  |  |  |  |  |  |  |  |  |  |  |  |  |  |  |  |  |  |  |  |  |  | 1 |  |  |  |  |  |  |  |  |  |  |  |  | 1 |  |  |  |
| GTC |  |  |  |  |  |  |  |  |  |  |  |  |  |  |  |  |  |  |  |  |  |  |  |  |  |  |  |  |  |  |  |  |  |  |  |  |  |  |  |  |  |  |  |  |  |  |  |  |  |  |  |  |  |  |  |  |  |  |  |  |  |  |  |  |
| GTG |  |  |  |  |  |  |  |  |  |  |  |  |  |  | 3 |  |  |  |  |  |  |  |  |  |  |  |  |  |  |  | 1 |  |  |  |  |  |  |  | 1 |  |  |  |  |  | 3 |  |  |  |  |  |  |  |  |  |  |  |  |  |  |  |  |  |  |  |
| GTT |  |  |  |  |  |  |  |  |  |  |  |  |  |  |  | 1 |  |  |  |  |  |  |  |  |  |  |  |  |  |  |  |  |  |  |  |  |  |  |  | 1 |  |  |  |  |  |  |  |  |  |  |  |  |  |  |  |  |  |  |  |  |  |  |  | 1 |
| TAA |  |  |  |  |  |  |  |  |  |  |  |  |  |  |  |  |  |  |  |  |  |  |  |  |  |  |  |  |  |  |  |  |  |  |  |  |  |  |  |  |  |  |  |  |  |  |  |  |  |  |  |  |  |  |  |  |  |  |  |  |  |  |  |  |
| TAC |  |  |  |  |  |  |  |  |  |  |  |  |  |  |  |  |  |  |  |  |  |  |  |  |  |  |  |  |  |  |  |  |  |  |  |  |  |  |  |  |  |  |  |  |  |  |  |  | 1 |  |  | 3 |  | 1 |  |  |  |  |  |  |  |  |  |  |
| TAG |  |  |  |  |  |  |  |  |  |  |  |  |  |  |  |  |  |  |  |  |  |  |  |  |  |  |  |  |  |  |  |  |  |  |  |  |  |  |  |  |  |  |  |  |  |  |  |  |  |  |  |  |  |  |  |  |  |  |  |  |  |  |  |  |
| TAT |  |  |  |  |  |  |  |  |  |  |  |  |  |  |  |  |  |  |  |  |  |  |  |  |  |  |  |  |  |  |  |  |  |  |  |  |  |  |  |  |  |  |  |  |  |  |  |  |  |  |  |  |  |  |  |  |  |  |  |  |  |  |  |  |
| TCA |  |  |  |  |  |  |  |  |  |  |  |  |  |  |  |  |  |  |  |  | 2 |  |  |  |  |  |  |  |  |  |  |  |  |  |  |  |  |  |  |  |  |  |  |  |  |  |  |  |  |  |  |  |  |  | 1 |  | 4 |  |  |  | 1 |  |  |  |
| TCC |  |  |  |  |  |  |  |  |  |  |  |  |  |  |  |  |  |  |  |  |  |  |  |  |  |  |  |  |  |  |  |  |  |  |  |  |  |  |  |  |  |  |  |  |  |  |  |  |  |  |  |  |  |  |  | 10 |  |  |  |  |  | 1 |  |  |
| TCG |  |  |  |  |  |  |  |  |  |  |  |  |  |  |  |  |  |  |  |  |  |  |  |  |  |  |  |  |  |  |  |  |  |  |  |  |  |  |  |  |  |  |  |  |  |  |  |  |  |  |  |  | 3 |  |  |  |  |  | 1 |  |  |  |  |  |
| TCT |  |  |  |  |  |  |  |  |  |  |  |  |  |  |  |  |  |  |  |  |  |  |  |  |  |  |  |  |  |  |  |  |  |  |  |  |  |  |  |  |  |  |  |  |  |  |  |  |  |  |  |  |  |  |  |  |  |  |  |  |  |  |  | 2 |
| TGA |  |  |  |  |  |  |  |  |  |  |  |  |  |  |  |  |  |  |  |  |  |  |  |  |  |  |  |  |  |  |  |  |  |  |  |  |  |  |  |  |  |  |  |  |  |  |  |  |  |  |  |  |  |  |  |  |  |  |  |  |  |  |  |  |
| TGC |  |  |  |  |  |  |  |  |  |  |  |  |  |  |  |  |  |  |  |  |  |  |  |  |  |  |  |  |  |  |  |  |  |  |  |  |  |  |  |  |  |  |  |  |  |  |  |  |  | 7 |  |  |  |  |  |  | 1 |  |  |  |  |  |  |  |
| TGG |  |  |  |  |  |  |  |  |  |  |  |  |  |  |  |  |  |  |  |  |  |  |  |  |  |  |  |  |  |  |  |  |  |  |  |  |  |  |  |  |  |  |  |  |  |  |  |  |  |  | 5 |  |  |  | 3 |  | 1 |  |  |  |  |  |  |  |
| TGT |  |  |  |  |  |  |  |  |  |  |  |  |  |  |  |  |  |  |  |  |  |  |  |  |  |  |  |  |  |  |  |  |  |  |  |  |  |  |  |  |  |  |  |  |  |  |  |  |  |  |  | 2 |  |  |  | 1 |  |  |  |  |  |  |  |  |
| TTA |  |  |  |  |  |  |  |  |  |  |  |  |  |  |  |  |  |  |  |  |  |  |  |  |  |  |  |  |  |  |  |  |  |  |  |  |  |  |  |  |  |  |  |  |  |  |  |  |  |  |  |  |  |  |  |  |  |  |  |  |  |  |  |  |
| TTC |  |  |  |  |  |  |  |  |  |  |  |  |  |  |  |  |  |  |  |  |  |  |  |  |  |  |  |  |  |  |  |  |  |  |  |  |  |  |  |  |  |  |  |  |  |  |  |  |  |  |  |  |  |  |  |  |  |  |  |  |  |  |  | 1 |
| TTG |  |  |  |  |  |  |  |  |  |  |  |  |  |  |  |  |  |  |  |  |  |  |  |  |  |  |  |  |  |  | 3 |  |  |  |  |  |  |  |  |  |  |  |  |  |  |  |  |  |  |  |  |  |  |  |  |  |  |  |  |  | 2 |  |  | 1 |
| TTT |  |  |  |  |  |  |  |  |  |  |  |  |  |  |  |  |  |  |  |  |  |  |  |  |  |  |  |  |  |  |  |  |  |  |  |  |  |  |  |  |  |  |  |  |  |  |  |  |  |  |  |  |  |  |  | 2 |  |  |  |  |  |  |  |  |


---


 **Amino acid changes**

How to read this table:   
- Rows are reference amino acids and columns are changed amino acids. E.g. Row 'A' column 'E' indicates how many 'A' amino acids have been replaced by 'E' amino acids.  
- Red background colors indicate that more changes happened (heat-map).  
- Diagonals are indicated using grey background color   
- WARNING: This table may include different translation codon tables (e.g. mamalian DNA and mitochondrial DNA).

|  | \* | A | C | D | E | F | G | H | I | K | L | M | N | P | Q | R | S | T | V | W | Y |
| --- | --- | --- | --- | --- | --- | --- | --- | --- | --- | --- | --- | --- | --- | --- | --- | --- | --- | --- | --- | --- | --- |
| \* |  |  |  |  |  |  |  |  |  |  |  |  |  |  |  |  |  |  |  |  |  |
| A |  | 1 |  |  |  |  |  |  |  |  |  |  |  | 2 |  |  | 1 |  | 6 |  |  |
| C | 1 |  |  |  |  |  |  |  |  |  |  |  |  |  |  |  | 1 |  |  |  | 9 |
| D |  |  |  | 1 |  |  |  |  |  |  |  |  |  |  |  |  |  |  |  |  |  |
| E |  |  |  | 7 | 4 |  | 1 |  |  | 8 |  |  |  |  | 1 |  |  |  |  |  |  |
| F |  |  |  |  |  | 1 |  |  |  |  |  |  |  |  |  |  | 2 |  |  |  |  |
| G | 2 | 3 |  |  | 2 |  | 6 |  |  |  |  |  |  |  |  | 8 | 2 |  | 1 |  |  |
| H |  |  |  |  |  |  |  | 1 |  |  |  |  |  |  |  | 5 |  |  |  |  | 1 |
| I |  |  |  |  |  |  |  |  |  |  |  |  |  |  |  |  |  | 1 | 1 |  |  |
| K | 1 |  |  |  | 1 |  |  |  |  | 5 |  |  | 7 |  |  | 1 |  | 1 |  |  |  |
| L |  |  |  |  |  | 14 |  |  |  |  | 20 | 2 |  |  | 1 | 1 |  |  | 1 |  |  |
| M |  |  |  |  |  |  |  |  | 2 |  | 1 |  |  |  |  |  |  | 1 |  |  |  |
| N |  |  |  | 2 |  |  |  |  |  | 2 |  |  | 2 |  |  |  | 1 | 1 |  |  |  |
| P |  | 1 |  |  |  |  |  |  |  |  | 9 |  |  | 10 |  | 3 | 4 | 4 |  |  |  |
| Q | 2 |  |  |  | 1 |  |  | 1 |  | 3 |  |  |  |  | 1 |  |  |  |  |  |  |
| R |  |  | 1 |  |  |  | 1 | 9 | 1 |  |  |  |  |  |  | 6 | 1 | 3 |  | 1 |  |
| S | 4 |  |  |  |  | 3 |  |  |  |  | 1 |  | 2 | 2 |  | 2 | 14 |  |  | 1 |  |
| T |  |  |  |  |  |  |  |  | 10 |  |  | 2 |  | 1 |  | 1 | 5 | 13 |  |  |  |
| V |  | 2 |  |  |  | 1 |  |  | 2 |  | 2 | 3 |  |  |  |  |  |  | 4 |  |  |
| W | 6 |  |  |  |  |  |  |  |  |  |  |  |  |  |  |  | 3 |  |  |  |  |
| Y | 1 |  |  |  |  |  |  |  |  |  |  |  |  |  |  |  | 1 |  |  |  | 3 |


---


 **Variants by chromosome**

```
		  

		1, Position,0,1000000,2000000,3000000,4000000,5000000,6000000,7000000,8000000,9000000,10000000,11000000,12000000,13000000,14000000,15000000,16000000,17000000,18000000,19000000,20000000,21000000,22000000,23000000,24000000,25000000,26000000,27000000,28000000,29000000,30000000
1,Count,5,2,5,11,3,4,1,2,4,6,6,5,9,13,77,175,54,10,2,5,5,0,6,2,5,2,0,5,1,9,2

	
```

```
		  

		2, Position,0,100000,200000,300000,400000,500000,600000,700000,800000,900000,1000000,1100000,1200000,1300000,1400000,1500000,1600000,1700000,1800000,1900000,2000000,2100000,2200000,2300000,2400000,2500000,2600000,2700000,2800000,2900000,3000000,3100000,3200000,3300000,3400000,3500000,3600000,3700000,3800000,3900000,4000000,4100000,4200000,4300000,4400000,4500000,4600000,4700000,4800000,4900000,5000000,5100000,5200000,5300000,5400000,5500000,5600000,5700000,5800000,5900000,6000000,6100000,6200000,6300000,6400000,6500000,6600000,6700000,6800000,6900000,7000000,7100000,7200000,7300000,7400000,7500000,7600000,7700000,7800000,7900000,8000000,8100000,8200000,8300000,8400000,8500000,8600000,8700000,8800000,8900000,9000000,9100000,9200000,9300000,9400000,9500000,9600000,9700000,9800000,9900000,10000000,10100000,10200000,10300000,10400000,10500000,10600000,10700000,10800000,10900000,11000000,11100000,11200000,11300000,11400000,11500000,11600000,11700000,11800000,11900000,12000000,12100000,12200000,12300000,12400000,12500000,12600000,12700000,12800000,12900000,13000000,13100000,13200000,13300000,13400000,13500000,13600000,13700000,13800000,13900000,14000000,14100000,14200000,14300000,14400000,14500000,14600000,14700000,14800000,14900000,15000000,15100000,15200000,15300000,15400000,15500000,15600000,15700000,15800000,15900000,16000000,16100000,16200000,16300000,16400000,16500000,16600000,16700000,16800000,16900000,17000000,17100000,17200000,17300000,17400000,17500000,17600000,17700000,17800000,17900000,18000000,18100000,18200000,18300000,18400000,18500000,18600000,18700000,18800000,18900000,19000000,19100000,19200000,19300000,19400000,19500000,19600000
2,Count,5,0,1,0,0,0,0,0,1,0,0,0,1,1,0,0,0,1,0,0,1,0,0,0,3,6,2,6,1,1,5,7,3,2,3,4,66,15,5,7,3,8,2,4,1,4,11,3,3,7,6,0,3,0,6,4,3,0,0,0,0,8,0,0,2,0,0,0,0,1,8,0,0,0,0,1,0,2,0,0,1,1,0,1,0,0,0,1,0,1,0,0,1,0,0,0,1,1,0,0,0,0,0,0,0,0,1,0,0,0,0,1,1,0,0,0,0,1,0,1,2,0,0,0,0,0,0,2,0,0,0,0,0,0,0,0,1,0,1,0,3,0,0,0,1,3,0,0,0,0,0,0,1,0,0,1,0,0,0,0,1,1,1,0,1,0,1,0,0,0,0,1,0,0,0,0,1,0,0,5,0,0,0,0,0,0,0,0,0,0,0,0,0,0,0,0,1

	
```

```
		  

		3, Position,0,100000,200000,300000,400000,500000,600000,700000,800000,900000,1000000,1100000,1200000,1300000,1400000,1500000,1600000,1700000,1800000,1900000,2000000,2100000,2200000,2300000,2400000,2500000,2600000,2700000,2800000,2900000,3000000,3100000,3200000,3300000,3400000,3500000,3600000,3700000,3800000,3900000,4000000,4100000,4200000,4300000,4400000,4500000,4600000,4700000,4800000,4900000,5000000,5100000,5200000,5300000,5400000,5500000,5600000,5700000,5800000,5900000,6000000,6100000,6200000,6300000,6400000,6500000,6600000,6700000,6800000,6900000,7000000,7100000,7200000,7300000,7400000,7500000,7600000,7700000,7800000,7900000,8000000,8100000,8200000,8300000,8400000,8500000,8600000,8700000,8800000,8900000,9000000,9100000,9200000,9300000,9400000,9500000,9600000,9700000,9800000,9900000,10000000,10100000,10200000,10300000,10400000,10500000,10600000,10700000,10800000,10900000,11000000,11100000,11200000,11300000,11400000,11500000,11600000,11700000,11800000,11900000,12000000,12100000,12200000,12300000,12400000,12500000,12600000,12700000,12800000,12900000,13000000,13100000,13200000,13300000,13400000,13500000,13600000,13700000,13800000,13900000,14000000,14100000,14200000,14300000,14400000,14500000,14600000,14700000,14800000,14900000,15000000,15100000,15200000,15300000,15400000,15500000,15600000,15700000,15800000,15900000,16000000,16100000,16200000,16300000,16400000,16500000,16600000,16700000,16800000,16900000,17000000,17100000,17200000,17300000,17400000,17500000,17600000,17700000,17800000,17900000,18000000,18100000,18200000,18300000,18400000,18500000,18600000,18700000,18800000,18900000,19000000,19100000,19200000,19300000,19400000,19500000,19600000,19700000,19800000,19900000,20000000,20100000,20200000,20300000,20400000,20500000,20600000,20700000,20800000,20900000,21000000,21100000,21200000,21300000,21400000,21500000,21600000,21700000,21800000,21900000,22000000,22100000,22200000,22300000,22400000,22500000,22600000,22700000,22800000,22900000,23000000,23100000,23200000,23300000,23400000
3,Count,0,0,1,2,0,2,0,0,0,0,0,0,1,0,0,1,0,0,0,0,0,0,0,0,1,0,0,0,0,0,0,0,0,0,0,0,1,1,0,0,0,2,1,0,0,0,0,0,1,2,0,0,0,0,0,0,0,1,0,0,0,1,0,0,0,0,0,0,1,1,0,1,0,0,0,0,0,0,1,0,0,2,0,0,1,0,0,0,0,0,0,2,0,0,0,1,0,0,1,0,0,0,0,0,1,1,0,1,1,0,0,2,0,0,2,2,1,1,0,0,4,3,24,2,3,4,0,1,0,2,0,4,9,0,1,70,17,67,69,4,2,34,87,44,13,11,5,1,5,1,0,17,10,6,22,0,1,9,1,0,2,0,1,1,0,1,1,0,0,3,0,0,5,2,1,0,0,0,0,0,0,0,0,1,0,0,4,1,1,0,0,0,0,0,0,0,0,1,2,0,0,0,0,0,2,0,0,0,1,1,0,0,0,0,0,0,0,0,0,0,2,0,0,0,0,0,0,0,0,1,0,0,0,0,0

	
```

```
		  

		4, Position,0,100000,200000,300000,400000,500000,600000,700000,800000,900000,1000000,1100000,1200000,1300000,1400000,1500000,1600000,1700000,1800000,1900000,2000000,2100000,2200000,2300000,2400000,2500000,2600000,2700000,2800000,2900000,3000000,3100000,3200000,3300000,3400000,3500000,3600000,3700000,3800000,3900000,4000000,4100000,4200000,4300000,4400000,4500000,4600000,4700000,4800000,4900000,5000000,5100000,5200000,5300000,5400000,5500000,5600000,5700000,5800000,5900000,6000000,6100000,6200000,6300000,6400000,6500000,6600000,6700000,6800000,6900000,7000000,7100000,7200000,7300000,7400000,7500000,7600000,7700000,7800000,7900000,8000000,8100000,8200000,8300000,8400000,8500000,8600000,8700000,8800000,8900000,9000000,9100000,9200000,9300000,9400000,9500000,9600000,9700000,9800000,9900000,10000000,10100000,10200000,10300000,10400000,10500000,10600000,10700000,10800000,10900000,11000000,11100000,11200000,11300000,11400000,11500000,11600000,11700000,11800000,11900000,12000000,12100000,12200000,12300000,12400000,12500000,12600000,12700000,12800000,12900000,13000000,13100000,13200000,13300000,13400000,13500000,13600000,13700000,13800000,13900000,14000000,14100000,14200000,14300000,14400000,14500000,14600000,14700000,14800000,14900000,15000000,15100000,15200000,15300000,15400000,15500000,15600000,15700000,15800000,15900000,16000000,16100000,16200000,16300000,16400000,16500000,16600000,16700000,16800000,16900000,17000000,17100000,17200000,17300000,17400000,17500000,17600000,17700000,17800000,17900000,18000000,18100000,18200000,18300000,18400000,18500000
4,Count,1,0,0,0,0,0,1,0,3,0,1,0,3,0,1,1,1,15,1,1,6,0,1,1,0,1,0,1,1,13,6,20,0,6,3,23,9,4,10,88,30,4,13,6,0,15,2,1,2,4,4,8,0,1,0,0,3,0,0,1,5,1,1,0,1,0,0,0,0,1,0,0,1,0,1,6,0,0,1,0,0,0,0,1,0,0,0,0,0,3,0,4,1,0,0,0,0,0,1,1,0,0,1,2,0,0,3,0,0,0,1,0,2,0,1,0,1,1,1,0,0,0,0,1,0,1,0,0,1,0,2,0,0,0,0,0,0,1,0,0,0,0,0,0,0,0,1,0,1,0,0,0,0,1,0,0,0,0,0,0,0,0,0,0,1,0,0,0,0,0,1,0,0,1,0,0,0,0,0,0,0,0,0,0,1,0

	
```

```
		  

		5, Position,0,100000,200000,300000,400000,500000,600000,700000,800000,900000,1000000,1100000,1200000,1300000,1400000,1500000,1600000,1700000,1800000,1900000,2000000,2100000,2200000,2300000,2400000,2500000,2600000,2700000,2800000,2900000,3000000,3100000,3200000,3300000,3400000,3500000,3600000,3700000,3800000,3900000,4000000,4100000,4200000,4300000,4400000,4500000,4600000,4700000,4800000,4900000,5000000,5100000,5200000,5300000,5400000,5500000,5600000,5700000,5800000,5900000,6000000,6100000,6200000,6300000,6400000,6500000,6600000,6700000,6800000,6900000,7000000,7100000,7200000,7300000,7400000,7500000,7600000,7700000,7800000,7900000,8000000,8100000,8200000,8300000,8400000,8500000,8600000,8700000,8800000,8900000,9000000,9100000,9200000,9300000,9400000,9500000,9600000,9700000,9800000,9900000,10000000,10100000,10200000,10300000,10400000,10500000,10600000,10700000,10800000,10900000,11000000,11100000,11200000,11300000,11400000,11500000,11600000,11700000,11800000,11900000,12000000,12100000,12200000,12300000,12400000,12500000,12600000,12700000,12800000,12900000,13000000,13100000,13200000,13300000,13400000,13500000,13600000,13700000,13800000,13900000,14000000,14100000,14200000,14300000,14400000,14500000,14600000,14700000,14800000,14900000,15000000,15100000,15200000,15300000,15400000,15500000,15600000,15700000,15800000,15900000,16000000,16100000,16200000,16300000,16400000,16500000,16600000,16700000,16800000,16900000,17000000,17100000,17200000,17300000,17400000,17500000,17600000,17700000,17800000,17900000,18000000,18100000,18200000,18300000,18400000,18500000,18600000,18700000,18800000,18900000,19000000,19100000,19200000,19300000,19400000,19500000,19600000,19700000,19800000,19900000,20000000,20100000,20200000,20300000,20400000,20500000,20600000,20700000,20800000,20900000,21000000,21100000,21200000,21300000,21400000,21500000,21600000,21700000,21800000,21900000,22000000,22100000,22200000,22300000,22400000,22500000,22600000,22700000,22800000,22900000,23000000,23100000,23200000,23300000,23400000,23500000,23600000,23700000,23800000,23900000,24000000,24100000,24200000,24300000,24400000,24500000,24600000,24700000,24800000,24900000,25000000,25100000,25200000,25300000,25400000,25500000,25600000,25700000,25800000,25900000,26000000,26100000,26200000,26300000,26400000,26500000,26600000,26700000,26800000,26900000
5,Count,1,1,1,0,2,0,0,0,0,0,1,0,0,1,1,0,0,1,1,0,0,0,1,0,0,0,1,0,1,0,0,1,1,0,1,0,0,0,0,1,0,1,1,1,0,0,0,0,0,1,1,1,0,0,0,0,1,1,0,5,0,0,0,0,0,1,1,0,0,1,0,0,0,0,1,1,0,1,0,0,1,0,3,1,0,1,1,0,2,1,0,0,1,0,0,0,0,1,1,0,7,1,0,0,1,0,1,7,1,3,8,3,7,11,1,0,1,90,6,7,30,11,5,8,6,1,0,2,12,12,0,1,11,13,4,9,2,2,1,0,0,2,0,1,0,1,2,1,0,0,0,0,0,0,0,1,2,0,0,3,0,0,0,0,0,0,0,0,0,0,1,0,0,0,0,0,1,0,0,0,0,0,0,0,0,0,0,0,0,0,0,0,1,0,0,0,0,0,0,0,0,1,0,0,1,0,0,0,0,1,0,1,1,9,0,1,0,1,0,0,0,0,0,0,0,0,0,1,0,1,1,0,0,0,0,1,0,0,1,0,4,0,0,0,0,0,0,0,0,0,0,1,0,0,1,0,0,0,1,0,1,0,1,0,0,0,1,0,0,1

	
```


---

 **Details by gene** 

**Here** you can find a tab-separated table.
