# Supplementary material for: Genomic impact of stress-induced transposable element mobility in Arabidopsis
Source: Nucleic Acids Res. 2021 Sep 22;49(18):10431–47. doi: 10.1093/nar/gkab828 (PMC8501995; doi:10.1093/nar/gkab828)
Supplement: gkab828_Supplemental_Files [file gkab828_supplemental_files.zip › Supplementary File 5 - hclines SNP INDELS CNV.zip!/snpEff_hclines_private_indels.html]

### SnpEff: Variant analysis

**Contents**
Summary  
 Variant rate by chromosome  
Variants by type  
 Number of variants by impact   
 Number of variants by functional class   
 Number of variants by effect   
Quality histogram  
InDel length histogram  
Base variant table  
Transition vs transversions (ts/tv)  
 Allele frequency   
 Allele Count   
 Codon change table   
 Amino acid change table   
 Chromosome variants plots   
 Details by gene


---


**Summary**

|  |  |
| --- | --- |
| **Genome** | Arabidopsis\_thaliana |
| **Date** | 2021-06-28 15:15 |
| **SnpEff version** | ``` SnpEff 5.0e (build 2021-03-09 06:01), by Pablo Cingolani ``` |
| **Command line arguments** | ``` SnpEff  Arabidopsis_thaliana hclines_private_indels.vcf ``` |
| **Warnings** | 316 |
| **Errors** | 0 |
| **Number of lines (input file)** | 1,603 |
| **Number of variants (before filter)** | 1,603 |
| **Number of not variants  (i.e. reference equals alternative)** | 0 |
| **Number of variants processed   (i.e. after filter and non-variants)** | 1,553 |
| **Number of known variants  (i.e. non-empty ID)** | 0 ( 0% ) |
| **Number of multi-allelic VCF entries  (i.e. more than two alleles)** | 0 |
| **Number of effects** | 8,865 |
| **Genome total length** | 119,667,800 |
| **Genome effective length** | 119,146,348 |
| **Variant rate** | 1 variant every 76,720 bases |


---


 **Variants rate details** 

| Chromosome | Length | Variants | Variants rate |
| --- | --- | --- | --- |
| 1 | 30,427,671 | 377 | 80,710 |
| 2 | 19,698,289 | 256 | 76,946 |
| 3 | 23,459,830 | 363 | 64,627 |
| 4 | 18,585,056 | 219 | 84,863 |
| 5 | 26,975,502 | 338 | 79,809 |
| Total | 119,146,348 | 1,553 | 76,720 |


---


 **Number variants by type**

| **Type** | **Total** |
| --- | --- |
| **SNP** | 0 |
| **MNP** | 0 |
| **INS** | 893 |
| **DEL** | 660 |
| **MIXED** | 0 |
| **INV** | 0 |
| **DUP** | 0 |
| **BND** | 0 |
| **INTERVAL** | 0 |
| **Total** | 1,553 |
| --- | --- |


---


 **Number of effects by impact** 

| **Type (alphabetical order)** |  | Count | Percent |
| --- | --- | --- | --- |
| **HIGH** |  | 77 | 0.869% |
| **LOW** |  | 36 | 0.406% |
| **MODERATE** |  | 10 | 0.113% |
| **MODIFIER** |  | 8,742 | 98.613% |


---


 **Number of effects by functional class** 

| **Type (alphabetical order)** |  | Count | Percent |
| --- | --- | --- | --- |

  

Missense / Silent ratio: 0


---


 **Number of effects by type and region** 

| Type | Region |
| --- | --- |
| **Type (alphabetical order)** \| \| Count \| Percent; **3\_prime\_UTR\_variant** \| \| 181 \| 2.022%; **5\_prime\_UTR\_variant** \| \| 189 \| 2.111%; **conservative\_inframe\_deletion** \| \| 4 \| 0.045%; **conservative\_inframe\_insertion** \| \| 1 \| 0.011%; **disruptive\_inframe\_deletion** \| \| 4 \| 0.045%; **disruptive\_inframe\_insertion** \| \| 5 \| 0.056%; **downstream\_gene\_variant** \| \| 3,220 \| 35.97%; **frameshift\_variant** \| \| 68 \| 0.76%; **intergenic\_region** \| \| 1,012 \| 11.305%; **intron\_variant** \| \| 585 \| 6.535%; **non\_coding\_transcript\_exon\_variant** \| \| 18 \| 0.201%; **splice\_acceptor\_variant** \| \| 9 \| 0.101%; **splice\_donor\_variant** \| \| 7 \| 0.078%; **splice\_region\_variant** \| \| 47 \| 0.525%; **start\_lost** \| \| 6 \| 0.067%; **stop\_gained** \| \| 28 \| 0.313%; **upstream\_gene\_variant** \| \| 3,568 \| 39.857% | **Type (alphabetical order)** \| \| Count \| Percent; **DOWNSTREAM** \| \| 3,220 \| 36.323%; **EXON** \| \| 100 \| 1.128%; **INTERGENIC** \| \| 1,012 \| 11.416%; **INTRON** \| \| 554 \| 6.249%; **SPLICE\_SITE\_ACCEPTOR** \| \| 5 \| 0.056%; **SPLICE\_SITE\_REGION** \| \| 36 \| 0.406%; **UPSTREAM** \| \| 3,568 \| 40.248%; **UTR\_3\_PRIME** \| \| 181 \| 2.042%; **UTR\_5\_PRIME** \| \| 189 \| 2.132% |


---


 **Quality:**

```
|  |  |
| --- | --- |
| Min | 34 |
| Max | 1,291 |
| Mean | 105.806 |
| Median | 73 |
| Standard deviation | 105.885 |
| Values | 34,35,36,37,38,39,40,41,42,43,44,45,46,47,48,49,50,51,52,53,54,55,56,57,58,59,60,61,62,63,64,65,66,67,68,69,70,71,72,73,74,75,76,77,78,79,80,81,82,83,84,85,86,87,88,89,90,91,92,93,94,95,96,97,98,99,100,101,102,103,104,105,106,107,108,109,110,111,112,113,114,115,116,117,118,120,121,122,123,124,125,126,127,128,130,131,132,133,134,135,136,137,138,139,140,141,142,144,145,146,148,151,152,154,155,156,157,159,160,163,164,166,167,169,170,171,172,175,178,179,180,181,185,186,187,189,190,191,192,193,195,196,199,201,202,206,208,210,211,214,217,219,220,223,224,225,226,227,229,230,231,232,233,235,236,242,244,247,250,251,253,255,256,261,262,265,268,269,272,274,275,276,283,286,289,292,295,296,298,306,307,308,309,310,311,313,316,319,320,322,326,332,343,346,348,349,352,353,366,369,388,406,419,426,437,444,445,450,464,471,473,482,484,488,497,509,526,532,547,567,597,616,645,698,706,715,755,786,803,887,1066,1291 |
| Count | 2,1,1,7,4,6,5,7,5,3,11,13,13,17,13,18,14,36,29,21,34,20,23,35,27,29,36,33,36,30,22,27,37,26,23,27,25,20,22,20,26,20,20,17,19,10,18,15,17,15,12,14,15,17,14,10,6,14,5,17,11,6,10,2,4,10,7,1,9,6,6,10,9,5,9,8,9,5,3,3,1,5,4,7,10,1,4,3,2,4,6,1,3,6,5,2,4,9,1,2,6,1,1,8,1,2,6,1,3,1,4,3,2,1,1,1,3,1,5,6,1,5,1,1,1,1,7,8,3,2,1,4,1,1,4,2,2,1,1,5,1,3,8,1,2,1,3,1,1,2,3,1,3,1,1,1,2,2,2,1,1,1,1,4,1,2,1,2,3,1,3,1,5,1,1,2,2,1,1,2,3,1,1,1,2,2,1,1,1,2,1,1,1,3,1,1,2,1,1,1,1,1,1,1,1,1,1,1,2,1,2,1,1,1,1,1,2,1,1,2,1,1,1,1,1,1,1,1,1,1,1,1,1,1,1,1,1,1,1,1,1,3 |
```


---


 **Insertions and deletions length:**

```
|  |  |
| --- | --- |
| Min | 0 |
| Max | 177 |
| Mean | 2.341 |
| Median | 1 |
| Standard deviation | 12.079 |
| Values | 0,1,2,3,4,5,6,9,10,11,12,13,14,16,17,18,20,23,26,29,30,31,34,35,36,38,39,40,41,42,45,49,50,52,53,54,55,56,57,60,64,67,71,77,79,80,86,102,157,176,177 |
| Count | 769,695,10,12,1,7,3,1,2,2,2,1,2,1,1,1,1,1,1,2,1,1,2,1,1,1,1,1,1,1,1,2,1,2,1,1,1,1,2,1,2,2,1,1,1,1,1,1,1,1,2 |
```


---


 **Base changes (SNPs)** 

|  |  |  |  |  |
| --- | --- | --- | --- | --- |
|  | **A** | **C** | **G** | **T** |
| **A** | 0 | 0 | 0 | 0 |
| **C** | 0 | 0 | 0 | 0 |
| **G** | 0 | 0 | 0 | 0 |
| **T** | 0 | 0 | 0 | 0 |

---


  **Ts/Tv (transitions / transversions)** 


**Note:** Only SNPs are used for this statistic.  
**Note:** This Ts/Tv ratio is a 'raw' ratio (ratio of observed events).

|  |  |
| --- | --- |
| Transitions | 0 |
| Transversions | 0 |
| Ts/Tv ratio | 0 |

**All variants:**

```
No results available (empty input?)
```

**Only known variants** (i.e. the ones having a non-empty ID field):

```
No results available (empty input?)
```

---


  **Allele frequency** 

  

|  |  |
| --- | --- |
| Min | 0 |
| Max | 0 |
| Mean | 0 |
| Median | 0 |
| Standard deviation | 0 |
| Values | 0 |
| Count | 1553 |

---


  **Allele Count** 

  

|  |  |
| --- | --- |
| Min | 0 |
| Max | 0 |
| Mean | 0 |
| Median | 0 |
| Standard deviation | 0 |
| Values | 0 |
| Count | 1553 |

---


  **Hom/Het per sample** 

  
  
  

```
Sample_names 
Reference 
Het 
Hom 
Missing
```

---


 **Codon changes**

How to read this table:   
- Rows are reference codons and columns are changed codons. E.g. Row 'AAA' column 'TAA' indicates how many 'AAA' codons have been replaced by 'TAA' codons.  
- Red background colors indicate that more changes happened (heat-map).  
- Diagonals are indicated using grey background color   
- WARNING: This table may include different translation codon tables (e.g. mamalian DNA and mitochondrial DNA).

|  | - | AAA | AAC | AAG | AAT | ACA | ACC | ACT | AGA | AGC | AGG | AGT | ATC | ATG | ATT | CAA | CAC | CAG | CAT | CCA | CCT | CGA | CGC | CTA | CTC | CTG | CTT | GAA | GAC | GAG | GAT | GCA | GCC | GCG | GCT | GGA | GGC | GGG | GGT | GTA | GTG | GTT | TAA | TAC | TAG | TAT | TCA | TCC | TCT | TGA | TGG | TGT | TTA | TTC | TTG | TTT |
| --- | --- | --- | --- | --- | --- | --- | --- | --- | --- | --- | --- | --- | --- | --- | --- | --- | --- | --- | --- | --- | --- | --- | --- | --- | --- | --- | --- | --- | --- | --- | --- | --- | --- | --- | --- | --- | --- | --- | --- | --- | --- | --- | --- | --- | --- | --- | --- | --- | --- | --- | --- | --- | --- | --- | --- | --- |
| - |  | 11 | 9 | 8 | 34 | 2 |  | 7 | 7 | 1 |  | 15 | 17 |  | 17 | 19 | 1 |  |  | 3 | 1 |  |  | 1 | 3 |  | 9 | 13 |  | 8 | 7 |  |  |  |  | 12 |  |  | 5 | 2 | 5 | 18 | 41 | 4 | 2 | 8 | 10 | 4 | 7 | 18 | 4 |  | 26 | 4 | 19 | 31 |
| AAA | 3 |  |  |  |  |  |  |  |  |  |  |  |  |  |  |  |  |  |  |  |  |  |  |  |  |  |  |  |  |  |  |  |  |  |  |  |  |  |  |  |  |  |  |  |  |  |  |  |  |  |  |  |  |  |  |  |
| AAC |  |  | 1 |  |  |  |  |  |  |  |  |  |  |  |  |  |  |  |  |  |  |  |  |  |  |  |  |  |  |  |  |  |  |  |  |  |  |  |  |  |  |  |  |  |  |  |  |  |  |  |  |  |  |  |  |  |
| AAG | 1 |  |  |  |  |  |  |  | 3 |  |  |  |  |  |  |  |  |  |  |  |  |  |  |  |  |  |  |  |  |  |  |  |  |  |  |  |  |  |  |  |  |  |  |  |  |  |  |  |  |  |  | 3 |  |  |  |  |
| AAT |  |  |  |  |  |  |  |  |  |  |  |  |  |  |  |  |  |  |  |  |  |  |  |  |  |  |  |  |  |  |  |  |  |  |  |  |  |  |  |  |  |  |  |  |  |  |  |  |  |  |  |  |  |  |  |  |
| ACA | 1 |  |  |  |  |  |  |  |  |  |  |  |  |  |  |  |  |  |  |  |  |  |  |  |  |  |  |  |  |  |  |  |  |  |  |  |  |  |  |  |  |  |  |  |  |  |  |  |  |  |  |  |  |  |  |  |
| ACC | 2 |  |  |  |  |  |  |  |  |  |  |  |  |  |  |  |  |  |  |  |  |  |  |  |  |  |  |  |  |  |  |  |  |  |  |  |  |  |  |  |  |  |  |  |  |  |  |  |  |  |  |  |  |  |  |  |
| ACT | 3 |  |  |  |  |  |  |  |  |  |  |  |  |  |  |  |  |  |  |  |  |  |  |  |  |  |  |  |  |  |  |  |  |  |  |  |  |  |  |  |  |  |  |  |  |  |  |  |  |  |  |  |  |  |  |  |
| AGA | 1 |  |  |  |  |  |  |  |  |  |  |  |  |  |  |  |  |  |  |  |  |  |  |  |  |  |  |  |  |  |  |  |  |  |  |  |  |  |  |  |  |  |  |  |  |  |  |  |  |  |  |  |  |  |  |  |
| AGC | 1 |  |  |  |  |  |  |  |  |  |  |  |  |  |  |  |  |  |  |  |  |  |  |  |  |  |  |  |  |  |  |  |  |  |  |  |  |  |  |  |  |  |  |  |  |  |  |  |  |  |  |  |  |  |  |  |
| AGG | 1 |  |  |  |  |  |  |  |  |  |  |  |  |  |  |  |  |  |  |  |  |  |  |  |  |  |  |  |  |  |  |  |  |  |  |  |  |  |  |  |  |  |  |  |  |  |  |  |  |  |  |  |  |  |  |  |
| AGT |  |  |  |  |  |  |  |  |  |  |  |  |  |  |  |  |  | 2 |  |  |  |  |  |  |  |  |  |  |  |  |  |  |  |  |  |  |  |  |  |  |  |  |  |  |  |  |  |  |  |  |  |  |  |  |  |  |
| ATC |  |  |  |  | 1 |  | 1 |  |  |  |  |  |  |  | 1 |  |  |  |  |  |  |  |  |  |  |  |  |  |  |  |  |  |  |  |  |  |  |  |  |  |  |  |  |  |  |  |  |  |  |  |  |  |  |  |  |  |
| ATG | 2 | 1 |  |  | 5 |  |  |  |  |  |  |  |  |  |  |  |  |  |  |  |  |  |  |  |  |  |  |  |  |  |  |  |  |  |  |  |  |  |  |  |  |  |  |  |  | 1 |  |  |  |  |  | 2 |  |  |  |  |
| ATT |  |  |  |  |  |  |  |  |  |  |  |  |  |  |  |  |  |  |  |  |  |  |  |  |  |  |  |  |  |  |  |  |  |  |  |  |  |  |  |  |  |  |  |  |  |  |  |  |  |  |  |  |  |  |  |  |
| CAA | 3 |  |  |  |  |  |  |  |  |  |  |  |  |  |  |  |  |  |  |  |  |  |  |  |  |  |  |  |  |  |  |  |  |  |  |  |  |  |  |  |  |  |  |  |  |  |  |  |  |  |  |  |  |  |  |  |
| CAC |  |  |  |  |  |  |  |  |  |  |  |  |  |  |  |  |  |  |  |  |  |  |  |  |  | 1 |  |  |  |  |  |  |  |  |  |  |  |  |  |  |  |  |  |  |  |  |  |  |  |  |  |  |  |  |  |  |
| CAG | 3 |  |  |  |  |  |  |  |  |  |  |  |  |  |  |  |  |  |  |  |  |  |  |  |  |  |  |  |  |  |  |  |  |  |  |  |  |  |  |  |  |  |  |  |  |  |  |  |  |  |  |  |  |  |  |  |
| CAT |  |  |  |  |  |  |  |  |  |  |  |  |  |  |  |  |  |  | 4 |  |  |  |  |  |  | 3 |  |  |  |  |  |  |  |  |  |  |  |  |  |  |  |  |  |  |  |  |  |  |  |  |  |  |  |  |  |  |
| CCA | 1 |  |  |  |  |  |  |  |  |  |  |  |  |  |  |  |  |  |  |  |  |  |  |  |  |  |  |  |  |  |  |  |  |  |  |  |  |  |  |  |  |  |  |  |  |  |  |  |  |  |  |  |  |  |  |  |
| CCT |  |  |  |  |  |  |  |  |  |  |  |  |  |  |  |  |  |  |  |  |  |  |  |  |  |  |  |  |  |  |  |  |  |  |  |  |  |  |  |  |  |  |  |  |  |  |  |  |  |  |  |  |  |  |  |  |
| CGA | 2 |  |  |  |  |  |  |  |  |  |  |  |  |  |  |  |  |  |  |  |  |  |  |  |  | 2 |  |  |  |  |  |  |  |  |  |  |  |  |  |  |  |  |  |  |  |  |  |  |  |  |  |  |  |  |  |  |
| CGC | 1 |  |  |  |  |  |  |  |  |  |  |  |  |  |  |  |  |  |  |  |  |  |  |  |  |  |  |  |  |  |  |  |  |  |  |  |  |  |  |  |  |  |  |  |  |  |  |  |  |  |  |  |  |  |  |  |
| CTA | 1 |  |  |  |  |  |  |  |  |  |  |  |  |  |  |  |  |  |  |  |  |  |  |  |  |  |  |  |  |  |  |  |  |  |  |  |  |  |  |  |  |  |  |  |  |  |  |  |  |  |  |  |  |  |  |  |
| CTC | 1 |  |  |  |  |  |  |  |  |  |  |  |  |  |  |  |  |  |  | 1 |  |  |  |  |  |  |  |  |  |  |  |  |  |  |  |  |  |  |  |  |  |  |  |  |  |  |  |  |  |  |  |  |  |  |  |  |
| CTG |  |  |  |  |  |  |  |  |  |  |  |  |  |  |  |  |  |  |  |  |  |  |  |  |  |  |  |  |  |  |  |  |  |  |  |  |  |  |  |  |  |  |  |  |  |  |  |  |  |  |  |  |  |  |  |  |
| CTT | 1 |  |  |  |  |  |  |  |  |  |  |  |  |  |  |  |  |  |  |  |  |  |  |  |  |  |  |  |  |  |  |  |  |  |  |  |  |  |  |  |  |  |  |  |  |  |  |  |  |  |  |  |  |  |  |  |
| GAA | 4 |  |  |  |  |  |  |  |  |  |  |  |  |  |  |  |  |  |  |  |  |  |  |  |  |  |  |  |  |  |  |  |  |  |  |  |  |  |  |  |  |  |  |  |  |  |  |  |  |  |  |  |  |  |  |  |
| GAC | 1 |  |  |  |  |  |  |  |  |  |  |  |  |  |  |  |  |  |  |  |  |  |  |  |  |  |  | 1 |  |  |  |  |  |  |  |  |  |  |  |  |  |  |  |  |  |  |  |  |  |  |  |  |  |  |  |  |
| GAG | 1 |  |  |  |  |  |  |  |  |  |  |  |  |  |  |  |  |  |  |  |  |  |  |  |  |  |  |  |  |  |  |  |  |  |  |  |  |  |  |  |  |  |  |  |  |  |  |  |  |  |  |  |  |  |  |  |
| GAT | 3 |  |  |  |  |  |  |  |  |  |  |  |  |  |  |  |  |  |  |  |  |  |  |  |  |  |  |  |  |  |  |  |  |  |  |  |  |  |  |  |  |  |  |  |  |  |  |  |  |  |  |  |  |  |  |  |
| GCA | 5 |  |  |  |  |  |  |  |  |  |  |  |  |  |  |  |  |  |  |  |  |  |  |  |  |  |  |  |  |  |  |  |  |  |  |  |  |  |  |  | 2 | 2 |  |  |  |  | 1 |  |  |  |  |  |  |  |  |  |
| GCC | 1 |  |  |  |  |  |  |  |  |  |  |  |  |  |  |  |  |  |  |  |  |  |  |  |  |  |  |  |  |  |  |  |  |  |  |  |  |  |  |  |  |  |  |  |  |  |  |  |  |  |  |  |  |  |  |  |
| GCG |  |  |  |  |  |  |  |  |  |  |  |  |  |  |  |  |  |  |  |  |  |  |  |  |  |  |  |  |  |  | 1 |  |  |  |  |  |  |  |  |  |  |  |  |  |  |  |  |  |  |  |  |  |  |  |  |  |
| GCT | 1 |  |  |  |  |  |  |  |  |  |  |  |  |  |  |  |  |  |  |  |  |  |  |  |  |  |  |  |  |  |  |  |  |  |  |  |  |  |  |  |  |  |  |  |  |  |  |  |  |  |  |  |  |  |  |  |
| GGA |  |  |  |  |  |  |  |  |  |  |  |  |  |  |  |  |  |  |  |  |  |  |  |  |  |  |  |  |  |  |  |  |  |  |  | 3 |  |  | 1 |  |  |  |  |  |  |  |  |  |  |  |  |  |  |  |  |  |
| GGC | 1 |  |  |  |  |  |  |  |  |  |  |  |  |  |  |  |  |  |  |  |  |  |  |  |  |  |  |  |  |  |  |  |  |  |  |  |  |  |  |  |  |  |  |  |  |  |  |  |  |  |  |  |  |  |  |  |
| GGG | 2 |  |  |  |  |  |  |  |  |  |  |  |  |  |  |  |  |  |  |  |  |  |  |  |  |  |  |  |  |  |  |  |  |  |  |  |  |  | 2 |  |  |  |  |  |  |  |  |  |  |  |  |  |  |  |  |  |
| GGT | 3 |  |  |  |  |  |  |  |  |  |  |  |  |  |  |  |  |  |  |  |  |  |  |  |  |  |  | 2 |  |  |  |  |  |  |  |  |  |  |  |  |  |  |  |  |  |  |  |  |  |  |  |  |  |  |  |  |
| GTA | 1 |  |  |  |  |  |  |  |  |  |  |  |  |  |  |  |  |  |  |  |  |  |  |  |  |  |  |  |  |  |  |  |  |  |  |  |  |  |  |  |  |  |  |  |  |  |  |  |  |  |  |  |  |  |  |  |
| GTG |  |  |  |  |  |  |  |  |  |  |  |  |  |  |  |  |  |  |  |  |  |  |  |  |  |  |  |  |  |  |  |  |  |  |  |  |  |  |  |  |  |  |  |  |  |  |  |  |  |  |  |  |  |  |  |  |
| GTT | 3 |  |  |  |  |  |  |  |  |  |  |  |  |  |  |  |  |  |  |  |  |  |  |  |  |  |  |  |  |  |  |  |  |  |  |  |  |  |  |  |  |  |  |  |  |  |  |  |  |  |  |  |  |  |  |  |
| TAA |  |  |  |  |  |  |  |  |  |  |  |  |  |  |  |  |  |  |  |  |  |  |  |  |  |  |  |  |  |  |  |  |  |  |  |  |  |  |  |  |  |  |  |  |  |  |  |  |  |  |  |  |  |  |  |  |
| TAC |  |  |  |  |  |  |  |  |  |  |  |  |  |  |  |  |  |  |  |  |  |  |  |  |  |  |  |  |  |  |  |  |  |  |  |  |  |  |  |  |  |  |  |  |  |  |  |  |  |  |  |  |  |  |  |  |
| TAG |  |  |  |  |  |  |  |  |  |  |  |  |  |  |  |  |  |  |  |  |  |  |  |  |  |  |  |  |  |  |  |  |  |  |  |  |  |  |  |  |  |  |  |  |  |  |  |  |  |  |  |  |  |  |  |  |
| TAT | 3 |  |  |  |  |  |  |  |  |  |  |  |  |  |  |  |  |  |  |  |  |  |  |  |  |  |  |  |  |  |  |  |  |  |  |  |  |  |  |  |  |  |  |  |  |  |  |  |  |  |  |  |  |  |  |  |
| TCA | 1 |  |  |  |  |  |  |  |  |  |  |  |  |  |  |  |  |  |  |  |  |  |  |  |  |  |  |  |  |  |  |  |  |  |  |  |  |  |  |  |  |  |  |  |  |  |  |  | 1 |  |  |  |  |  |  |  |
| TCC |  |  |  |  |  |  |  |  |  |  |  |  |  |  |  |  |  |  |  |  |  |  |  |  |  |  |  |  |  |  |  |  |  |  |  |  |  |  |  |  |  |  |  |  |  |  |  |  |  |  |  |  |  |  |  |  |
| TCT | 3 |  |  |  |  |  |  |  |  |  |  |  |  |  |  |  |  |  |  |  |  |  |  |  |  |  |  |  |  |  |  |  |  |  |  |  |  |  |  |  |  | 2 |  |  |  |  |  |  |  | 1 |  |  |  | 1 |  |  |
| TGA |  |  |  |  |  |  |  |  |  |  |  |  |  |  |  |  |  |  |  |  |  |  |  |  |  |  |  |  |  |  |  |  |  |  |  |  |  |  |  |  |  |  |  |  |  |  |  |  |  |  |  |  |  |  |  |  |
| TGG |  |  |  |  |  |  |  |  |  |  |  |  |  |  |  |  |  |  |  |  |  |  |  |  |  |  |  |  |  |  |  |  |  |  |  |  |  |  |  |  |  |  |  |  |  |  |  |  |  |  |  |  |  |  |  |  |
| TGT | 1 |  |  |  |  |  |  |  |  |  |  |  |  |  |  |  |  |  |  |  |  |  |  |  |  |  |  |  |  |  |  |  |  |  |  |  |  |  |  |  |  |  |  |  |  |  |  |  |  |  |  |  |  |  |  |  |
| TTA | 1 |  |  |  |  |  |  |  |  |  |  |  |  |  |  |  |  |  |  |  |  |  |  |  |  |  |  |  |  |  |  |  |  |  |  |  |  |  |  |  |  |  |  |  |  |  |  |  |  |  |  |  |  |  |  |  |
| TTC |  |  |  |  |  |  |  |  |  |  |  |  |  |  |  |  |  |  |  |  |  |  |  |  |  |  |  |  |  |  |  |  |  |  |  |  |  |  |  |  |  |  |  |  |  |  |  |  |  |  |  |  |  |  |  |  |
| TTG |  |  |  |  |  |  |  |  |  |  |  |  |  |  |  |  |  |  |  |  |  |  |  |  |  |  |  |  |  |  |  |  |  |  |  |  |  |  |  |  |  |  |  |  |  |  |  |  |  |  |  |  |  |  |  | 2 |
| TTT | 1 |  |  |  |  |  |  |  |  |  |  |  |  |  |  |  |  |  |  |  |  |  |  |  |  |  |  |  |  |  |  |  |  |  |  |  |  |  |  |  |  |  |  |  |  |  |  |  |  |  |  |  |  |  |  |  |


---


 **Amino acid changes**

How to read this table:   
- Rows are reference amino acids and columns are changed amino acids. E.g. Row 'A' column 'E' indicates how many 'A' amino acids have been replaced by 'E' amino acids.  
- Red background colors indicate that more changes happened (heat-map).  
- Diagonals are indicated using grey background color   
- WARNING: This table may include different translation codon tables (e.g. mamalian DNA and mitochondrial DNA).

|  | \* | - | ? | A | C | D | E | F | G | H | I | K | L | M | N | P | Q | R | S | T | V | W | Y |
| --- | --- | --- | --- | --- | --- | --- | --- | --- | --- | --- | --- | --- | --- | --- | --- | --- | --- | --- | --- | --- | --- | --- | --- |
| \* |  |  |  |  |  |  |  |  |  |  |  |  |  |  |  |  |  |  |  |  |  |  |  |
| - | 61 |  | 44 |  |  | 7 | 21 | 35 | 17 | 1 | 34 | 19 | 58 |  | 43 | 4 | 19 | 7 | 37 | 9 | 25 | 4 | 12 |
| ? |  |  |  |  |  |  |  |  |  |  |  |  |  |  |  |  |  |  |  |  |  |  |  |
| A |  | 7 |  |  |  | 1 |  |  |  |  |  |  |  |  |  |  |  |  | 1 |  | 4 |  |  |
| C |  | 1 |  |  |  |  |  |  |  |  |  |  |  |  |  |  |  |  |  |  |  |  |  |
| D |  | 4 |  |  |  |  | 1 |  |  |  |  |  |  |  |  |  |  |  |  |  |  |  |  |
| E |  | 5 |  |  |  |  |  |  |  |  |  |  |  |  |  |  |  |  |  |  |  |  |  |
| F |  | 1 |  |  |  |  |  |  |  |  |  |  |  |  |  |  |  |  |  |  |  |  |  |
| G |  | 6 |  |  |  |  | 2 |  | 6 |  |  |  |  |  |  |  |  |  |  |  |  |  |  |
| H |  |  |  |  |  |  |  |  |  | 4 |  |  | 4 |  |  |  |  |  |  |  |  |  |  |
| I |  |  |  |  |  |  |  |  |  |  | 1 |  |  |  | 1 |  |  |  |  | 1 |  |  |  |
| K |  | 4 |  |  | 3 |  |  |  |  |  |  |  |  |  |  |  |  | 3 |  |  |  |  |  |
| L |  | 4 |  |  |  |  |  | 2 |  |  |  |  |  |  |  | 1 |  |  |  |  |  |  |  |
| M |  | 2 |  |  | 2 |  |  |  |  |  |  | 1 |  |  | 5 |  |  |  |  |  |  |  | 1 |
| N |  |  |  |  |  |  |  |  |  |  |  |  |  |  | 1 |  |  |  |  |  |  |  |  |
| P |  | 1 |  |  |  |  |  |  |  |  |  |  |  |  |  |  |  |  |  |  |  |  |  |
| Q |  | 6 |  |  |  |  |  |  |  |  |  |  |  |  |  |  |  |  |  |  |  |  |  |
| R |  | 5 |  |  |  |  |  |  |  |  |  |  | 2 |  |  |  |  |  |  |  |  |  |  |
| S | 1 | 5 |  |  |  |  |  | 1 |  |  |  |  |  |  |  |  | 2 |  | 1 |  | 2 |  |  |
| T |  | 6 |  |  |  |  |  |  |  |  |  |  |  |  |  |  |  |  |  |  |  |  |  |
| V |  | 4 |  |  |  |  |  |  |  |  |  |  |  |  |  |  |  |  |  |  |  |  |  |
| W |  |  |  |  |  |  |  |  |  |  |  |  |  |  |  |  |  |  |  |  |  |  |  |
| Y |  | 3 |  |  |  |  |  |  |  |  |  |  |  |  |  |  |  |  |  |  |  |  |  |


---


 **Variants by chromosome**

```
		  

		1, Position,0,1000000,2000000,3000000,4000000,5000000,6000000,7000000,8000000,9000000,10000000,11000000,12000000,13000000,14000000,15000000,16000000,17000000,18000000,19000000,20000000,21000000,22000000,23000000,24000000,25000000,26000000,27000000,28000000,29000000,30000000
1,Count,14,10,19,12,10,6,11,12,7,10,14,12,14,15,7,27,12,12,13,5,16,10,9,20,17,16,10,9,6,16,6

	
```

```
		  

		2, Position,0,100000,200000,300000,400000,500000,600000,700000,800000,900000,1000000,1100000,1200000,1300000,1400000,1500000,1600000,1700000,1800000,1900000,2000000,2100000,2200000,2300000,2400000,2500000,2600000,2700000,2800000,2900000,3000000,3100000,3200000,3300000,3400000,3500000,3600000,3700000,3800000,3900000,4000000,4100000,4200000,4300000,4400000,4500000,4600000,4700000,4800000,4900000,5000000,5100000,5200000,5300000,5400000,5500000,5600000,5700000,5800000,5900000,6000000,6100000,6200000,6300000,6400000,6500000,6600000,6700000,6800000,6900000,7000000,7100000,7200000,7300000,7400000,7500000,7600000,7700000,7800000,7900000,8000000,8100000,8200000,8300000,8400000,8500000,8600000,8700000,8800000,8900000,9000000,9100000,9200000,9300000,9400000,9500000,9600000,9700000,9800000,9900000,10000000,10100000,10200000,10300000,10400000,10500000,10600000,10700000,10800000,10900000,11000000,11100000,11200000,11300000,11400000,11500000,11600000,11700000,11800000,11900000,12000000,12100000,12200000,12300000,12400000,12500000,12600000,12700000,12800000,12900000,13000000,13100000,13200000,13300000,13400000,13500000,13600000,13700000,13800000,13900000,14000000,14100000,14200000,14300000,14400000,14500000,14600000,14700000,14800000,14900000,15000000,15100000,15200000,15300000,15400000,15500000,15600000,15700000,15800000,15900000,16000000,16100000,16200000,16300000,16400000,16500000,16600000,16700000,16800000,16900000,17000000,17100000,17200000,17300000,17400000,17500000,17600000,17700000,17800000,17900000,18000000,18100000,18200000,18300000,18400000,18500000,18600000,18700000,18800000,18900000,19000000,19100000,19200000,19300000,19400000,19500000,19600000
2,Count,0,1,0,1,2,1,3,2,1,0,0,0,1,2,1,1,0,1,2,2,0,0,0,0,1,2,2,4,1,1,3,0,0,1,1,0,4,3,0,2,2,0,0,2,1,0,1,3,2,1,3,0,0,1,1,0,1,0,1,3,1,1,1,2,0,1,3,3,1,1,1,0,2,3,0,0,2,0,1,0,2,1,2,2,2,0,0,1,0,2,3,1,0,0,3,2,0,2,1,2,0,1,1,0,0,2,2,0,1,1,3,1,0,4,0,1,0,1,4,1,3,2,2,3,1,3,3,2,1,12,1,0,2,2,0,3,2,0,1,0,2,2,1,2,1,2,1,1,2,1,2,3,1,0,2,2,3,5,1,1,1,0,2,1,0,0,0,1,1,0,2,1,3,2,0,1,0,0,0,2,1,1,0,0,3,0,0,1,0,0,0,3,4,3,3,0,2

	
```

```
		  

		3, Position,0,100000,200000,300000,400000,500000,600000,700000,800000,900000,1000000,1100000,1200000,1300000,1400000,1500000,1600000,1700000,1800000,1900000,2000000,2100000,2200000,2300000,2400000,2500000,2600000,2700000,2800000,2900000,3000000,3100000,3200000,3300000,3400000,3500000,3600000,3700000,3800000,3900000,4000000,4100000,4200000,4300000,4400000,4500000,4600000,4700000,4800000,4900000,5000000,5100000,5200000,5300000,5400000,5500000,5600000,5700000,5800000,5900000,6000000,6100000,6200000,6300000,6400000,6500000,6600000,6700000,6800000,6900000,7000000,7100000,7200000,7300000,7400000,7500000,7600000,7700000,7800000,7900000,8000000,8100000,8200000,8300000,8400000,8500000,8600000,8700000,8800000,8900000,9000000,9100000,9200000,9300000,9400000,9500000,9600000,9700000,9800000,9900000,10000000,10100000,10200000,10300000,10400000,10500000,10600000,10700000,10800000,10900000,11000000,11100000,11200000,11300000,11400000,11500000,11600000,11700000,11800000,11900000,12000000,12100000,12200000,12300000,12400000,12500000,12600000,12700000,12800000,12900000,13000000,13100000,13200000,13300000,13400000,13500000,13600000,13700000,13800000,13900000,14000000,14100000,14200000,14300000,14400000,14500000,14600000,14700000,14800000,14900000,15000000,15100000,15200000,15300000,15400000,15500000,15600000,15700000,15800000,15900000,16000000,16100000,16200000,16300000,16400000,16500000,16600000,16700000,16800000,16900000,17000000,17100000,17200000,17300000,17400000,17500000,17600000,17700000,17800000,17900000,18000000,18100000,18200000,18300000,18400000,18500000,18600000,18700000,18800000,18900000,19000000,19100000,19200000,19300000,19400000,19500000,19600000,19700000,19800000,19900000,20000000,20100000,20200000,20300000,20400000,20500000,20600000,20700000,20800000,20900000,21000000,21100000,21200000,21300000,21400000,21500000,21600000,21700000,21800000,21900000,22000000,22100000,22200000,22300000,22400000,22500000,22600000,22700000,22800000,22900000,23000000,23100000,23200000,23300000,23400000
3,Count,2,1,3,2,1,4,1,1,1,2,0,1,1,1,1,2,0,2,1,2,1,1,0,0,2,1,0,1,1,0,0,1,2,1,0,1,1,3,0,0,0,0,1,6,1,1,1,0,1,1,2,1,3,1,1,0,1,0,3,4,0,0,0,3,2,3,2,0,0,3,2,2,1,0,1,1,0,1,2,2,1,2,1,2,4,0,4,2,0,2,3,0,0,1,4,0,1,1,2,0,1,0,1,0,0,2,1,3,1,3,2,0,1,0,0,1,1,0,0,1,0,0,3,2,1,4,0,0,0,1,1,1,0,1,0,28,1,8,18,1,0,5,25,3,5,2,0,0,0,0,0,6,4,0,10,1,2,1,0,1,0,2,2,2,0,3,1,1,1,0,0,0,0,0,1,0,1,0,1,2,0,0,1,1,1,0,3,2,3,0,0,3,1,1,2,3,0,0,0,6,1,3,2,0,0,4,0,1,1,1,2,1,0,4,3,1,2,1,0,2,3,1,0,1,2,0,0,0,0,0,0,0,0,2,0

	
```

```
		  

		4, Position,0,100000,200000,300000,400000,500000,600000,700000,800000,900000,1000000,1100000,1200000,1300000,1400000,1500000,1600000,1700000,1800000,1900000,2000000,2100000,2200000,2300000,2400000,2500000,2600000,2700000,2800000,2900000,3000000,3100000,3200000,3300000,3400000,3500000,3600000,3700000,3800000,3900000,4000000,4100000,4200000,4300000,4400000,4500000,4600000,4700000,4800000,4900000,5000000,5100000,5200000,5300000,5400000,5500000,5600000,5700000,5800000,5900000,6000000,6100000,6200000,6300000,6400000,6500000,6600000,6700000,6800000,6900000,7000000,7100000,7200000,7300000,7400000,7500000,7600000,7700000,7800000,7900000,8000000,8100000,8200000,8300000,8400000,8500000,8600000,8700000,8800000,8900000,9000000,9100000,9200000,9300000,9400000,9500000,9600000,9700000,9800000,9900000,10000000,10100000,10200000,10300000,10400000,10500000,10600000,10700000,10800000,10900000,11000000,11100000,11200000,11300000,11400000,11500000,11600000,11700000,11800000,11900000,12000000,12100000,12200000,12300000,12400000,12500000,12600000,12700000,12800000,12900000,13000000,13100000,13200000,13300000,13400000,13500000,13600000,13700000,13800000,13900000,14000000,14100000,14200000,14300000,14400000,14500000,14600000,14700000,14800000,14900000,15000000,15100000,15200000,15300000,15400000,15500000,15600000,15700000,15800000,15900000,16000000,16100000,16200000,16300000,16400000,16500000,16600000,16700000,16800000,16900000,17000000,17100000,17200000,17300000,17400000,17500000,17600000,17700000,17800000,17900000,18000000,18100000,18200000,18300000,18400000,18500000
4,Count,4,0,1,2,2,0,1,1,0,0,1,0,1,0,1,0,0,0,1,1,4,1,1,1,2,0,0,0,0,1,2,3,0,0,3,4,0,0,1,15,5,1,1,1,0,0,0,6,0,0,0,0,0,0,0,1,2,0,1,0,4,2,3,0,1,2,2,0,0,0,1,1,2,1,0,0,1,1,1,0,0,1,0,3,0,1,2,0,1,6,3,3,0,0,4,0,0,2,1,0,0,0,2,2,1,0,2,1,2,1,1,1,1,0,0,1,3,0,5,1,0,2,3,3,0,1,1,1,1,3,1,0,0,0,1,0,3,1,2,2,0,0,2,0,5,2,2,2,0,0,1,0,1,1,1,0,1,0,1,0,0,2,2,1,1,1,1,1,0,0,0,0,1,4,3,0,1,1,1,0,1,1,3,2,2,1

	
```

```
		  

		5, Position,0,100000,200000,300000,400000,500000,600000,700000,800000,900000,1000000,1100000,1200000,1300000,1400000,1500000,1600000,1700000,1800000,1900000,2000000,2100000,2200000,2300000,2400000,2500000,2600000,2700000,2800000,2900000,3000000,3100000,3200000,3300000,3400000,3500000,3600000,3700000,3800000,3900000,4000000,4100000,4200000,4300000,4400000,4500000,4600000,4700000,4800000,4900000,5000000,5100000,5200000,5300000,5400000,5500000,5600000,5700000,5800000,5900000,6000000,6100000,6200000,6300000,6400000,6500000,6600000,6700000,6800000,6900000,7000000,7100000,7200000,7300000,7400000,7500000,7600000,7700000,7800000,7900000,8000000,8100000,8200000,8300000,8400000,8500000,8600000,8700000,8800000,8900000,9000000,9100000,9200000,9300000,9400000,9500000,9600000,9700000,9800000,9900000,10000000,10100000,10200000,10300000,10400000,10500000,10600000,10700000,10800000,10900000,11000000,11100000,11200000,11300000,11400000,11500000,11600000,11700000,11800000,11900000,12000000,12100000,12200000,12300000,12400000,12500000,12600000,12700000,12800000,12900000,13000000,13100000,13200000,13300000,13400000,13500000,13600000,13700000,13800000,13900000,14000000,14100000,14200000,14300000,14400000,14500000,14600000,14700000,14800000,14900000,15000000,15100000,15200000,15300000,15400000,15500000,15600000,15700000,15800000,15900000,16000000,16100000,16200000,16300000,16400000,16500000,16600000,16700000,16800000,16900000,17000000,17100000,17200000,17300000,17400000,17500000,17600000,17700000,17800000,17900000,18000000,18100000,18200000,18300000,18400000,18500000,18600000,18700000,18800000,18900000,19000000,19100000,19200000,19300000,19400000,19500000,19600000,19700000,19800000,19900000,20000000,20100000,20200000,20300000,20400000,20500000,20600000,20700000,20800000,20900000,21000000,21100000,21200000,21300000,21400000,21500000,21600000,21700000,21800000,21900000,22000000,22100000,22200000,22300000,22400000,22500000,22600000,22700000,22800000,22900000,23000000,23100000,23200000,23300000,23400000,23500000,23600000,23700000,23800000,23900000,24000000,24100000,24200000,24300000,24400000,24500000,24600000,24700000,24800000,24900000,25000000,25100000,25200000,25300000,25400000,25500000,25600000,25700000,25800000,25900000,26000000,26100000,26200000,26300000,26400000,26500000,26600000,26700000,26800000,26900000
5,Count,1,4,1,0,2,3,0,1,2,3,1,0,4,1,0,0,3,1,1,5,0,0,2,2,3,3,1,1,0,0,0,2,0,0,0,1,0,0,1,1,1,1,0,0,0,3,1,1,2,1,1,1,2,1,1,0,1,4,0,3,0,2,3,1,4,2,0,0,0,0,1,1,2,0,1,3,2,2,1,0,0,1,1,1,1,1,1,3,1,1,0,1,0,1,1,1,1,2,2,1,0,0,2,2,1,2,1,1,1,2,1,0,0,0,0,0,0,16,2,2,6,2,3,0,0,1,0,0,4,0,0,1,2,1,1,1,0,2,0,0,1,0,1,0,0,0,0,3,2,1,1,0,1,3,0,0,0,0,0,2,1,0,1,2,0,2,1,1,2,0,2,1,1,0,1,0,3,2,2,2,1,0,2,1,1,0,2,4,0,1,0,1,1,1,1,0,0,3,1,2,1,0,3,0,2,1,0,1,3,1,0,0,1,4,2,2,0,3,2,0,1,2,0,3,2,1,3,0,7,1,0,1,1,0,0,1,1,2,1,1,0,1,0,3,3,0,2,1,1,2,0,2,2,4,1,4,2,0,1,3,1,2,2,3,2,0,2,0,1,1

	
```


---

 **Details by gene** 

**Here** you can find a tab-separated table.
